# High-Throughput Tabular Data Processor – Platform independent graphical tool for processing large data sets

**DOI:** 10.1371/journal.pone.0192858

**Published:** 2018-02-12

**Authors:** Piotr Madanecki, Magdalena Bałut, Patrick G. Buckley, J. Renata Ochocka, Rafał Bartoszewski, David K. Crossman, Ludwine M. Messiaen, Arkadiusz Piotrowski

**Affiliations:** 1 Department of Biology and Pharmaceutical Botany, Medical University of Gdansk, Gdansk, Poland; 2 Molecular Pathology Laboratory, Beaumont Hospital, Dublin, Ireland; 3 Heflin Center for Genomic Science, Department of Genetics, University of Alabama at Birmingham, Birmingham, Alabama, United States of America; 4 Medical Genomics Laboratory, Department of Genetics, University of Alabama at Birmingham, Birmingham, Alabama, United States of America; Roswell Park Cancer Institute, UNITED STATES

## Abstract

High-throughput technologies generate considerable amount of data which often requires bioinformatic expertise to analyze. Here we present High-Throughput Tabular Data Processor (HTDP), a platform independent Java program. HTDP works on any character-delimited column data (e.g. BED, GFF, GTF, PSL, WIG, VCF) from multiple text files and supports merging, filtering and converting of data that is produced in the course of high-throughput experiments. HTDP can also utilize itemized sets of conditions from external files for complex or repetitive filtering/merging tasks. The program is intended to aid global, real-time processing of large data sets using a graphical user interface (GUI). Therefore, no prior expertise in programming, regular expression, or command line usage is required of the user. Additionally, no *a priori* assumptions are imposed on the internal file composition. We demonstrate the flexibility and potential of HTDP in real-life research tasks including microarray and massively parallel sequencing, i.e. identification of disease predisposing variants in the next generation sequencing data as well as comprehensive concurrent analysis of microarray and sequencing results. We also show the utility of HTDP in technical tasks including data merge, reduction and filtering with external criteria files. HTDP was developed to address functionality that is missing or rudimentary in other GUI software for processing character-delimited column data from high-throughput technologies. Flexibility, in terms of input file handling, provides long term potential functionality in high-throughput analysis pipelines, as the program is not limited by the currently existing applications and data formats. HTDP is available as the Open Source software (https://github.com/pmadanecki/htdp).

## Introduction

High-throughput technologies, e.g. microarrays and massively parallel sequencing, have become standard tools in genetics. Widespread utilization of these technologies is reflected by the substantial amount of data which is generated during these experiments. As a result, data analysis from such experiments is a challenging and complex task. Consequently, new software solutions are constantly being developed to handle information inflow and management of genomic data. A widening range of available software inherently leads to cross-compatibility issues in terms of data file formats. Several formats based on tabular text files with delimiters (e.g. BED, GFF, GTF, WIG, VCF), are widely used for exchange and storage of microarray data (http://www.sanger.ac.uk/resources/software/gff/spec.html; http://mblab.wustl.edu/GTF22.html; http://genome.ucsc.edu/FAQ/FAQformat.html) [1]. In general, these file formats denote genomic coordinates along with corresponding annotation data, which can include genomic features or experimental values such as normalized expression, allele zygosity, ChIP enrichment or DNA copy number status. However, format-specific or non-standard features can also be included in these data sets. For example, genetic variants resulting from massively parallel sequencing are usually stored in the Variant Call Format (VCF).The VCF was proposed by the 1000 Genomes Consortium which published format specification guidelines (http://genome.ucsc.edu/FAQ/FAQformat.html; http://www.1000genomes.org/wiki/analysis/variant-call-format/vcf-variant-call-format-version-42) [2,3]. However, VCF files produced by different programs often deviate from the canonical layout. This is due to flexibility assumed by the VCF definition. Specifically, the INFO field may contain a variety of user defined features which are not imposed by VCF specification as outlined by the 1000 Genomes Consortium (http://www.1000genomes.org/wiki/analysis/variant-call-format/vcf-variant-call-format-version-42) [2].

Non-standard entries in genomic data files, if not recognized, may lead to compatibility issues upon data exchange between different programs or services. Furthermore, the non-standard features are often ignored by other programs and constitute excess information which creates unnecessary burden in terms of data processing and storage.

To alleviate these problems several solutions can be used, including standard or specialized unix command line tools (e.g. grep, VCF-tools, BED-tools) [2,4], custom programming (e.g. Perl, Python scripts) [5,6], commercial software solutions and in some cases office spreadsheet software [2,4,7]. However, usability of these tools is limited to users with programming skills, or at least a basic understanding of regular expressions and command line user interface. At the same time, software based on Graphical User Interface (GUI) usually requires the purchase of a commercial license or does not provide sufficient functionality to handle high-throughput data.

**Fig 1:**
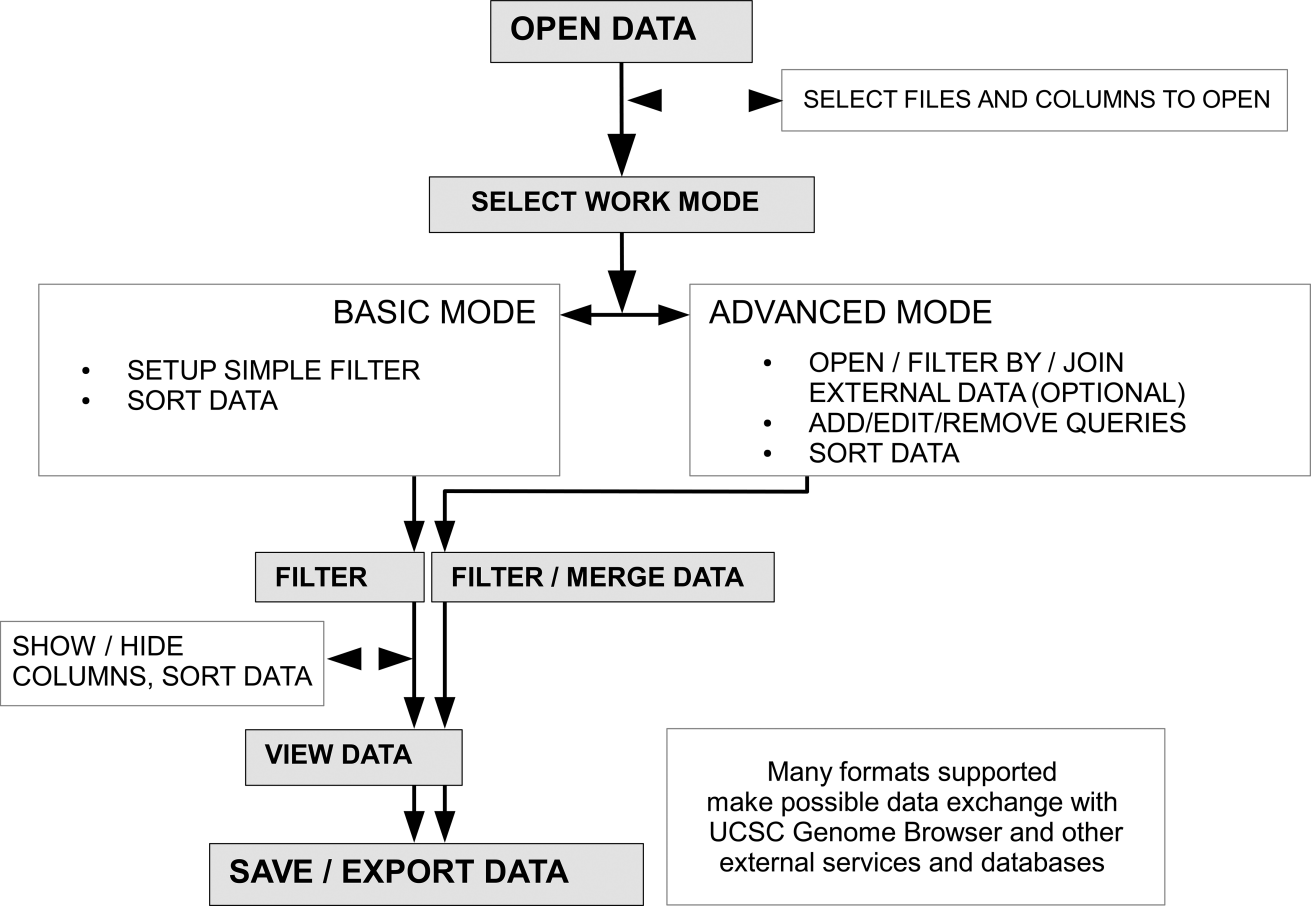
Typical HTDP workflow diagram with main features.

Here, we present High-Throughput Tabular Data Processor (HTDP), a platform independent Java program, which features filtering, converting and combining multiple data files that are produced by high-throughput technologies, e.g. microarray and massively parallel sequencing experiments. The program is intended to aid global, real-time processing of large data sets using GUI, therefore no prior expertise in programming, regular expression, or command line usage is required of the user. Furthermore, the GUI can be switched between the basic and advanced mode of operation. HTDP supports character-delimited text files, with no *a priori* assumptions imposed on the internal file composition. Importantly, different files with regards to format and content can be analyzed at the same time. HTDP provides unlimited filtering and data reduction capabilities, also using itemized filtering conditions from external files. Finally, the program can be used for conversion between different standard formats that are commonly used for high-throughput data, i.e. BED, GFF, GTF, WIG and VCF. Detailed description of program functions and usage is provided in the user manual (S1 File and https://github.com/pmadanecki/htdp) and typical workflow is presented in Fig 1.

## Results

HTDP was created to meet the demand for efficient GUI based processing of high-throughput data, with flexible support for multiple files and character-delimited formats (Table 1).

**Table 1:** Summary of the main HTDP features.

Feature	Note
Horizontal merging of files ^a^	Based on genome location (many locations formats and join options are supported); horizontal joining of the files based on one or multiple columns (max 4) is supported.
Vertical merging of files (by append) ^a^	The files may be in different formats. Columns with the same name are merged
Filtering data by list of values ^a^	
Operations on samples ^a^	A total of 1–4 user selectable columns indicate sample name. Filtering based on the other columns content that are present in user-defined percentage of samples is possible
Developed as java application	Platform independence—works on all supported architectures and operating systems without emulators
Flexible input file(s) format	Any character-delimited column data file(s) is accepted (including VCF with headers and non-canonical entries in the INFO field). User defined delimiting as well as commenting characters are allowed for input files. Missing data values are permitted and can be denoted by user-specified symbol. Column/row names are not mandatory and can be automatically assigned. Multiple files with different delimiters and column/row composition can be opened and combined in one data set (limited by available RAM^b^ or a maximum of 2 147 483 647 indexed elements in an array).
Many export formats	Output files are saved as tab delimited text files. Additionally, export/conversion to the following formats is supported: BED, BED detail, PSL, GFF, Personal Genome SNP, ENCODE RNA elements: BED6 + 3 scores, ENCODE narrowPeak: Narrow (or Point-Source) Peaks, ENCODE broadPeak: Broad Peaks (or Regions), ENCODE gappedPeak: Gapped Peaks (or Regions), ENCODE peptideMapping: BED6+4 detailed formats description: https://genome.ucsc.edu/FAQ/FAQformat.html

^a^ This feature is shown in Example 1 and is intended for illustrative purposes. This example contains sets of one stage tasks demonstrating the most useful and unique features of HTDP on simple artificial data set (S2 File).

^b^ Random Access Memory (RAM) requirement is c.a. 1GB RAM per 50MB of input file size, however the exact values may vary depending on the specific query composition and internal complexity of the input files. Sample test results and additional information are provided in the user manual (S1 File, page: 6).

Furthermore, HTDP was written for compatibility with UCSC Genome Browser tracks in terms of input and output files (http://genome.ucsc.edu). The UCSC Genome Browser tracks can be imported and used as the filtering criteria, while the resulting files can be exported from the HTDP in one of the formats which are used for the custom tracks. We successfully tested the program, performing real-life tasks to demonstrate the potential utility of HTDP.

### Identification of a disease predisposing variant in deep parallel sequencing data

We used HTDP in a high-throughput parallel sequencing study aimed at the identification of a gene predisposing to the inherited disorder of multiple schwannomas. The data analysis pipeline included variant calling with Platypus, followed by annotation with SeatlleSeq137 and shortlisting candidate variants with HTDP (http://www.well.ox.ac.uk/platypus) [8]. This analysis identified *LZTR1* as the disease predisposing gene [9]. Additionally, the HTDP was used to merge raw Platypus output with the SeattleSeq137 annotated variant list. The raw output from Platypus as well as other variant callers (e.g. SnpEff, GATK) contains detailed technical information on variant call quality [10–12]. This information is lost upon annotation with SeattleSeq. Combining selected data fields from the raw variant data with the corresponding entries in the annotated variant list facilitated downstream analysis, which reduced likely false positive calls. Sample input data along with the results of selective merge operation highlight this feature (Example 2 in S2 File).

### Filtering with external criteria files

HTDP provides advanced filtering capabilities based on external criteria files. The contents of the criteria file is flexible, however it should be provided in one of the supported formats containing genomic coordinates along with annotation features. Here we demonstrate how advanced filtering can be used to narrow down the list of candidate sequence alterations in a project aimed at the identification of disease causing variants. The first external criteria file used was the 1000 Genomes Project Phase 1 Paired-end Accessible Regions track from UCSC Genome Browser. Using this track we identified regions that may display an increased rate of false calls due to anomalous depth of coverage, or regions located within redundant sequences, resulting in low mapping quality. Next, using the 46-way evolutionary conservation track we selected variants at residues showing the highest evolutionary conservation (>0.85 according to phastCons method), thus of likely causal significance (Example 3 in S2 File). The latter feature is particularly useful for non-coding regions as variant annotation tools are usually deficient in *in silico* predictions for non-genic regions.

### Integrated analysis of DNA copy number and deep parallel sequencing data

Another feature of HTDP allows the parallel analysis of sequencing data and copy number variants (CNV) from microarray based comparative genomic hybridization experiments (aCGH). Point mutations from the targeted resequencing study of driver mutations in breast cancer (VCF data) were compared to CNV status (normalized log_2_ ratios, tab delimited data) in the same set of clinical samples. This provided comprehensive information on allelic status, including inactivation via simple nucleotide mutations or loss of heterezygosity due to deletion, as well as possible dosage effect caused by copy number gain. Finally, the program output was exported to BED custom tracks for convenient visualization in the UCSC Genome Browser (Example 4 in S2 File).

### Reduction of data size

The multistep analysis, involving the merging of data from different sources, yields results which are extremely rich in terms of the number and complexity of annotated features. While rich annotation is generally advantageous for versatility of downstream processing, it may prove redundant for data presentation purposes. Particularly, web services such as UCSC Genome Browser or Ensembl permit upload or linking user data. User data containing excessive information may easily exceed the allowed size limit (e.g. 50 MB for Ensembl) or, if no limit is imposed, excessive information increases network traffic and slows down data presentation. Additionally, data with rich annotation may not comply with the format definition and cause issues upon importing or presentation. Therefore, we incorporated a data reduction functionality in HTDP. This feature was tested by simplifying the 1000 Genomes Project Phase 1 variants set [13]. We also reduced GFF data containing aCGH results along with full information on oligonucleotide probe properties and sequences, which was unnecessary for the presentation of normalized copy number ratios (Example 5 in S2 File).

## Discussion

HTDP was developed based on the need to analyze high-throughput, e.g. genomic, data in a versatile, efficient manner. The program is not intended to replace other GUI software with partially overlapping functions, such as VarSifter or Tablebutler [14,15]. VarSifter is a convenient, but specialized tool for targeted analysis of genetic variants, solely in deep parallel sequencing data. While VarSifter supports VCF files, it cannot use tab delimited files as input at the same time. Also, advanced data filtering in VarSifter requires knowledge of regular expressions, and using advanced filtering criteria from external sources is supported only for two predefined file formats. On the other hand, Tablebutler is designed for comprehensive processing of tab delimited files [14]. Tablebutler is a file parser, and although its memory requirements are moderate, it lacks real-time global data analysis capabilities. However, its major disadvantages are the lack of support for VCF and the lack of filtering functionality based on genomic coordinates. Tablebutler is therefore dependent on third party software to perform this function which impacts on the ease of use of the application as at least two external programs are required for conversion between VCF and tab delimited text file.

HTDP overcomes these drawbacks by integrating complex data analysis task in a single user-defined pipeline. However, this approach implies extensive use of allocated random access memory (RAM). Hence, availability of sufficient amount of RAM is the main limitation of HTDP, specifically when complex input files of considerable size are subjected to compound queries (Table 1, S1 File, page: 6).

HTDP is designed for non-expert users operating a basic GUI. At the same time, in the advanced mode, the program ensures the flexibility that is required to carry out complex data processing tasks, using input files of different formats, enabling the set up of multiple filters as well as providing data merge, reduction and conversion capabilities. Although the program was developed to handle high-throughput genomic data in principle, its flexibility allows the use of HTDP in other areas of high-throughput analysis as it is not limited by currently existing applications and data formats. Finally, HTDP is continually developed by the authors, hosted on two independent public repositories (https://github.com/pmadanecki/htdp and https://sourceforge.net/projects/htdp/) and is available as Open Source software which facilitates its future development and possible contribution to community based projects.

## Methods

HTDP was written in Java using the NetBeans IDE (http://netbeans.org), utilizing the modular architecture and windowing system provided by the NetBeans Platform. Nullsoft Scriptable Install System (NSIS, https://sourceforge.net/projects/nsis/) was used to generate windows installer. NSIS is licensed as zlib/libpng licensed software with parts licensed under bzip2 and Common Public License version 1.0. The installer automates installation process and is applicable in Windows only. Apache Derby database (Apache License, Version 2.0) is required to compile HTDP (http://db.apache.org/derby/). The program source code, binary installation files and documentation have been published as an open source project at GitHub (https://github.com/pmadanecki/htdp) and Sourceforge (https://sourceforge.net/projects/htdp/) under a GNU GPL license version 3.0, in addition to a website that provides supporting information (http://en.biology.pl/downloads, https://osf.io/pw2dx/). The program has been tested extensively using the MS Windows 7 and Linux (Debian 7 and 8) operating systems and has been confirmed to run on the Mac OS X (with JRE 1.6 installed).

All supporting files were deposited on Open Science Framework (https://osf.io/pw2dx/) and are publicly available.

## Supporting information

S1 File High-Throughput Tabular Data Processor user manual. (PDF)

S2 File Examples description. (PDF)
