# Supplementary material for: High-Throughput Tabular Data Processor – Platform independent graphical tool for processing large data sets
Source: PLoS One. 2018 Feb 12;13(2):e0192858. doi: 10.1371/journal.pone.0192858 (PMC5809091; doi:10.1371/journal.pone.0192858)

S1 File. User Manual

1. INTRODUCTION 3
2. INSTALLATION AND RUNNING 4
2.1 System requirements: 4
2.2 Windows (automated installation): 4
2.3 Windows (manual installation): 4
2.4 Linux: 4
3. INPUT FILE FORMAT AND LIMITS: 5
4. THE MAIN WINDOW OF THE PROGRAM – BASIC MODE 7
5. THE MAIN WINDOW OF THE PROGRAM – ADVANCED MODE 8
6. QUERY CONFIGURATION WINDOW – ADVANCED MODE 10
7. BASIC MODE – USAGE 12
7.1 FILE IMPORT 12
7.2 MAIN VIEW 17
7.2.1 PULL DOWN MENU 17
7.2.2 REPORT PREVIEW 21
7.2.3 INFORMATION AND STATUS BAR 22
7.2.4 THE CONTROL BUTTONS 22
7.3 22
7.4 COLUMN SORTING 22
7.5 COLUMN MANAGEMENT 24
7.5.1 MANAGE DATA COLUMNS field 25
7.5.1.1 COLUMN PROPERTIES subfield 26
7.5.1.2 COLUMN ORDERING subfield 28
7.5.2 FILE SELECTION field 29
7.6 FILTERING THE INPUT FILE(S) 30
7.7 SAVING FILTERED FILE(S) 34
7.8 SAVING QUERIES 35
7.9 LOADING QUERIES 36
8. ADVANCED MODE – USAGE 37
8.1 FILE IMPORT 37
8.2 MAIN VIEW 41
8.2.1 PULL DOWN MENU 41
8.2.2 MAIN INPUT FILE / DIRECTORY field 45
8.2.3 SECOND, OPTIONAL FILE / DIRECTORY field 45
8.2.4 FILE LIST field 46
8.2.5 QUEUE field 47
8.2.5.1 QUEUE subfield 47
8.2.5.2 QUERY QUEUE CONTROL SECTION 47
8.2.6 REPORT PREVIEW 49
8.2.7 INFORMATION AND STATUS BAR 49
8.2.8 FILE OPERATIONS BUTTONS 50
8.3 SORTING OF COLUMNS 51
8.3.1 MANAGE DATA COLUMNS field 53
8.3.1.1 COLUMN PROPERTIES subfield 54
8.3.1.2 COLUMN ORDERING subfield 56
8.3.2 FILE SELECTION field 57

8.4 FILTERING THE INPUT FILE(S) 58
8.4.1 DATA PREPROCESSING 60
8.4.1.1 COLUMN SELECTION field 63
8.4.1.2 INPUT DATA PREPROCESSING field 64
8.4.1.3 QUERY SETUP field 66
8.4.2 LOCATION FILTER 69
8.4.2.1 SELECT COLUMN OR CREATE A LOCUS field 69
8.4.2.2 LOCUS SEARCH field 71
8.4.2.3 SELECT COLUMNS AND CONDITION field 73
8.4.2.4 SELECT ACTION field 76
8.4.2.5 ADDITIONAL COLUMNS AND STATISTICS subfield 77
8.4.2.6 SELECT COLUMN OR CREATE LOCUS field 79
8.4.2.7 SELECT COLUMN OR CREATE LOCUS (EXTERNAL DATA) field ... 81
8.4.2.8 CONDITION field 81
8.4.2.9 SELECT ACTION field 82
8.4.2.10 ADDITIONAL COLUMNS AND STATISTICS subfield 84
8.5 GENERATING REPORT 87
8.6 CANCELLING OF INTRODUCED CHANGES 88
8.7 SAVING FILTERED FILE(S) 89
8.8 EXPORTING FILTERED FILE(S) 90
8.9 SAVING QUERIES 91
8.10 LOADING QUERIES 92

1. INTRODUCTION

HIGH-THROUGHPUT TABULAR DATA PROCESSOR (HTDP) is Java application that is intended to facilitate data exploration and reduction tasks in large text files resulting from high throughput technologies, e.g. massively parallel sequencing or microarrays. The software has been optimized for microarray and deep parallel sequencing data, however it can accept any character delimited tabular data sets. In the latter case the first row of the data set should be designated as header row and should contain the names of the corresponding columns. HTDP can also import, process and convert Variant Call Format (VCF) files ver. 4.0, 4.1 and 4.2 (http://www.1000genomes.org/wiki/Analysis/Variant%20 Call%20Format/ vcf-variant-call-format-version-41). HTDP provides quick filtering functionality and can process data consisting of single or multiple input files. Files in different supported formats can be processed at the same time. The processed data can be exported as tab delimited file. The user interface provides two modes of operation: “basic mode” (default) and “advanced mode”.

Data filtering in the basic mode is carried out via selection of search terms from the pull down list or manual input of alphanumeric strings into a simple form (up to 8 queries). Data filtering in the advanced mode is carried out based on user defined unlimited queries. Subsequent queries are added to the queue, which constitutes a set of filtering criteria. This set of filtering criteria is applied to every row in the input file(s).

HTDP is distributed under GPL License version 3.0. Nullsoft Scriptable Install System (NSIS, https://sourceforge.net/projects/nsis/) was used to generate windows installer. NSIS is licensed as zlib/libpng licensed software with parts licensed under bzip2 and Common Public License version 1.0. The installer automates installation process and is applicable in Windows only. Apache Derby database (Apache License, Version 2.0) is required to compile HTDP (http://db.apache.org/derby/).

2. INSTALLATION AND RUNNING

2.1 System requirements:

- Windows XP, Windows 7 or Linux with Java run time environment v. 1.6 or later (64-bit version)*.
- At least 1 GB of RAM, depending on the amount of input data and complexity of queries.

*Default Java version is 32-bit and its maximum theoretical heap limit is 4GB. To increase the amount of allocated memory and therefore the performance of this program, the 64-bit version should be used.

2.2 Windows (automated installation):

- Run the installation program (htdpinstaller.exe) and follow the on screen tips.
- The installation program will create program group "**HIGH-THROUGHPUT TABULAR DATA PROCESSOR**" in Windows Start Menu.
- The program group contains the shortcut to the "**HIGH-THROUGHPUT TABULAR DATA PROCESSOR**" as well as to the uninstall program.
- Additional shortcut to the "HIGH-THROUGHPUT TABULAR DATA PROCESSOR" will be created on Windows desktop.

2.3 Windows (manual installation):

Copy **HTDP.jar file** and **lib folder** from the installation archive to selected folder on local hard drive. Run the program using the following command:

"java -Xmx800M -jar "/path/to/the/program/folder/HTDP.jar"

2.4 Linux:

Copy **HTDP.jar** file from the installation archive to selected folder on local hard drive. Run the program using the following command:

"java -Xmx800M -jar "/path/to/the/program/folder/HTDP.jar"

3. INPUT FILE FORMAT AND LIMITS:

- The following file formats can be imported and processed:
- **Any Character-Delimited Tabular Text File** with optional single line header row. File has to contain at least four rows of data to be correctly recognized and opened. This includes **Variant Call Format (VCF)** ver. 4.0, 4.1 and 4.2 (http://www.1000genomes.org/wiki/Analysis/Variant%20Call%20Format/vcf-variant-call-format-version-41) and other uncompressed text data formats like BED, BED detail, PSL, GFF, Personal Genome SNP, ENCODE RNA elements: BED6 + 3 scores, ENCODE narrowPeak: Narrow (or Point-Source) Peaks, ENCODE broadPeak: Broad Peaks (or Regions), ENCODE gappedPeak: Gapped Peaks (or Regions), ENCODE peptideMapping: BED6+4, detailed formats description: https://genome.ucsc.edu/FAQ/FAQformat.html.
- Any user defined delimiting character is allowed for input files. The most frequently used delimiters are predefined and selectable from the list (comma, semicolon, dot). Any other character that is used as delimiter may be specified by the user. The preview of opened file was implemented with automatic refresh when other delimitation character is selected/entered.
- Comment character is user defined; hence any arbitrarily chosen character can be used as comment delimiter.
- If there are no column names in the input file, the column names will be created automatically upon file opening. File preview function allows specifying whether a file has column names and what data delimiters or comment characters are used. Additionally, while opening multiple files (directory) different settings can be applied separately to each file.
- Similarly, rows are not required to have row names. The unique row numbers and other information (e.g. file name) is added automatically (as system data).
- Missing data values are allowed in the input files. The format for missing data values is flexible. This can be either empty space or “NA” or “null”. The main requirement is that the missing data values should be denoted in the same way in the whole data set. It is also possible to replace the missing data values with a different symbol after the file has been opened.
- The maximum values for data rows and data columns are essentially limited by the available operating system memory (RAM). Albeit, there is intrinsic limit in Java language specification (theoretical, maximum number of elements indexed in an array should not exceed $2^{31}-1$, i.e. 2 147 483 647), but in most real life situations limitations related to available RAM will occur first.
- There is no limit of number of files that HTDP can work with at the same time. The only limits here are related to intrinsic features of Java or available RAM. Sample memory requirement and performance test results are presented in Table 1 below.

Table 1. Sample memory requirement and performance test results.

Allocated memory (max. RAM usage allowed)	Max input file size (format)	Rows	Additional information
4 GB	228,4 MB (VCF)[a]	1 750 000	Opening time: about 6 min. Simple query (get unique rows only on the basis of selected column contents): 8 sec.
8 GB	457,9 MB (VCF) [a]	3 500 000	Opening time: about 9 min. Simple query (get unique rows only on the basis of selected column content): 21 sec.
16 GB	918,7 MB (VCF) [a]	7 000 000	Opening time: about 17 min. Simple query (get unique rows only on the basis of selected column content):46 sec.
4GB	184,8 MB (TDTF) [a]	4 800 000	Opening time: about 2 min. Simple query (get unique rows only on the basis of selected column content): 48 sec.
8 GB	388,4 MB (TDTF) [a]	9 700 000	Opening time: about 4 min. Simple query (get unique rows only on the basis of selected column content): 95 sec.
16 GB	704,9 MB (TDTF) [a]	17 500 000	Opening time: about 6 min. Simple query (get unique rows only on the basis of selected column content): 2 min.

Methodology: Linux (Debian 8) ‘split’ command was used to split large files into the desired number of rows as indicated in the table. Java ‘Xmx’ parameter was used to set java virtual machine accessible memory limit. All tests were carried out on a workstation equipped with Intel Xeon E5-1650v3 3,5GHz processor.

[a] TDTF and VCF files are both character-delimited files. However, VCF files often have richer annotation compared to simple tab-delimited files, as well as VCF files utilize multiple delimiters (tabs, colons and semicolons) in a single file. Hence, VCF and TDTF files were subjected to the test separately to illustrate differences in the initial processing time during file opening. The tab delimited text file (TDTF) (6 columns) and VCF file (32 columns) were used.

4. THE MAIN WINDOW OF THE PROGRAM – BASIC MODE

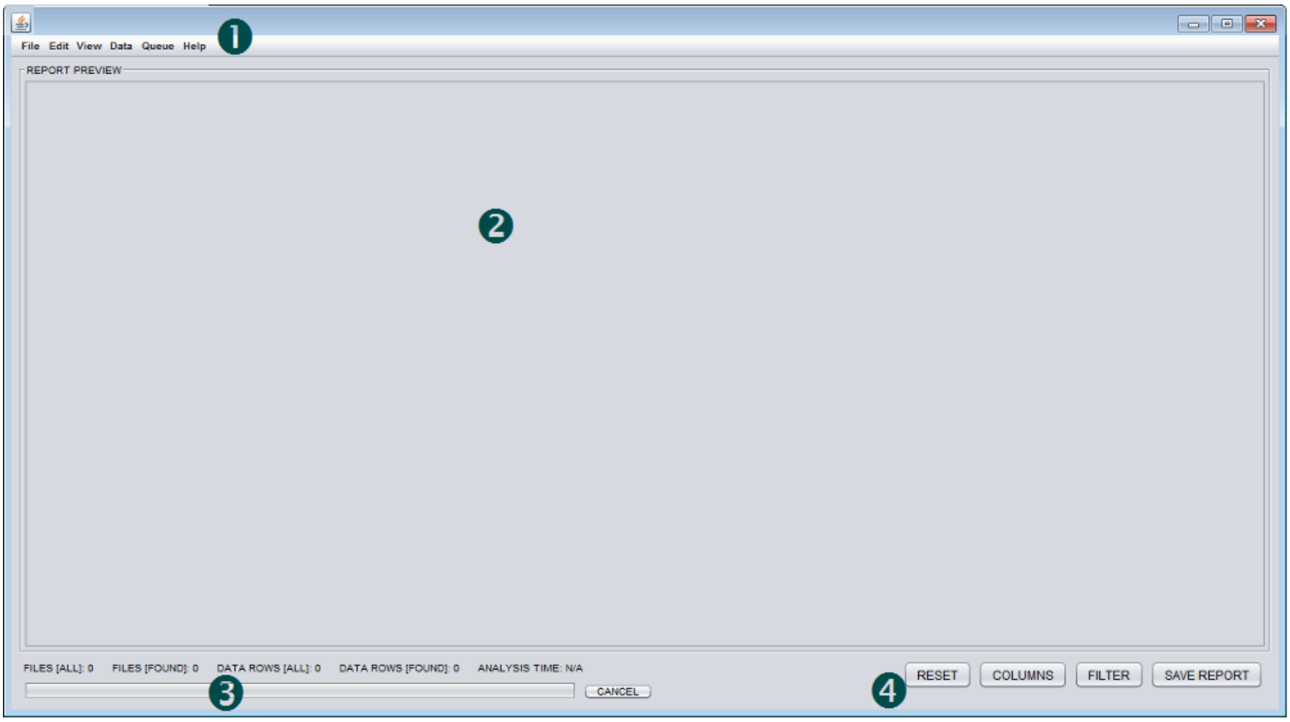

❶– Pull down text menu with program options

❷– Preview of the data, before and after filtering (**REPORT PREVIEW**)

❸– Information and status bar

❹– The buttons for column and file operations (**RESET**, **COLUMN**, **FILTER**, **SAVE REPORT**)

5. THE MAIN WINDOW OF THE PROGRAM – ADVANCED MODE

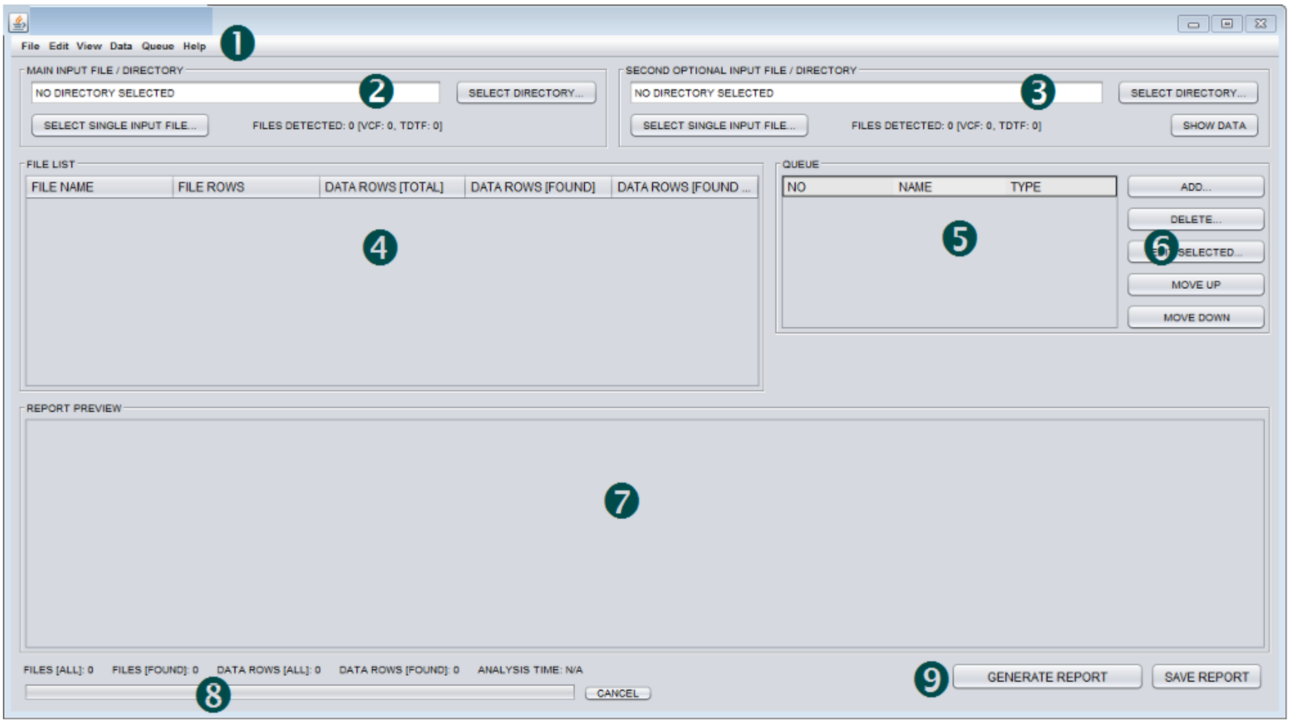

1 – Pull down text menu with program options

2 – Input data folder selection form (**MAIN INPUT FILE / DIRECTORY**)

3 – Selection form for the second file or folder (**SECOND, OPTIONAL INPUT FILE / DIRECTORY**)

4 – List of imported files (**FILE LIST**)

5 – The list of user specified queries (**QUEUE**)

6 – Query queue control section

7 – Preview of the resulting data table (**REPORT PREVIEW**)

❽– Information and status bar

❾– The buttons for file operations (**GENERATE REPORT**, **SAVE REPORT**)

6. QUERY CONFIGURATION WINDOW – ADVANCED MODE

The **QUERY SETUP** window is available only in the advanced mode. It enables forming subsequent, complex queries.

To set up a query, click the **ADD** button in the query queue control section (❷). Subsequently added queries will show up as a list in the **QUEUE** field (❶).

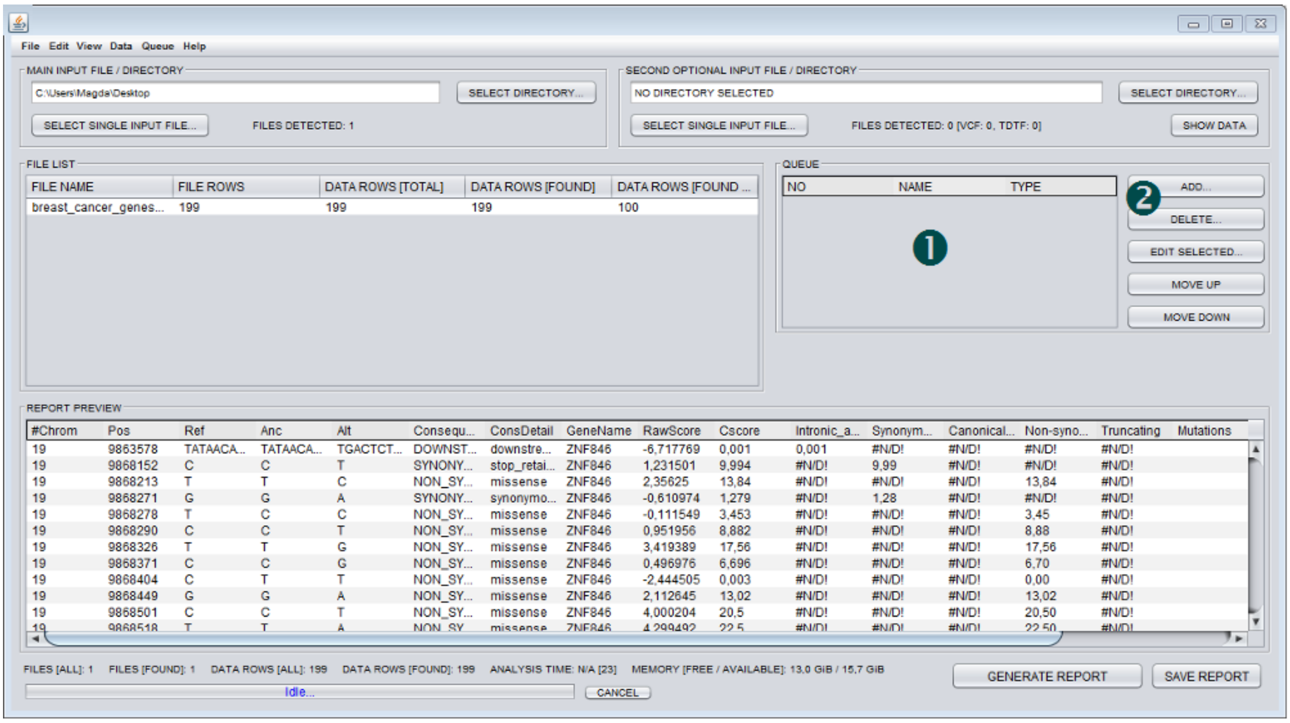

In the present version of the program the **QUERY SETUP** window has six tabs corresponding to the different types of queries that can be handled by the program (see 7.6 FILTERING THE INPUT FILE(S) section):

- **DATA PREPROCESSING**.
- **SIMPLE FILTER**.
- **LOCATION FILTER**.
- **EXTERNAL DATA FILTER / MERGER**.
- **EXTERNAL DATA FILTER / MERGER (LOCATION)**.
- **SAMPLE FILTER**.

DATA PREPROCESSING tab is selected by default.

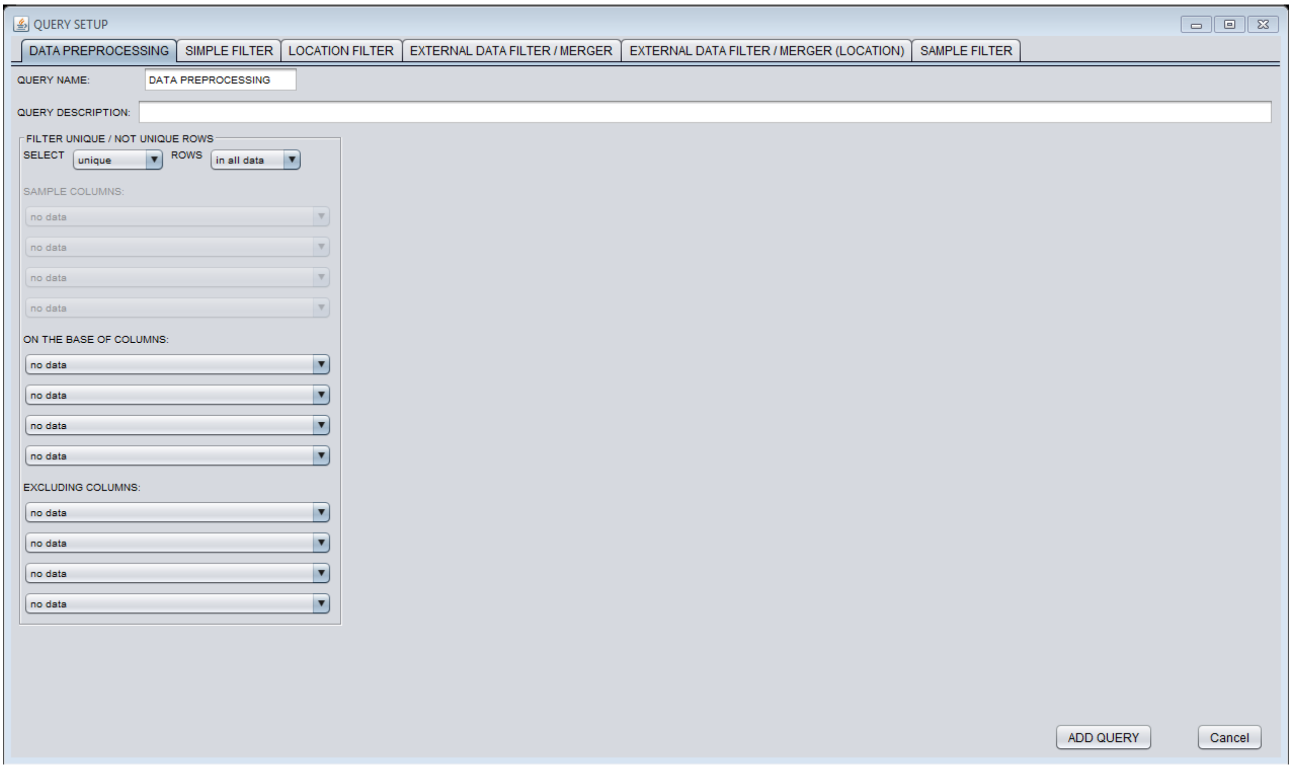

7. BASIC MODE – USAGE

The **BASIC MODE** is intended for simple tasks, specifically for viewing the combined data from multiple files and simplified alphanumeric filtering which may however consist of several steps linked by Boolean operators.

7.1 FILE IMPORT

To import file(s) which will be subjected to filtering:

1 Copy the input files to a single directory. Select **File > Open directory** menu option and point to this directory in **Select directory** window. The supported data formats are automatically detected and listed upon data import.

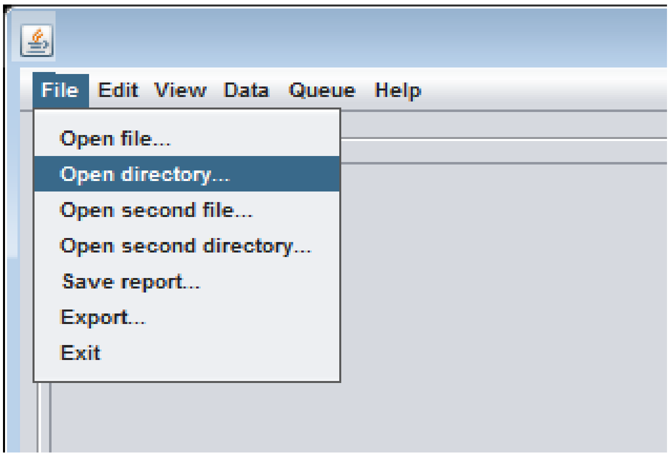

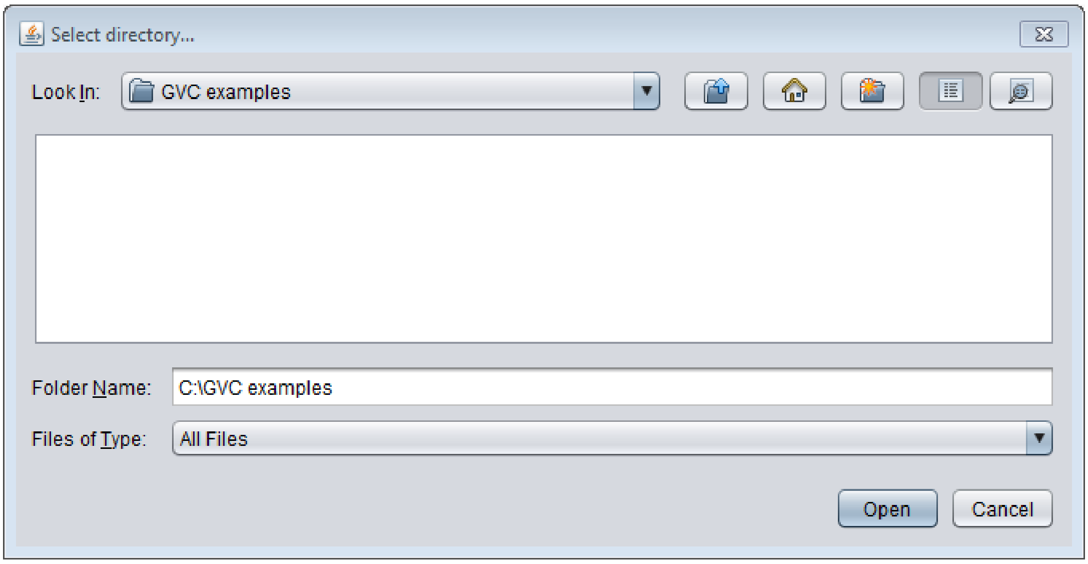

Alternatively, a single file can be imported. To accomplish it, select **File > Open file** menu option and point the file in **Select single file** window.

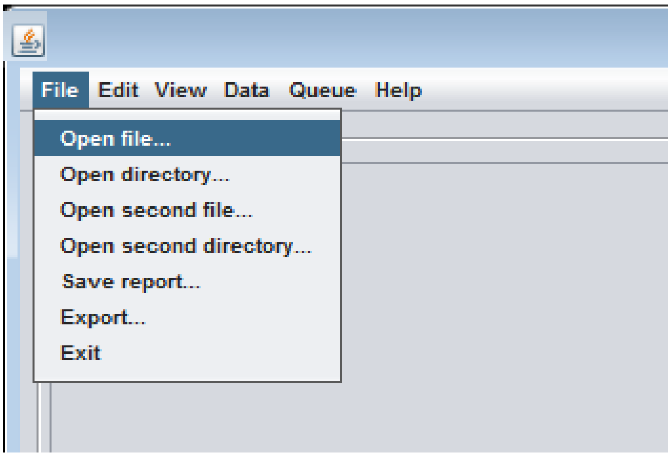

2 The status bar displays file import progress information in real time. The import time depends on the total size of the import files and available computing power and RAM.

3 In the **SELECT FILES** window mark the files from the import directory to open. Using buttons in **SELECT FILES TO OPEN** subfield it is possible to simultaneously select or deselect for opening all detected files (**MARK ALL TO OPEN** / **MARK ALL NOT TO OPEN**) or to invert the selection of files (**INVERT SELECTION**). Column delimiter and comment marker may be set independently for each opened file with data preview. The predefined values may be selected using the menu or simply typed in menu field and confirmed by pressing enter key. Selected settings may be applied to all opened files (by pressing **APPLY TO ALL FILES** button) or set to each file separately.

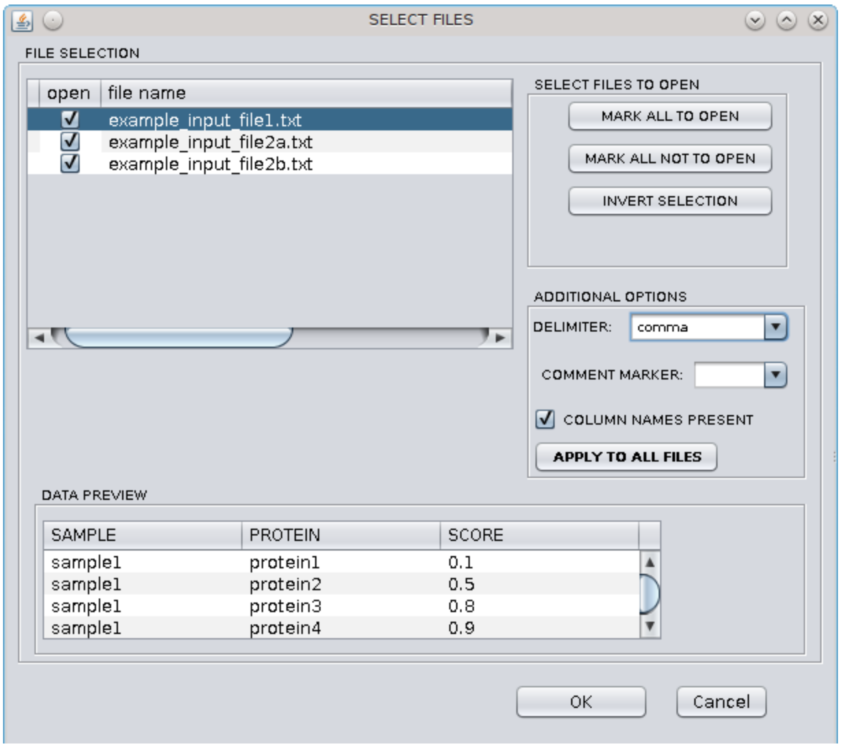

In the **SELECT COLUMNS** window select the data columns to be imported from the input file(s) using the same buttons as in the previous window (**MARK ALL TO OPEN, MARK ALL NOT TO OPEN** and **INVERT SELECTION**). Optionally, reorganize the data columns with **MOVE UP** and **MOVE DOWN** buttons in **COLUMN ORDERING** subfield.

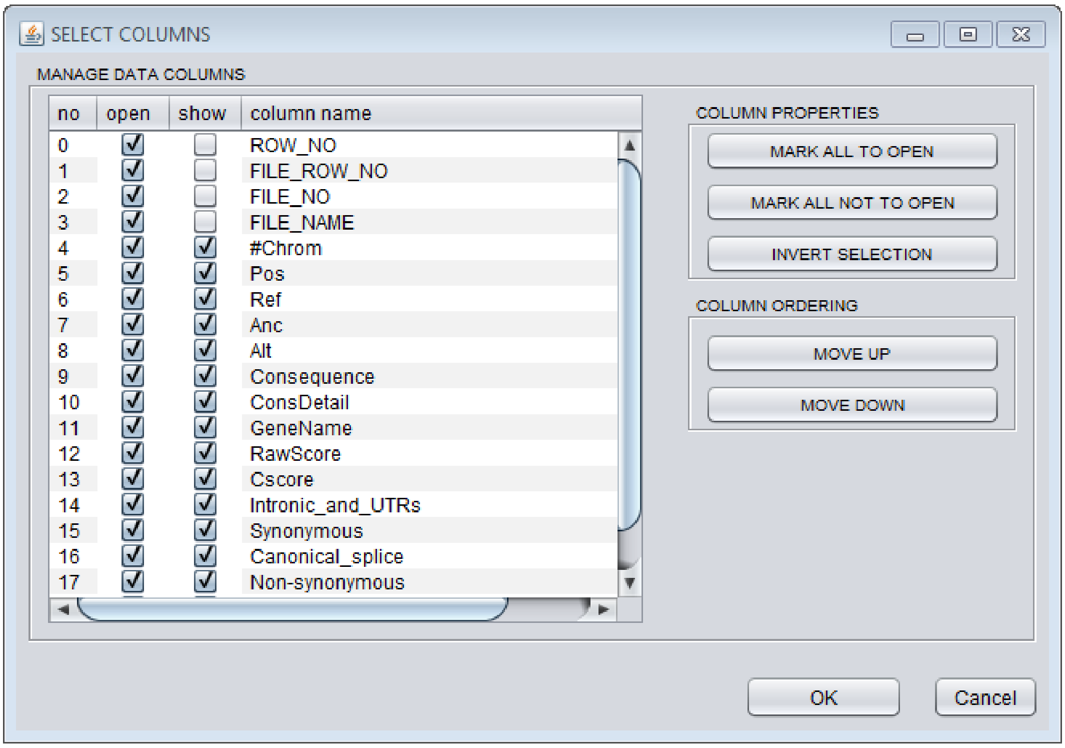
SELECT COLUMNS
MANAGE DATA COLUMNS
no
open
show
column name
0 ROW_NO
1 FILE_ROW_NO
2 FILE_NO
3 FILE_NAME
4 #Chrom
5 Pos
6 Ref
7 Anc
8 Alt
9 Consequence
10 ConsDetail
11 GeneName
12 RawScore
13 Cscore
14 Intronic_and_UTRs
15 Synonymous
16 Canonical_splice
17 Non-synonymous
COLUMN PROPERTIES
MARK ALL TO OPEN
MARK ALL NOT TO OPEN
INVERT SELECTION
COLUMN ORDERING
MOVE UP
MOVE DOWN
OK
Cancel

4 When the file import has been completed, the **REPORT PREVIEW** is generated, which contains the combined data from the imported files.

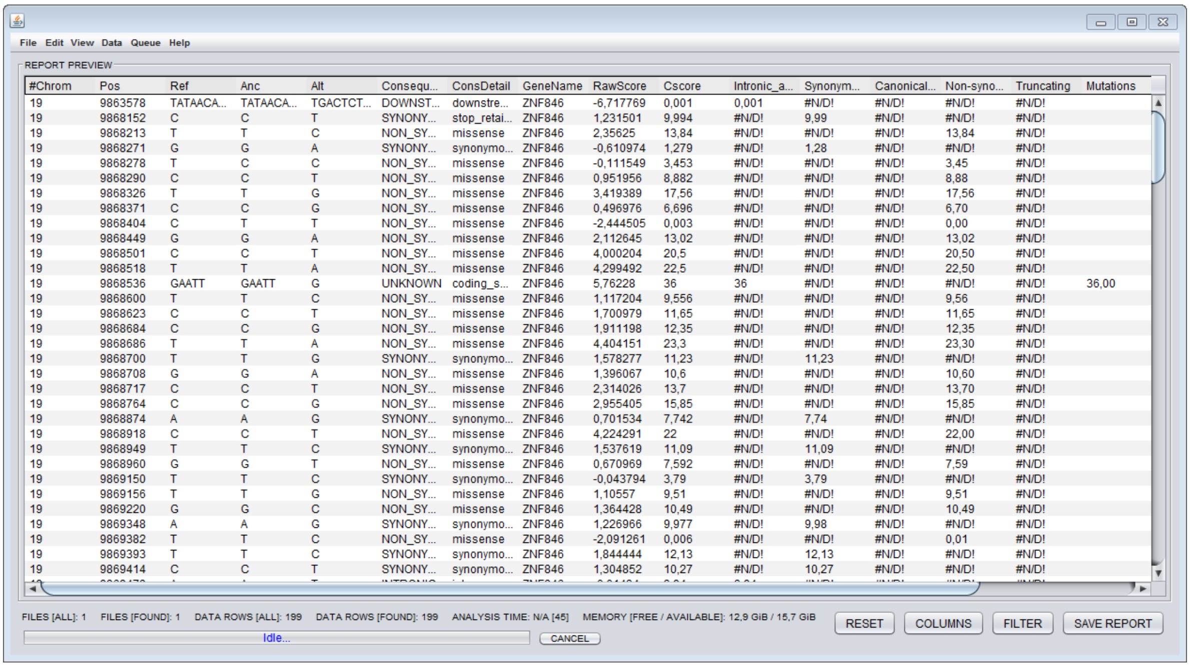

#Chrom	Pos	Ref	Anc	Alt	Consequ...	ConsDetail	GeneName	RawScore	Cscore	Intronic_a...	Synonym...	Canonical...	Non-syno...	Truncating	Mutations
19	9863578	TATAACA...	TATAACA...	TGACTCT...	DOWNST...	downstre...	ZNF846	-6,717769	0,001	0,001	#N/D!	#N/D!	#N/D!	#N/D!	
19	9868152	C	C	T	SYNONY...	stop_retai...	ZNF846	1,231501	9,994	#N/D!	9,99	#N/D!	#N/D!	#N/D!	
19	9868213	T	T	C	NON_SY...	missense	ZNF846	2,35625	13,84	#N/D!	#N/D!	#N/D!	13,84	#N/D!	
19	9868271	G	G	A	SYNONY...	synonymo...	ZNF846	-0,610974	1,279	#N/D!	1,28	#N/D!	#N/D!	#N/D!	
19	9868278	T	C	C	NON_SY...	missense	ZNF846	-0,111549	3,453	#N/D!	#N/D!	#N/D!	3,45	#N/D!	
19	9868290	C	C	T	NON_SY...	missense	ZNF846	0,951956	8,882	#N/D!	#N/D!	#N/D!	8,88	#N/D!	
19	9868326	T	T	G	NON_SY...	missense	ZNF846	3,419389	17,56	#N/D!	#N/D!	#N/D!	17,56	#N/D!	
19	9868371	C	C	G	NON_SY...	missense	ZNF846	0,496976	6,696	#N/D!	#N/D!	#N/D!	6,70	#N/D!	
19	9868404	C	T	T	NON_SY...	missense	ZNF846	-2,444505	0,003	#N/D!	#N/D!	#N/D!	0,00	#N/D!	
19	9868449	G	G	A	NON_SY...	missense	ZNF846	2,112645	13,02	#N/D!	#N/D!	#N/D!	13,02	#N/D!	
19	9868501	C	C	T	NON_SY...	missense	ZNF846	4,000204	20,5	#N/D!	#N/D!	#N/D!	20,50	#N/D!	
19	9868518	T	T	A	NON_SY...	missense	ZNF846	4,299492	22,5	#N/D!	#N/D!	#N/D!	22,50	#N/D!	
19	9868536	GAATT	GAATT	G	UNKNOWN	coding_s...	ZNF846	5,76228	36	36	#N/D!	#N/D!	#N/D!	#N/D!	36,00
19	9868600	T	T	C	NON_SY...	missense	ZNF846	1,117204	9,556	#N/D!	#N/D!	#N/D!	9,56	#N/D!	
19	9868623	C	C	T	NON_SY...	missense	ZNF846	1,700979	11,65	#N/D!	#N/D!	#N/D!	11,65	#N/D!	
19	9868684	C	C	G	NON_SY...	missense	ZNF846	1,911198	12,35	#N/D!	#N/D!	#N/D!	12,35	#N/D!	
19	9868686	T	T	A	NON_SY...	missense	ZNF846	4,404151	23,3	#N/D!	#N/D!	#N/D!	23,30	#N/D!	
19	9868700	T	T	G	SYNONY...	synonymo...	ZNF846	1,578277	11,23	#N/D!	11,23	#N/D!	#N/D!	#N/D!	
19	9868708	G	G	A	NON_SY...	missense	ZNF846	1,396067	10,6	#N/D!	#N/D!	#N/D!	10,60	#N/D!	
19	9868717	C	C	T	NON_SY...	missense	ZNF846	2,314026	13,7	#N/D!	#N/D!	#N/D!	13,70	#N/D!	
19	9868764	C	C	G	NON_SY...	missense	ZNF846	2,955405	15,85	#N/D!	#N/D!	#N/D!	15,85	#N/D!	
19	9868874	A	A	G	SYNONY...	synonymo...	ZNF846	0,701534	7,742	#N/D!	7,74	#N/D!	#N/D!	#N/D!	
19	9868918	C	C	T	NON_SY...	missense	ZNF846	4,224291	22	#N/D!	#N/D!	#N/D!	22,00	#N/D!	
19	9868949	T	T	C	SYNONY...	synonymo...	ZNF846	1,537619	11,09	#N/D!	11,09	#N/D!	#N/D!	#N/D!	
19	9868960	G	G	T	NON_SY...	missense	ZNF846	0,670969	7,592	#N/D!	#N/D!	#N/D!	7,59	#N/D!	
19	9869150	T	T	C	SYNONY...	synonymo...	ZNF846	-0,043794	3,79	#N/D!	3,79	#N/D!	#N/D!	#N/D!	
19	9869156	T	T	G	NON_SY...	missense	ZNF846	1,10557	9,51	#N/D!	#N/D!	#N/D!	9,51	#N/D!	
19	9869220	G	G	C	NON_SY...	missense	ZNF846	1,364428	10,49	#N/D!	#N/D!	#N/D!	10,49	#N/D!	
19	9869348	A	A	G	SYNONY...	synonymo...	ZNF846	1,226966	9,977	#N/D!	9,98	#N/D!	#N/D!	#N/D!	
19	9869382	T	T	C	NON_SY...	missense	ZNF846	-2,091261	0,006	#N/D!	#N/D!	#N/D!	0,01	#N/D!	
19	9869393	T	T	C	SYNONY...	synonymo...	ZNF846	1,844444	12,13	#N/D!	12,13	#N/D!	#N/D!	#N/D!	
19	9869414	C	C	T	SYNONY...	synonymo...	ZNF846	1,304852	10,27	#N/D!	10,27	#N/D!	#N/D!	#N/D!	

7.2 MAIN VIEW

7.2.1 PULL DOWN MENU

At the top of the main window there is a pull down menu with six program menus, namely **File**, **Edit**, **View**, **Data**, **Queue** and **Help**.

File menu refers to following operations on files – opening a single file, opening a directory with files, opening second file or directory, saving report with processed data and exiting the program.

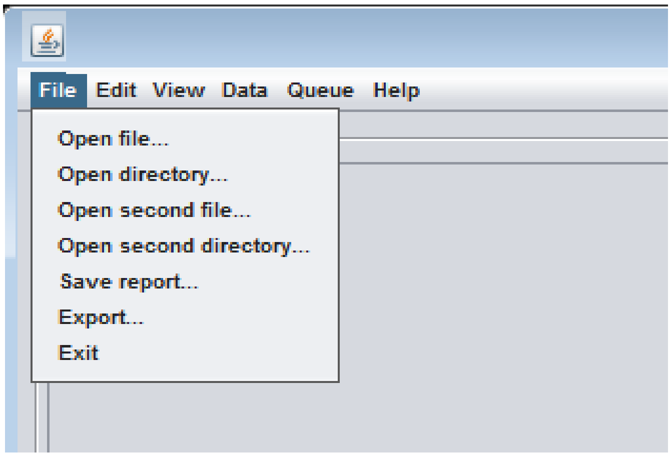

Making use of **Edit** menu, the global data changes can be done – such as carrying out the filtering task and generating a resulting report or resetting all settings. Clearing all actually loaded data is also possible.

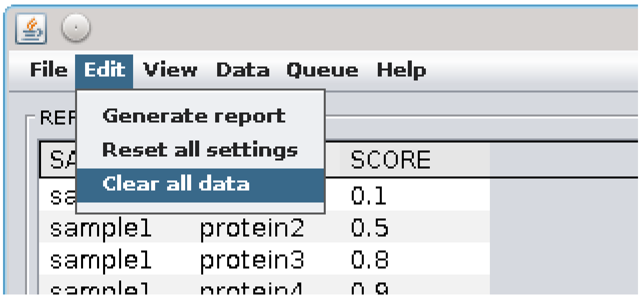

Switching between the basic and advanced mode is available via **View** menu**.**

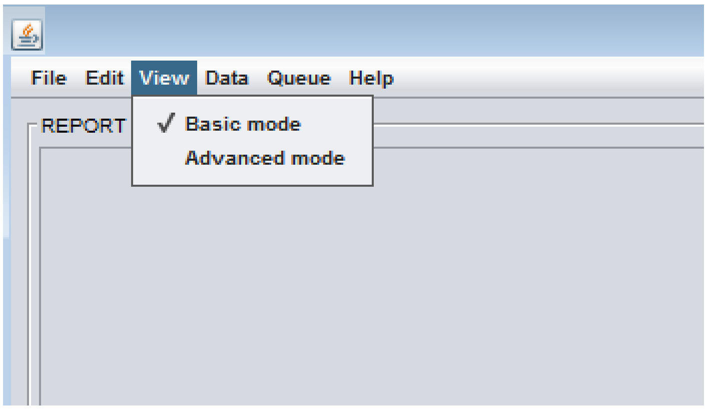

Functions designed for data processing are provided in **Data** menu. Functions called **Column manager**, **Sort** and **Filter** are also available via buttons in the bottom right of the main window (see **Error! Reference source not found.** COLUMN MANAGEMENT, **Error! Reference source not found.** COLUMN SORTING or 7.6 FILTERING THE INPUT FILE(S) section, respectively).

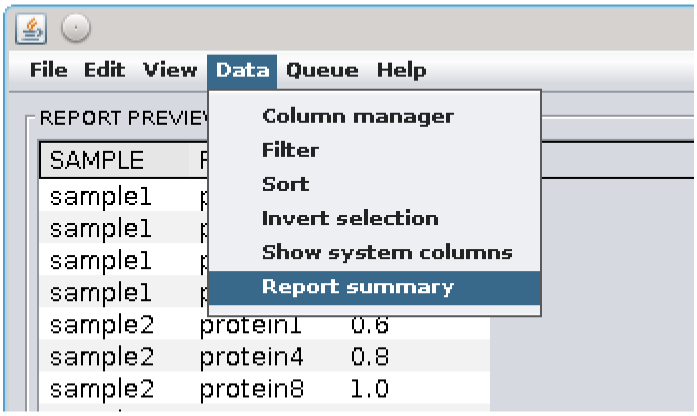

Under **Data** menu it is also possible to reverse the output with regards to the applied filtering criteria using **Invert selection** function. The invert selection function is intended for control purposes, i.e. to provide an insight into data that is removed by the filters.

Show system columns function is intended to determine whether system columns are displayed or hidden. System columns, called **ROWS_NO**, **FILE_ROW_NO**, **FILE_NO** and **FILE_NAME**, are created automatically by the program to keep track of the original records in the imported files. **Show system columns** function is mainly helpful when working with multiple files at the same time.

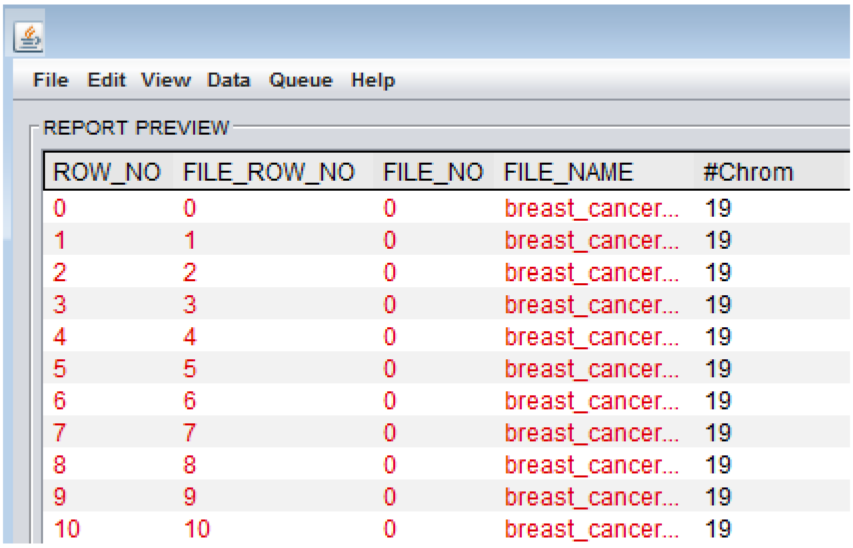

Report summary gives some useful information about the most recent task run time, system memory and processed files (number of rows/number of selected rows). These data can be exported to tab delimited text file for additional use. Additionally, there are graphical bars where these simple information is graphically presented (total number of files, data rows, system memory are in green; actual selected files, deata rows, and memory used in blue and maximum java memory limit in red).

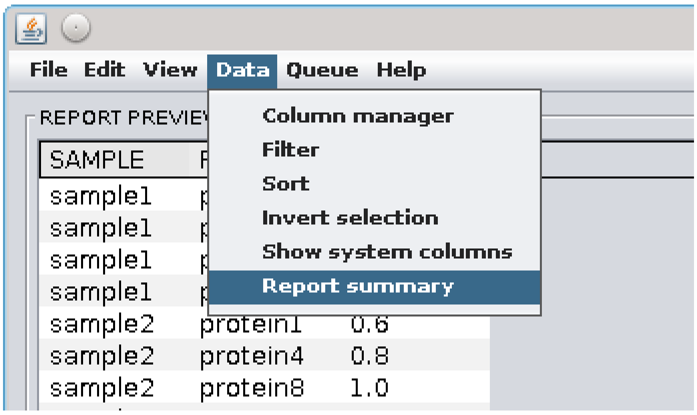

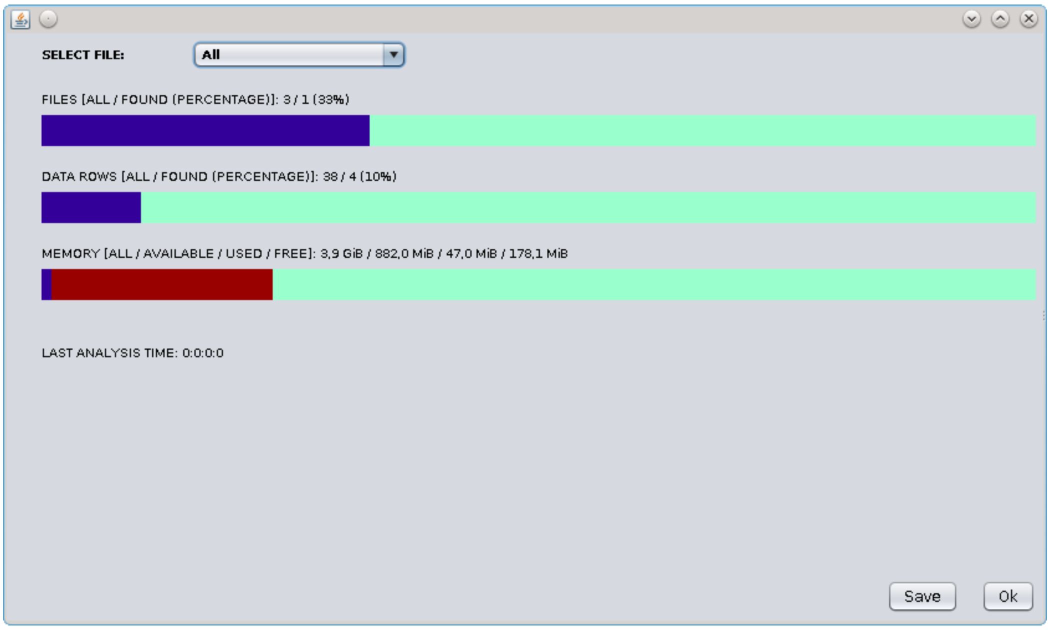

Add query function under **Queue** menu facilitates adding a query with regard to imported data.It is also possible to save a query consisting of particular parameters for further analyses and to load the previously saved query if needed (see **Error! Reference source not found.** SAVING QUERIES and 7.9 LOADING QUERIES sections).

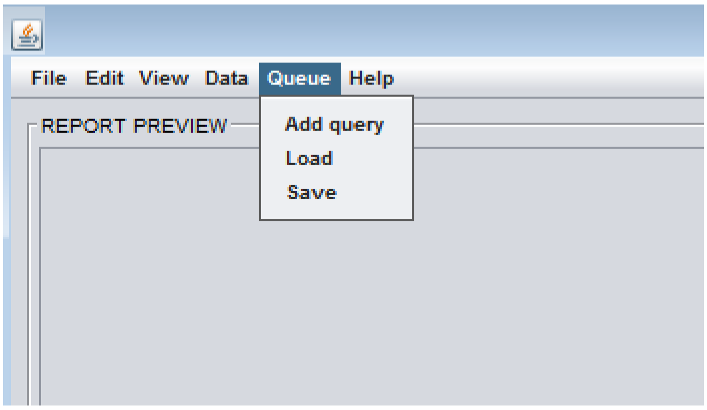

Information about the **HIGH-THROUGHPUT TABULAR DATA PROCESSOR** and this **user manual** is accessible via **Help** menu.

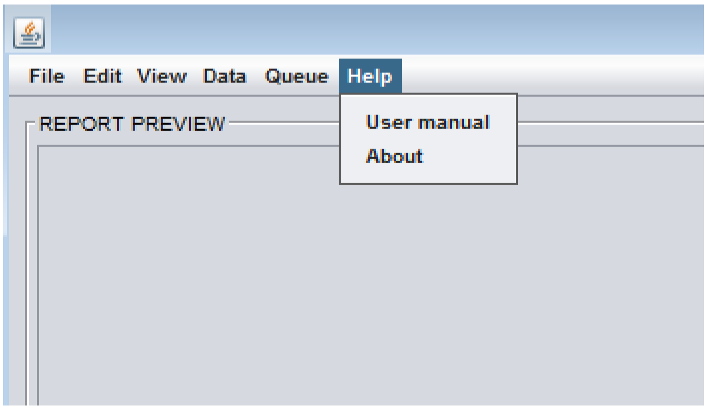

7.2.2 REPORT PREVIEW

In the basic mode the main portion of the application window is occupied by the **REPORT PREVIEW** table.

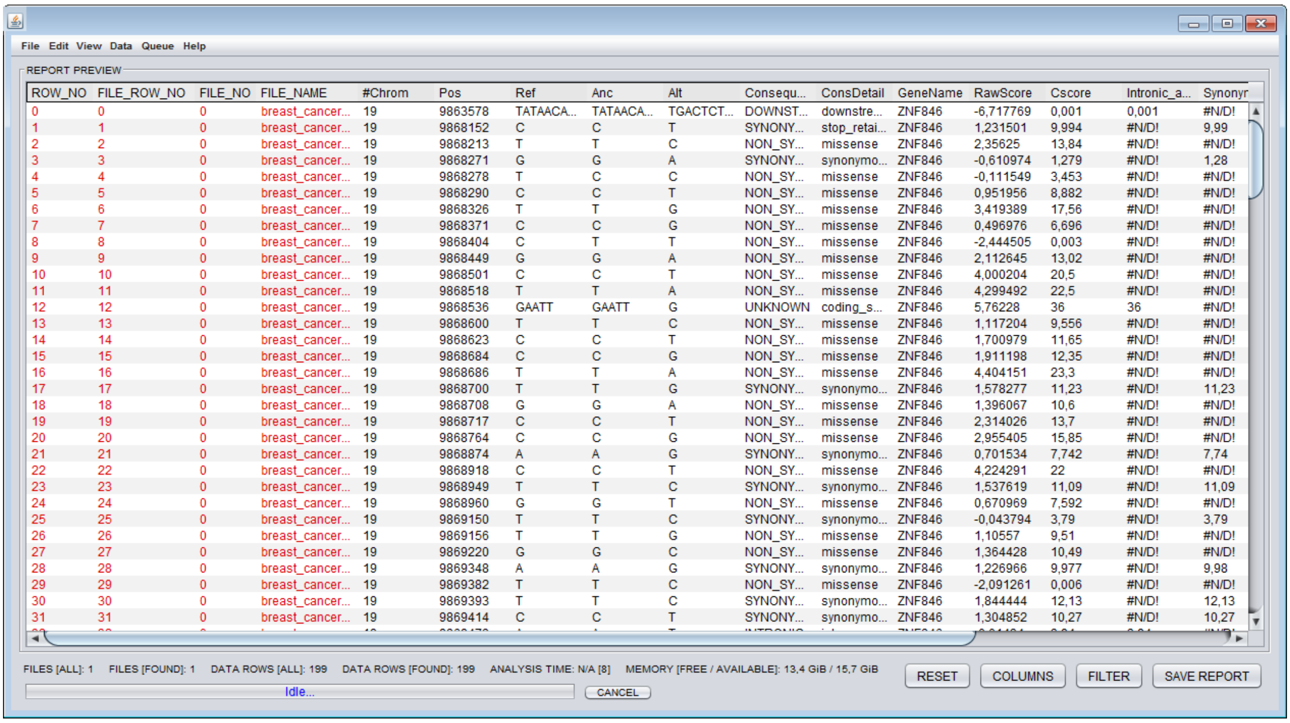

Table is updated in real time along with execution of the filtering (see 7.6 FILTERING FILES section). The table can be sorted by any column after clicking the column name or

selecting **Data > Sort** menu option (see **Error! Reference source not found.** COLUMN SORTING section).
This table is also editable – double click on certain field, type some text and the modified content will be saved.

7.2.3 INFORMATION AND STATUS BAR

The information bar, located at the bottom of the main window, shows aggregate data statistics:

- Number of imported files (**FILES [ALL]**).
- Number of imported files which have retained at least one row of data after the filtering has been applied (**FILES [FOUND]**).
- The total number of rows in all imported files prior to filtering (**DATA ROWS [ALL]**).
- The total number of rows in all imported files which are retained after the filtering criteria have been applied (**DATA ROWS [FOUND]**).
- Total analysis time after completion of all items in the query queue (**ANALYSIS TIME**).

Status bar is located below aggregate data statistics and shows progress of the analysis in real time.

The **CANCEL** button allows to abort an ongoing operation.

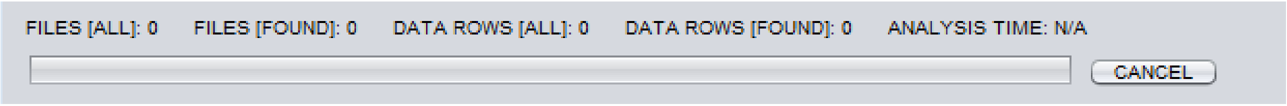

7.2.4 THE CONTROL BUTTONS

These four buttons, namely **RESET**, **COLUMN**, **FILTER** and **SAVE REPORT**, are located in the bottom right corner of the main window. They are created to facilitate operations on columns and files. The specific functions are described in the following sections: 7.6. CANCELLING OF CHANGES, 7.4. COLUMN MANAGEMENT, 7.5. FILTERING THE INPUT FILE(S), 7.7. SAVING FILTERED FILE(S), respectively.

7.3

7.4 COLUMN SORTING

To rearrange the data set with respect to one or more columns select **Data > Sort** in pull down menu or click on any column name in the header line in the **REPORT PREVIEW**

field. The **DATA SORT** window which is opened facilitates sorting by up to the three columns at a time, in ascending or descending manner and in case insensitive (by default) or sensitive way.

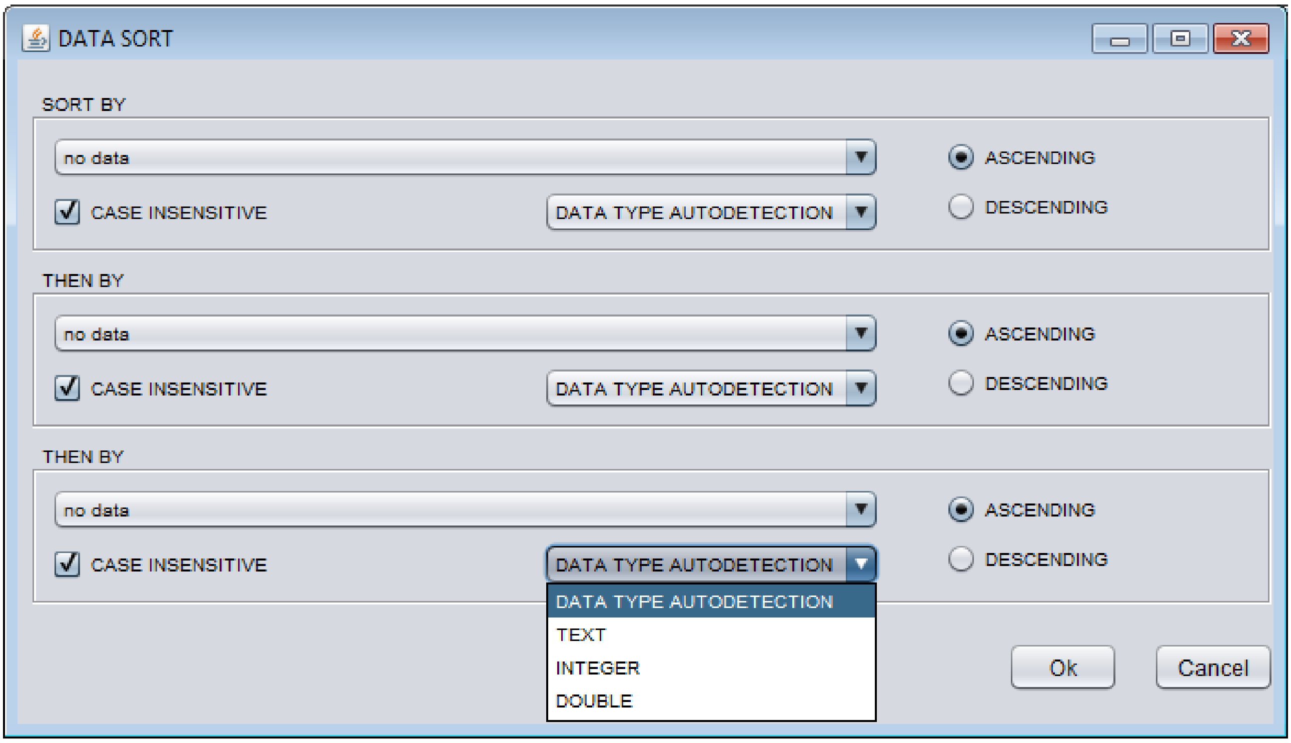

The type of data in the column(s) subjected to sorting is detected automatically (**DATA TYPE AUTODETECTION**), but can be also selected by hand as **TEXT**, **INTEGER** or **DOUBLE** (double-precision floating-point numbers, namely decimals).

7.5 COLUMN MANAGEMENT

Column(s) and file(s) management is possible via the **COLUMN MANAGER** window. To open it, select **Data** > **Column manager** in pull down menu or click on **COLUMNS** button in the main screen.

This window consists of two main fields – **MANAGE DATA COLUMNS** and **FILE SELECTION**.

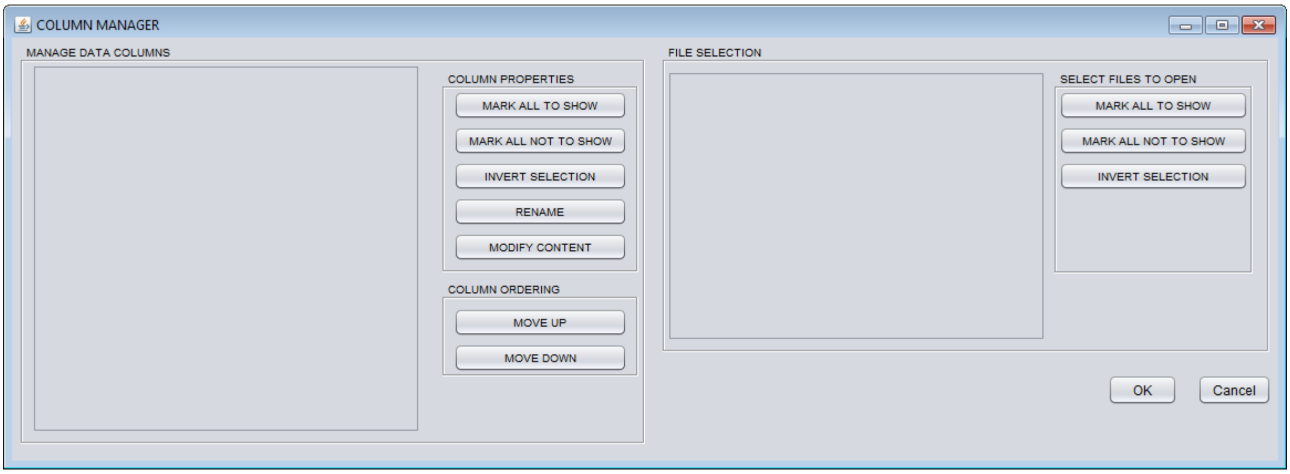

7.5.1 MANAGE DATA COLUMNS field

Here it is possible to change columns' settings / visibility that have been chosen during file(s) import.

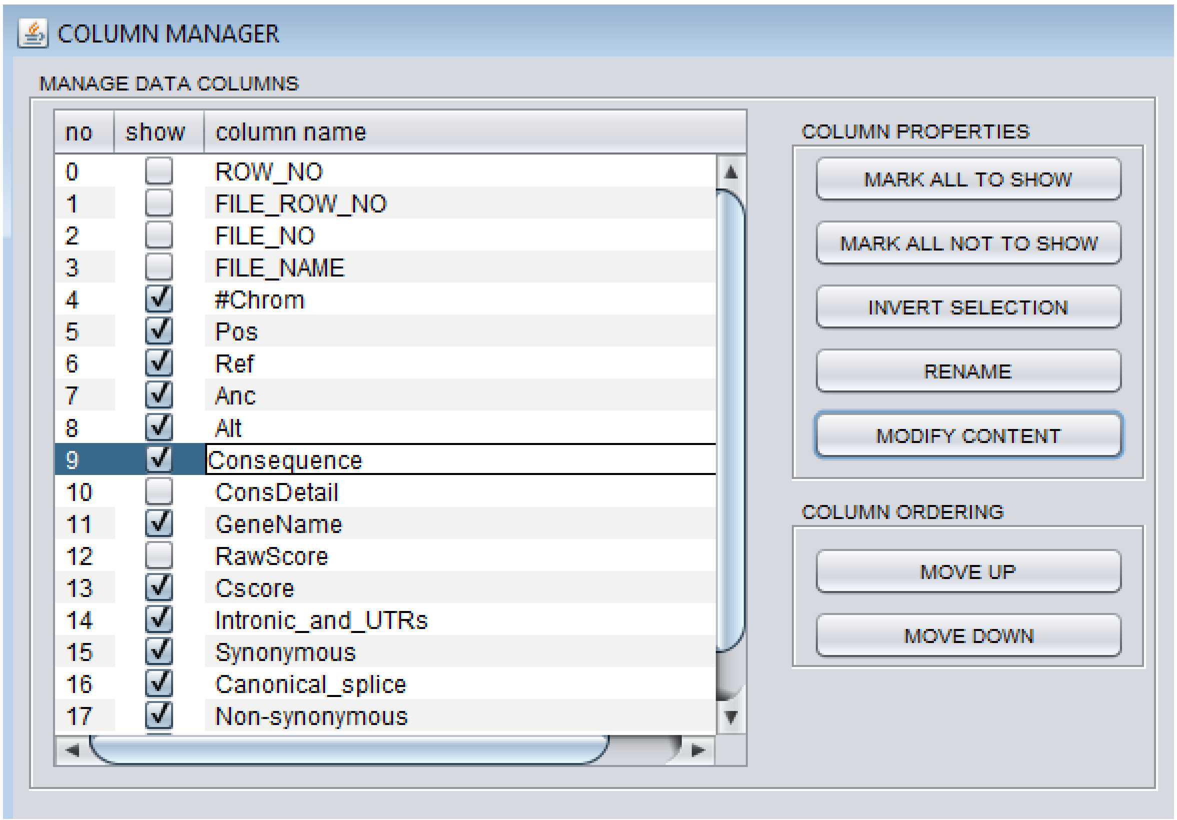

7.5.1.1 COLUMN PROPERTIES subfield

To customize browsing the data by hiding or showing again selected column(s), uncheck or check the corresponding selection field(s). For this purpose also **MARK ALL TO SHOW**, **MARK ALL NOT TO SHOW** and **INVERT SELECTION** buttons can be used. Hidden columns are not deleted from the data set though and are taken into account during every data processing step.

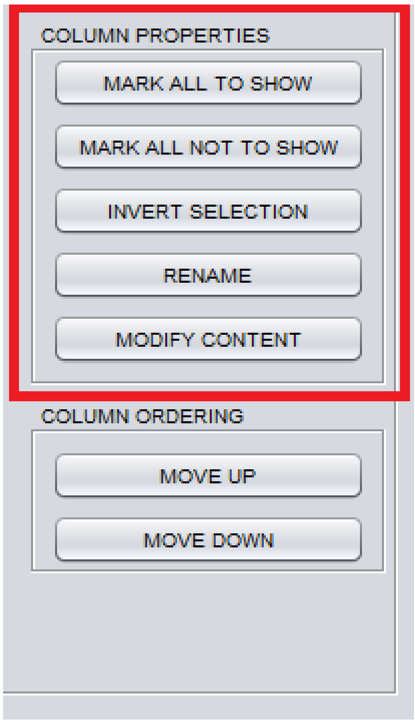

Every column name can be altered in two ways – by double clicking on it or by using the **RENAME** button. The latter opens a new window to define the new name of particular column.

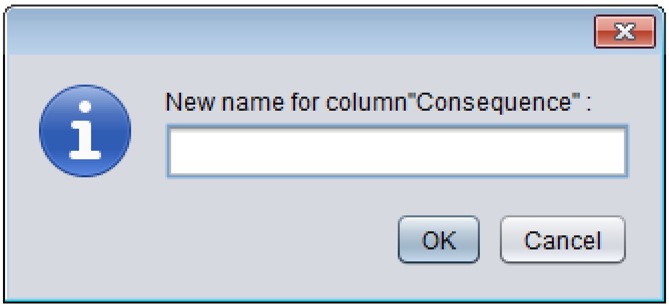

MODIFY CONTENT button opens a window called **COLUMN CONTENT MODIFICIATION**, which is intended to transform the data in all fields / cells from the selected column.

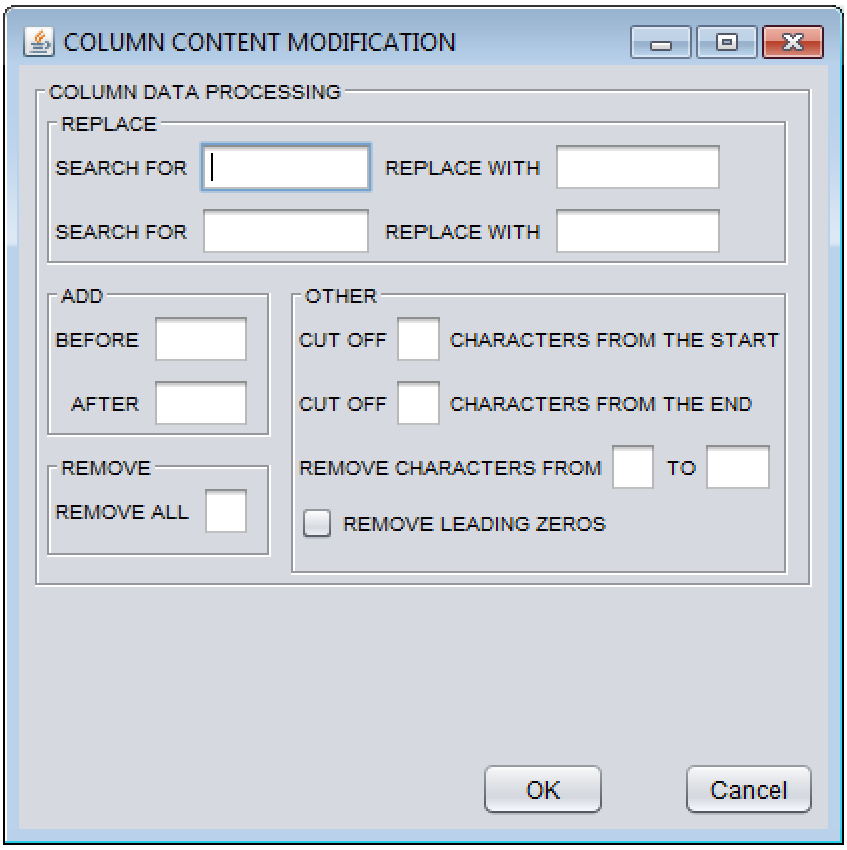

Following changes are available here:

- Searching and replacing alphanumeric strings (**SEARCH FOR** … **REPLACE WITH** …).
- Adding alphanumeric strings before and/or after (**ADD – BEFORE** … and **AFTER** …).
- Removing specified expression from every data row (**REMOVE ALL** …).
- Trimming the ends of the alphanumeric strings (**CUT OFF** … **CHARACTERS FROM THE START** / **END**) or cutting the characters within the alphanumeric strings at the specified positions (**REMOVE CHARACTERS FROM** … **TO** …).
- Removing of so called “leading zeros”, eg. 000023 to 23 (**REMOVE LEADING ZEROS** checkbox).

7.5.1.2 COLUMN ORDERING subfield

The order of columns displayed in **DATA PREVIEW** field (at the main window) can be changed. For this purpose, choose the column name and click **MOVE UP** or **MOVE DOWN** button. Every click changes the column's location by one position forward or backward.

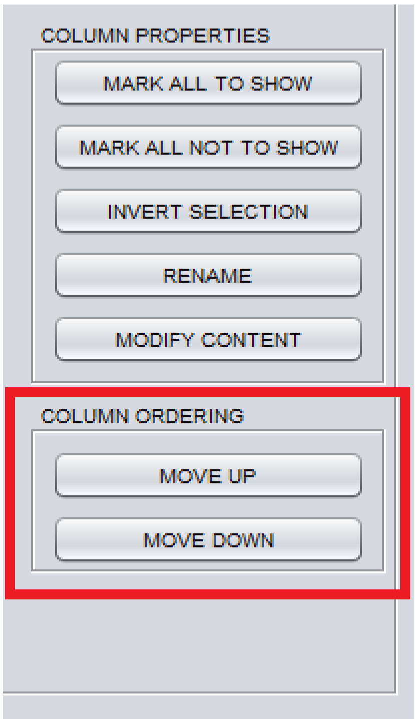

7.5.2 FILE SELECTION field

In this field it is possible to hide or unhide previously imported data set(s). As in case of column selection, mark a checkbox of matched file(s) with cursor or use **MARK ALL TO SHOW**, **MARK ALL NOT TO SHOW** and **INVERT SELECTION** buttons.

Hidden files are not deleted from the data set though, and are taken into account during every data processing step.

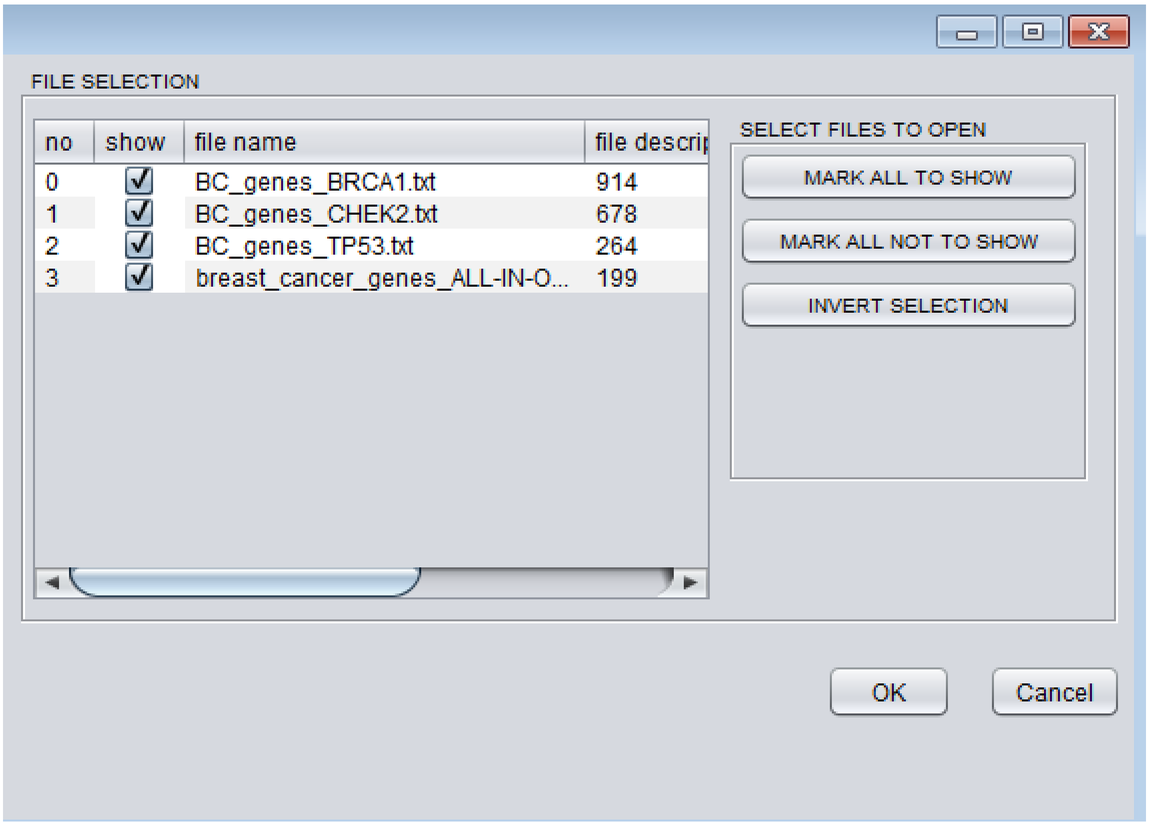

7.6 FILTERING THE INPUT FILE(S)

In the basic mode, filtering applies to the simplified alphanumeric processing of the data.

The **SIMPLE FILTER** window is displayed when the **FILTER** button has been pressed or **Data** > **Filter** in the pull down menu has been selected.

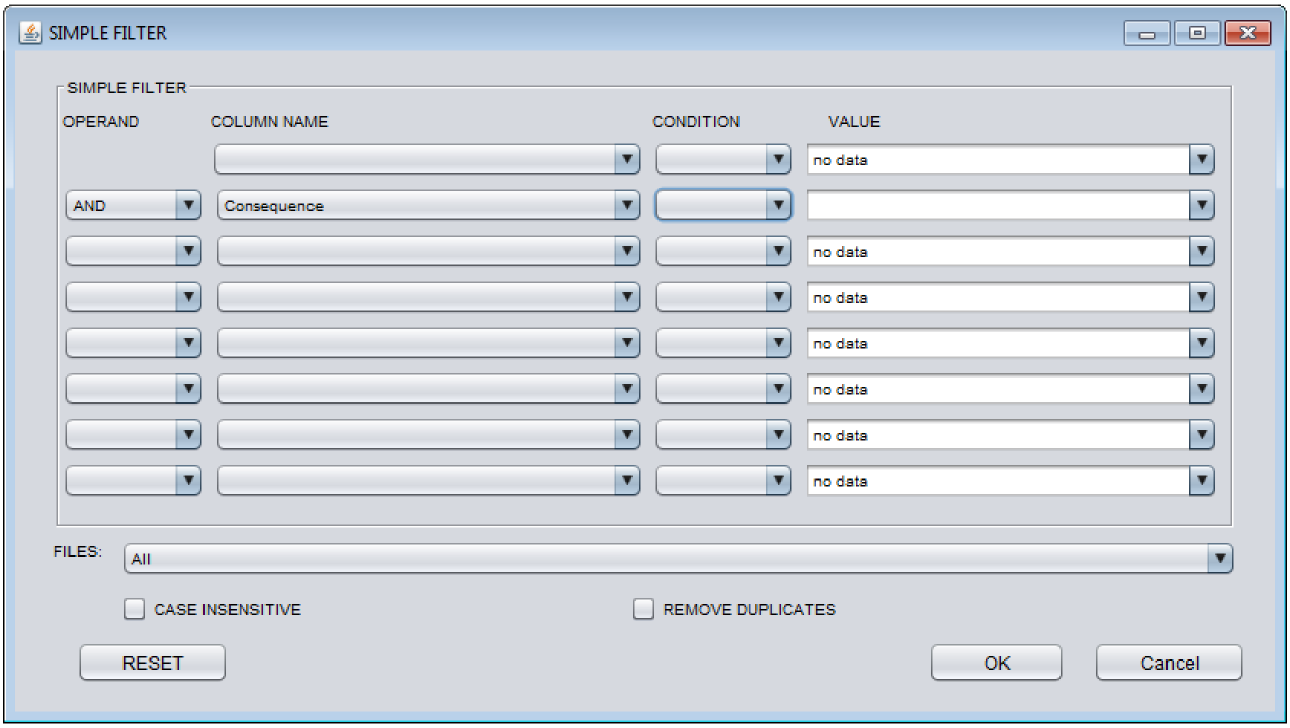

Up to 8 columns, chosen from the **COLUMN NAME** pull down lists, can be filtered. For this purpose following conditions are available in the **CONDITION** pull down list: "**=**", "**<**", "**>**", "**<=**", "**>=**", "**<>**", "**Contains**", "**Does not contain**", "**Begins with**", "**Does not begin with**", "**Ends with**", "**Does not end with**".

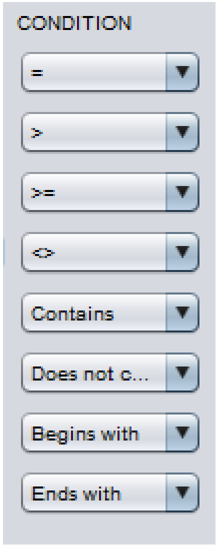

The subsequent filtering steps can be linked by **AND** or **OR** Boolean operators present in **OPERAND** drop-down list at each line.

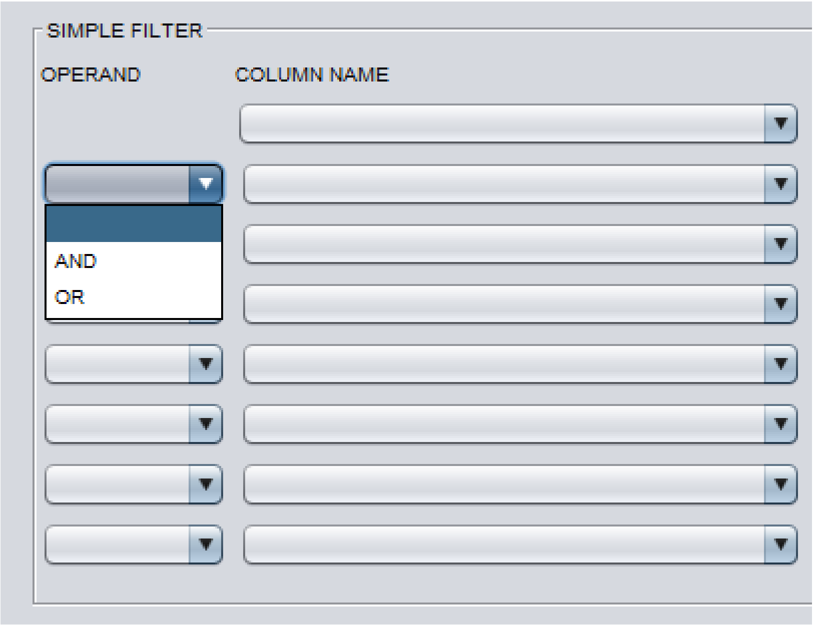

The filtering task can be limited to the selected file with **FILES** drop-down list. By default it is performed for all imported files.

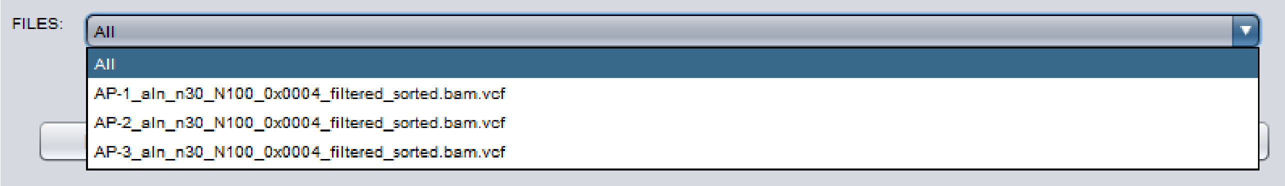

It is also possible to remove duplicate rows from the input data (**REMOVE DUPLICATES** checkbox).

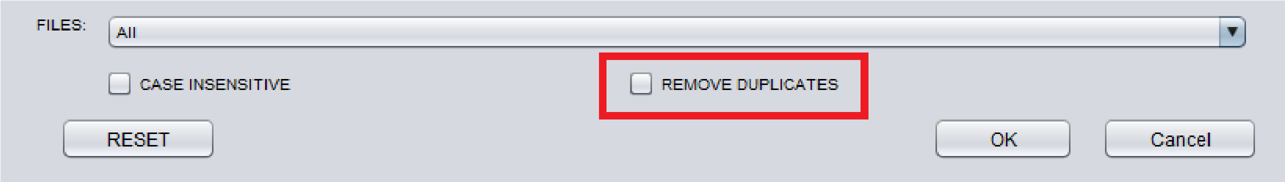

All operations are case sensitive unless specified otherwise by checking the **CASE INSENSITIVE** box.

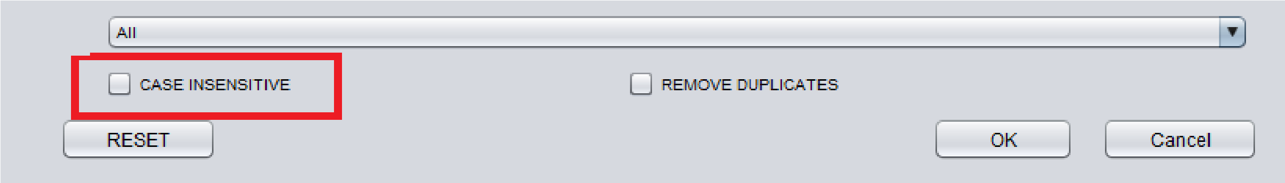

All entered filtering criteria can be cancelled using the **RESET** button.

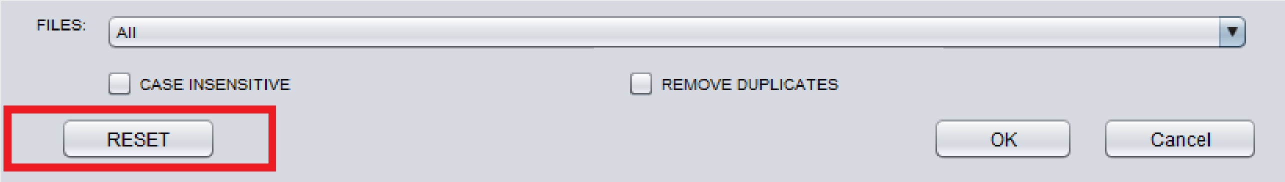

The described functions were used in example 4 (stage 5) in S2 Text. CANCELLING OF INTRODUCED CHANGES

The **RESET** button, which is situated in the operation buttons' panel, restores the data to the original layout. It can be also done using the **Reset all settings** option from the **Edit** pull down menu.

It does not apply to permanently introduced changes – namely using **MODIFY CONTENT** and **RENAME** functions in **COLUMN MANAGER** window (see 7.5.1.1 COLUMN PROPERTIES subfield section).

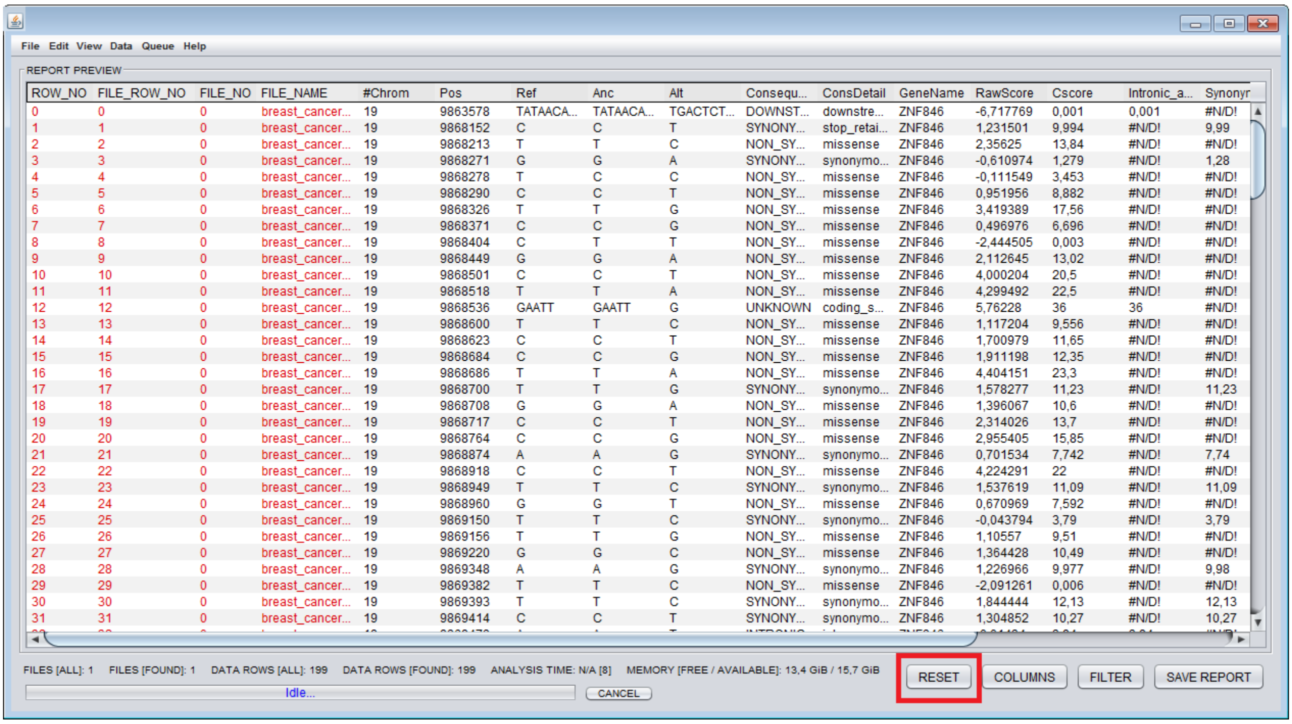

7.7 SAVING FILTERED FILE(S)

The filtered data can be saved to the output file by clicking the **SAVE REPORT** button in the main window or **File > Save Report** in pull down menu.
In the Save window the file directory and name are specified. The file is saved in tab delimited format only.

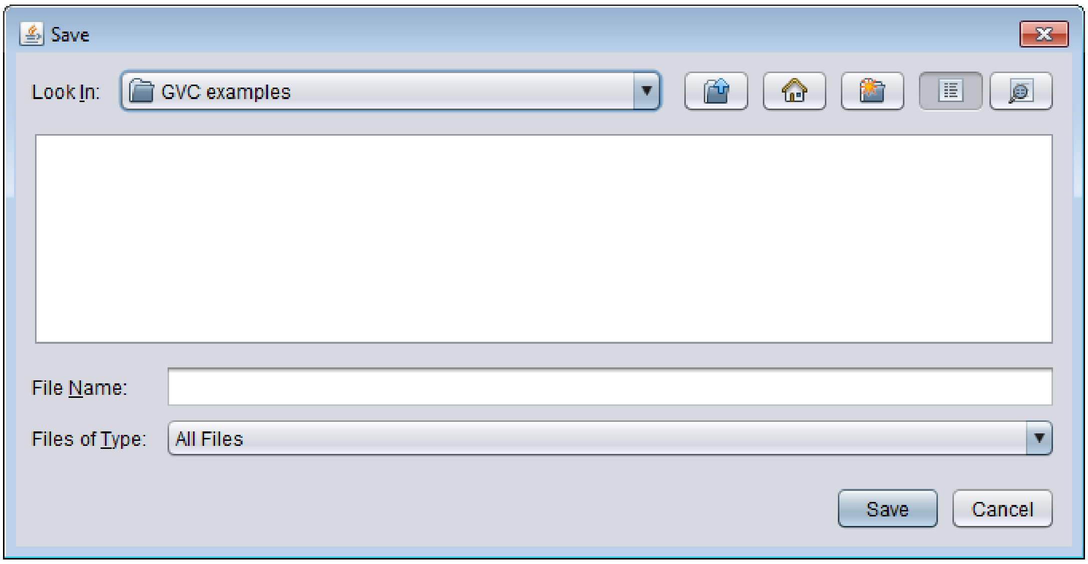

7.8 SAVING QUERIES

Every implemented set of filtering parameters can be saved for further analyses. For this purpose select **Queue** > **Save** in pull down menu in the main window.
In the **SAVE** window the query file directory and name are specified. The file is saved in the internal format (.que extension).

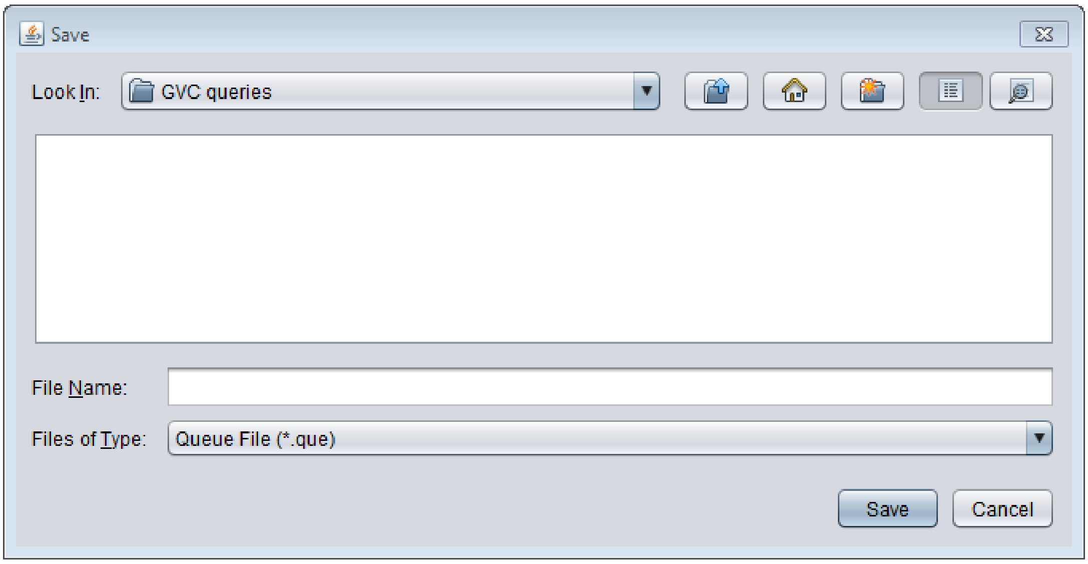

7.9 LOADING QUERIES

Previously saved query can by loaded by selecting **Queue > Load**. The **Load queue file** window is opened to point to the particular directory with .que file.
To successfully use the queue file, it should be consistent with previously loaded data, particularly in terms of columns' names.

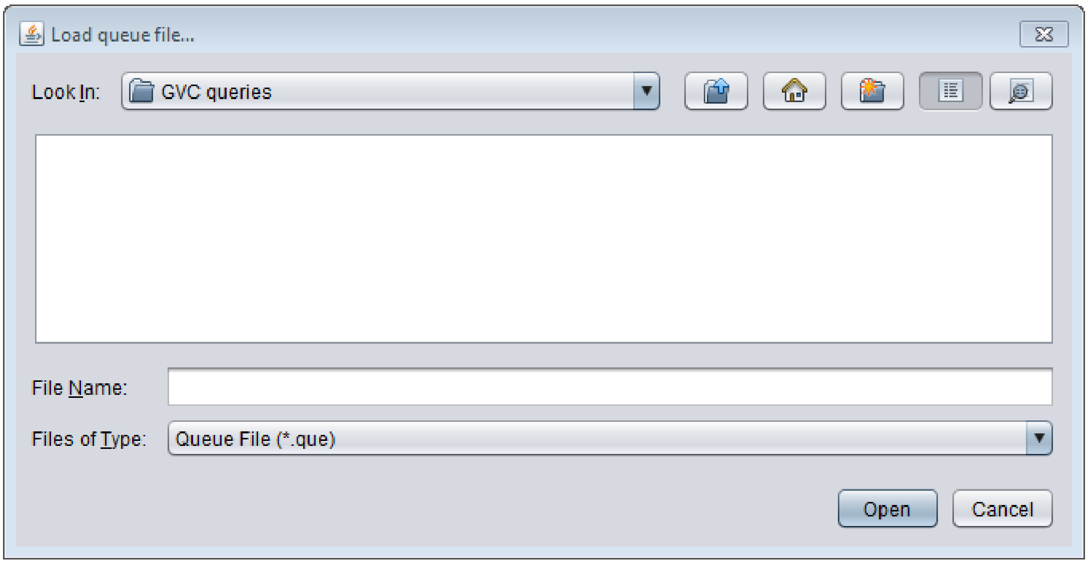

8. ADVANCED MODE – USAGE

The **ADVANCED MODE** is intended for complex tasks that can be performed on the unlimited number of files, simultaneously. For example, the following procedures can be carried out: searching for unique/redundant samples, at the same time for multiple alphanumerical strings, for specified locus, filtering file with regard to any data from external data files or filtering on the base of column defined samples..

8.1 FILE IMPORT

To import file(s) that will be subjected to filtering:

1 Copy the input files to a single directory. Select **File > Open directory** menu option or click on **SELECT DIRECTORY** button and point to this directory in **Select directory** window.
The supported data formats are automatically detected and listed upon data import.

Alternatively, a single file can be imported. To accomplish it, select **File > Open file** menu option or click on **SELECT SINGLE INPUT FILE** button and point to the file in **Select single file** window.

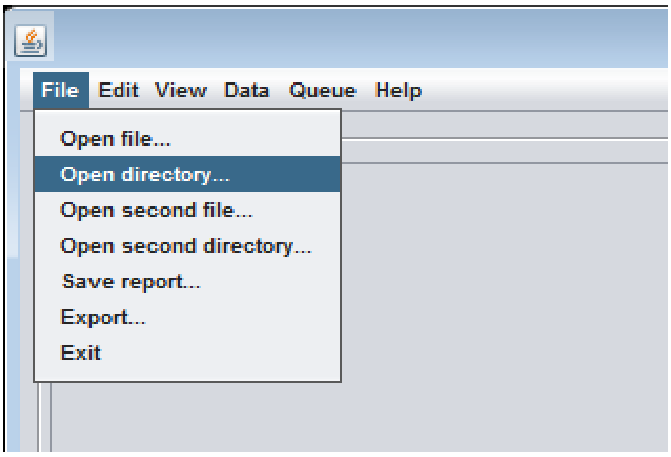

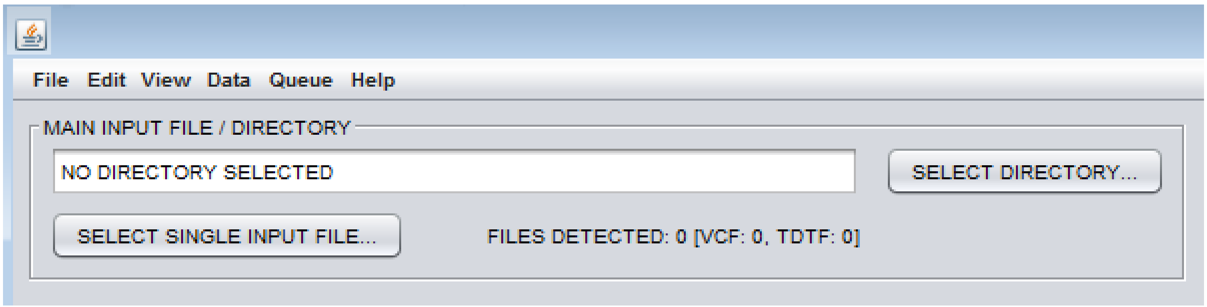

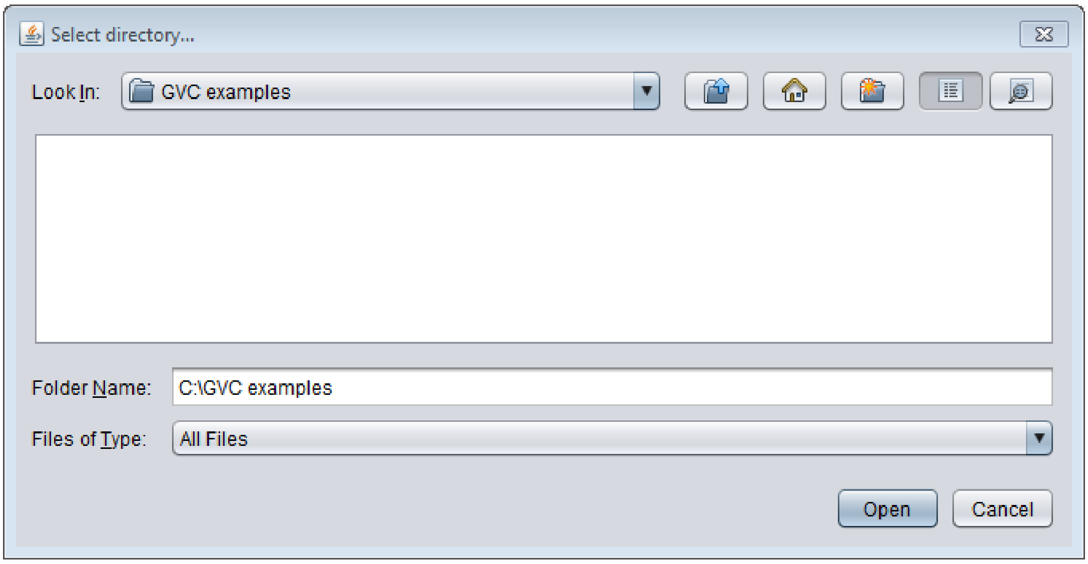

2 The status bar displays file import progress information in real time. The import time depends on the total size of the import files and available computing power and RAM.

3 In the **SELECT FILES** window mark the files from the import directory to open. Using buttons in **SELECT FILES TO OPEN** subfield it is possible to simultaneously select or deselect for opening all detected files (**MARK ALL TO OPEN** / **MARK ALL NOT TO OPEN**) or to invert the selection of files (**INVERT SELECTION**).

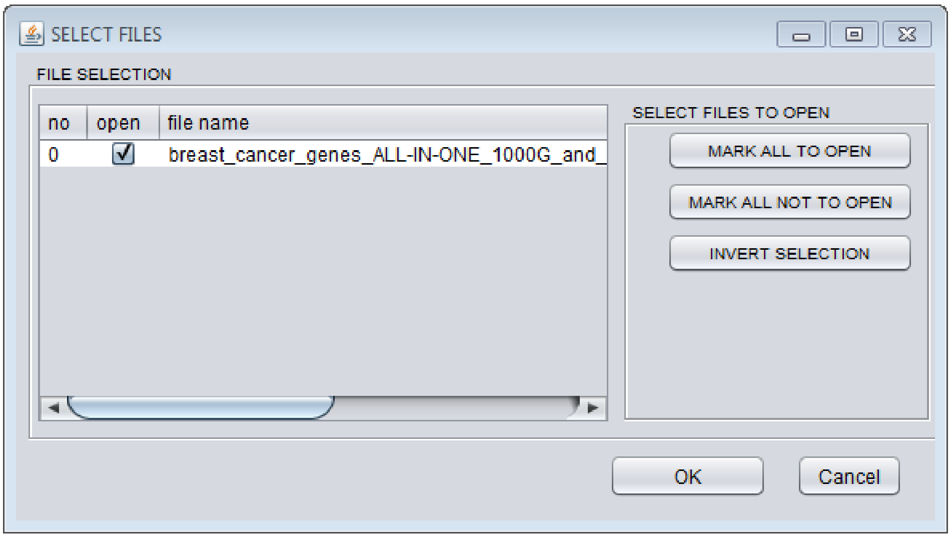

4 In the **SELECT COLUMNS** window select the data columns to be imported from the input files using the same buttons like in the previous window (**MARK ALL TO OPEN, MARK ALL TO NOT OPEN** and **INVERT SELECTION**).

Optionally, the data columns can be reorganized with the **MOVE UP** and **MOVE DOWN** buttons (in **COLUMN ORDERING** subfield).

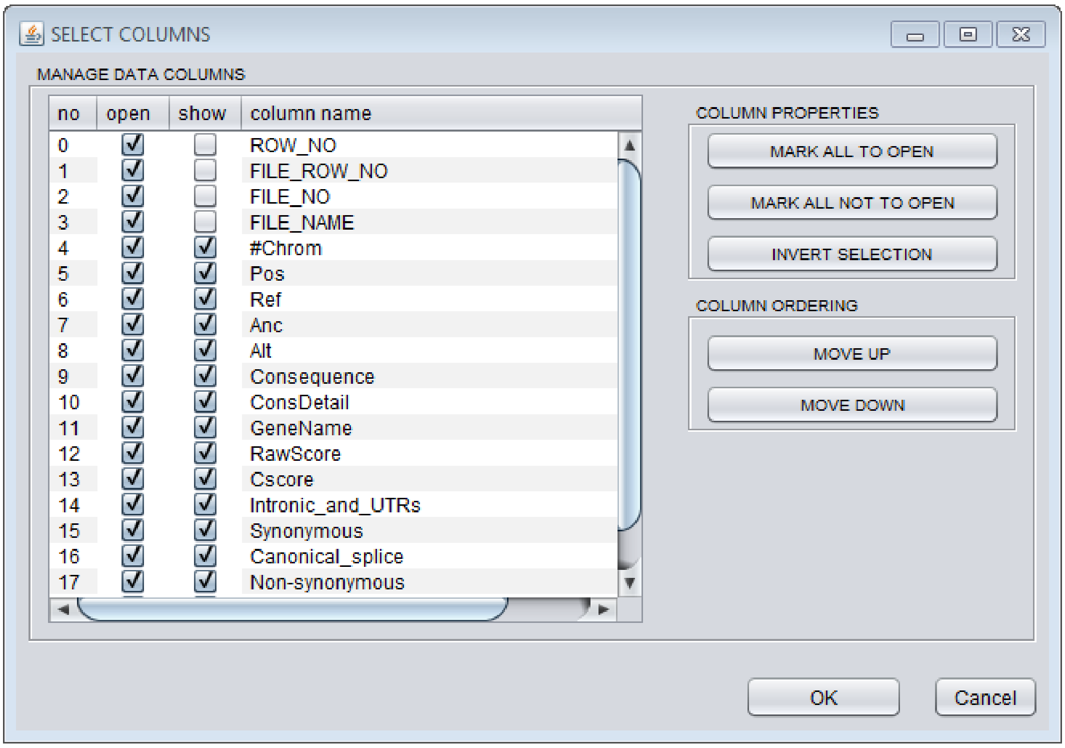

5 When the file import has been completed, the imported files along with the corresponding summaries are displayed in the **FILE LIST** and above the status bar. At the same time the **REPORT PREVIEW** is generated, which contains the combined data from the imported files.

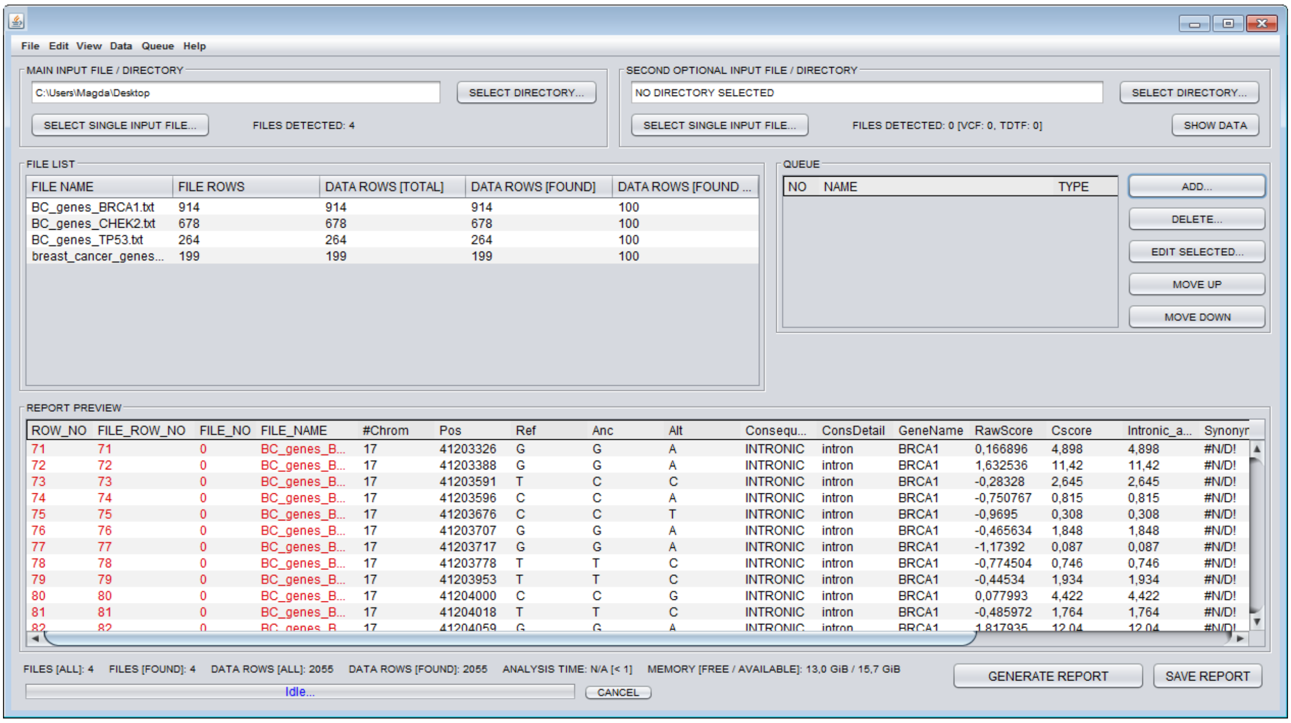

8.2 MAIN VIEW

8.2.1 PULL DOWN MENU

At the top of the main window there is a pull down menu with six program menus, namely **File**, **Edit**, **View**, **Data**, **Queue** and **Help**.

File menu refers to following operations on files – opening a single file, opening a directory with files, opening second file or directory, saving report with processed data and exiting the program.

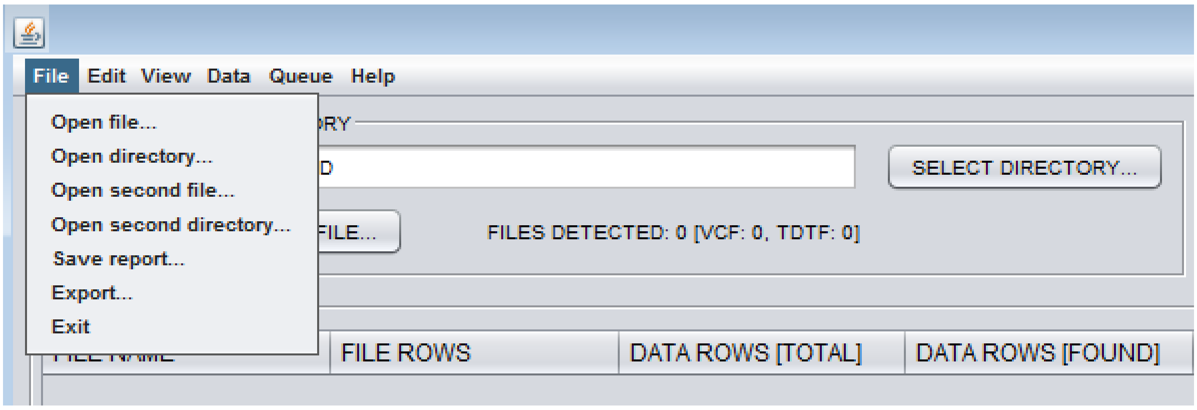

Making use of **Edit** menu, the global data changes can be done – such as carrying out the filtering task and generating a resulting report or resetting all settings.

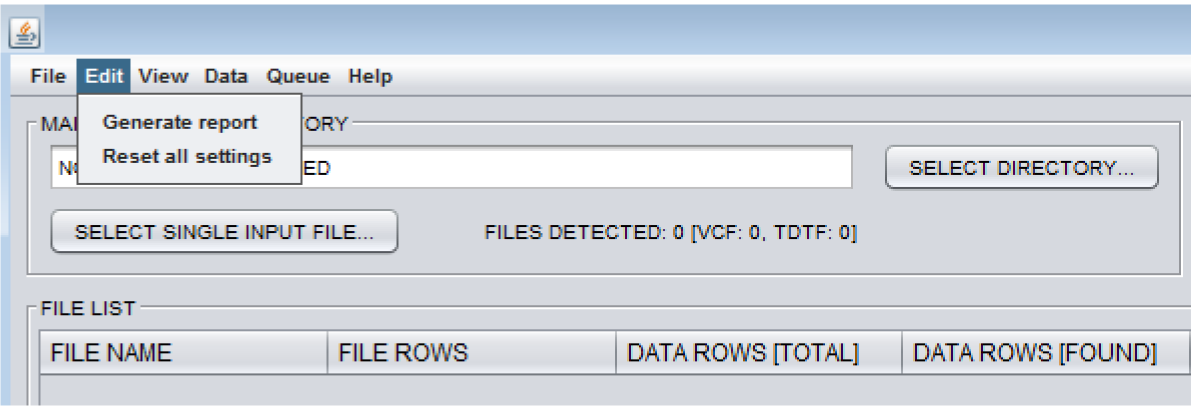

Switching between the advanced and basic mode is available via **View** menu.

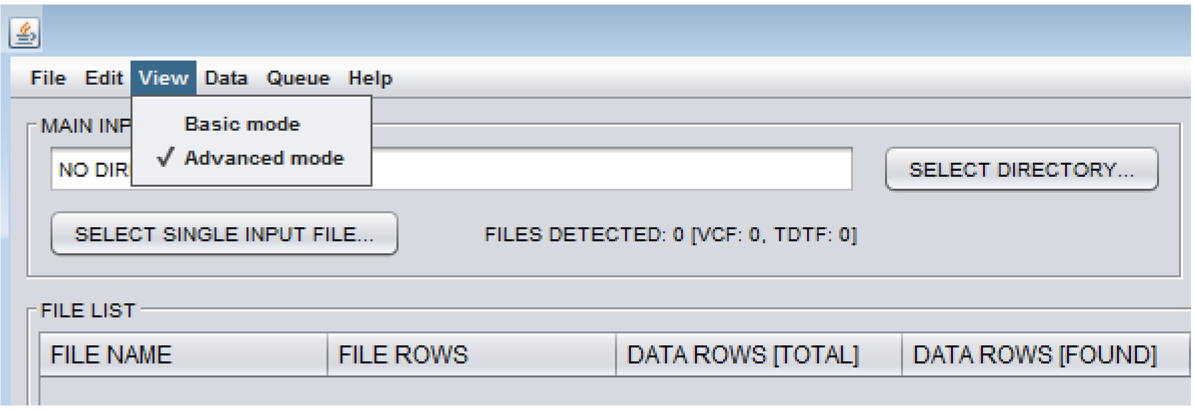

Functions designed for data processing are provided in **Data** menu. Functions called **Column manager**, **Sort** and **Filter** are described in 8.4. COLUMN MANAGEMENT, 8.3. SORTING OF COLUMNS and 8.5. FILTERING THE INPUT FILE(S) section, respectively.

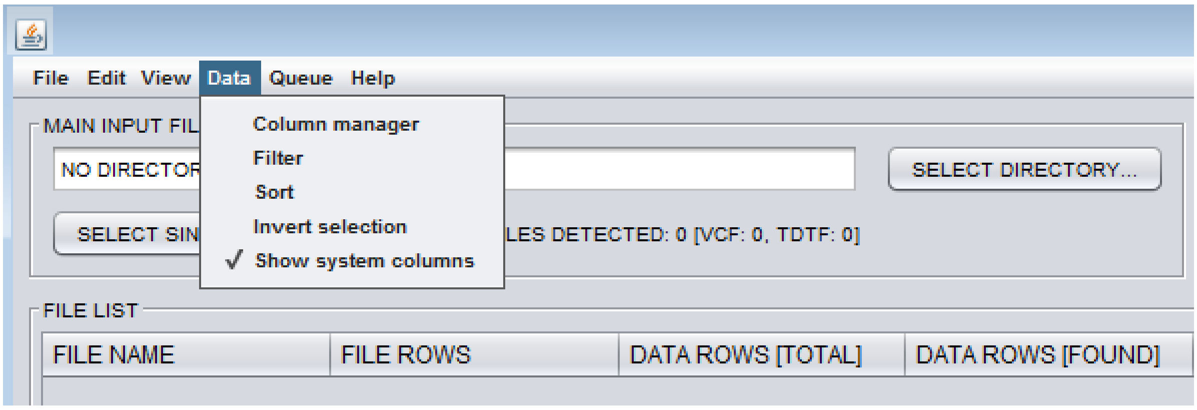

Under **Data** menu it is also possible to reverse the output with regards to the applied filtering criteria using **Invert selection** function. The invert selection function is intended for control purposes, i.e. to provide an insight into data that is removed by the filters.

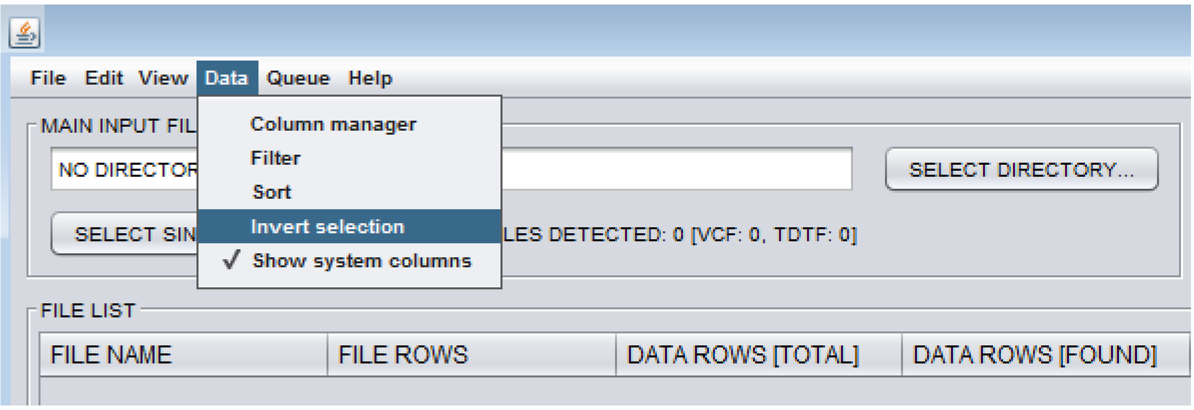

Show system columns function is intended to determine whether system columns are displayed or hidden. System columns, called **ROWS_NO**, **FILE_ROW_NO**, **FILE_NO** and **FILE_NAME**, are created automatically by the program to keep track of the original records in the imported files. **Show system columns** function is mainly helpful when working with multiple files at the same time.

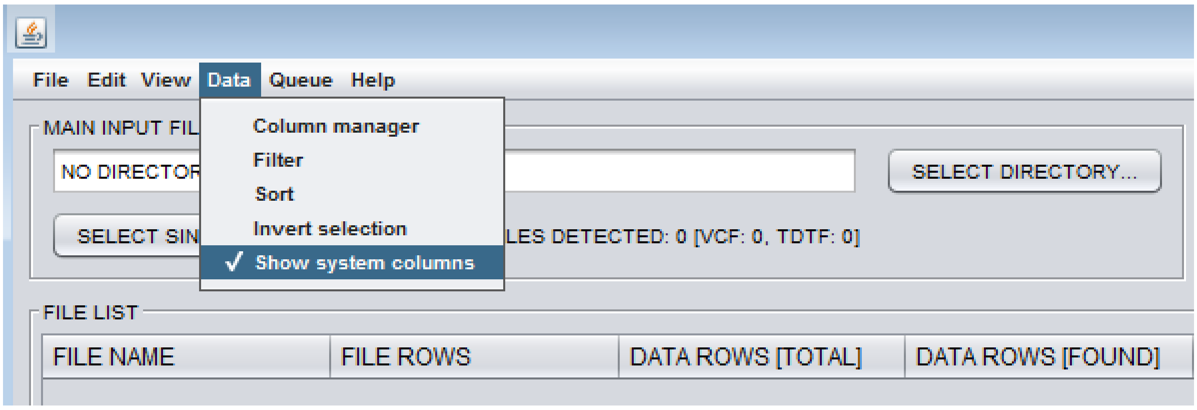

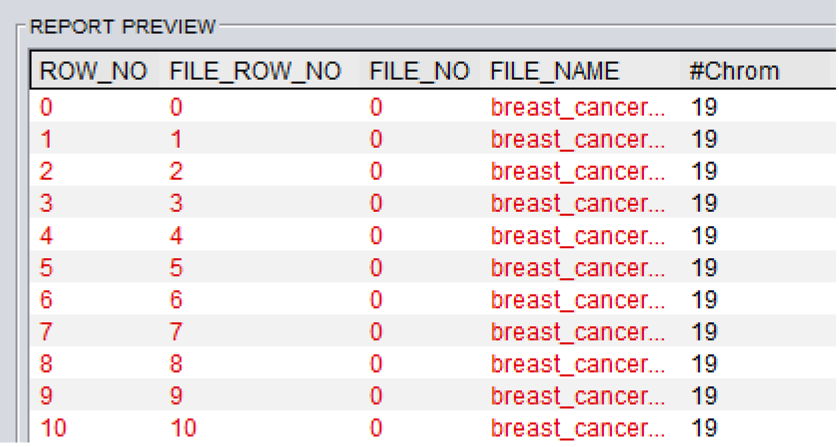

REPORT PREVIEW

ROW_NO	FILE_ROW_NO	FILE_NO	FILE_NAME	#Chrom
0	0	0	breast_cancer...	19
1	1	0	breast_cancer...	19
2	2	0	breast_cancer...	19
3	3	0	breast_cancer...	19
4	4	0	breast_cancer...	19
5	5	0	breast_cancer...	19
6	6	0	breast_cancer...	19
7	7	0	breast_cancer...	19
8	8	0	breast_cancer...	19
9	9	0	breast_cancer...	19
10	10	0	breast_cancer...	19

Add query function under **Queue** menu facilitates adding a query with regard to imported data. It is also possible to save a query consisting of particular parameters for further analyses and to load the previously saved query if needed (see **Error! Reference source not found.** SAVING QUERIES and 7.9 . LOADING QUERIES sections).

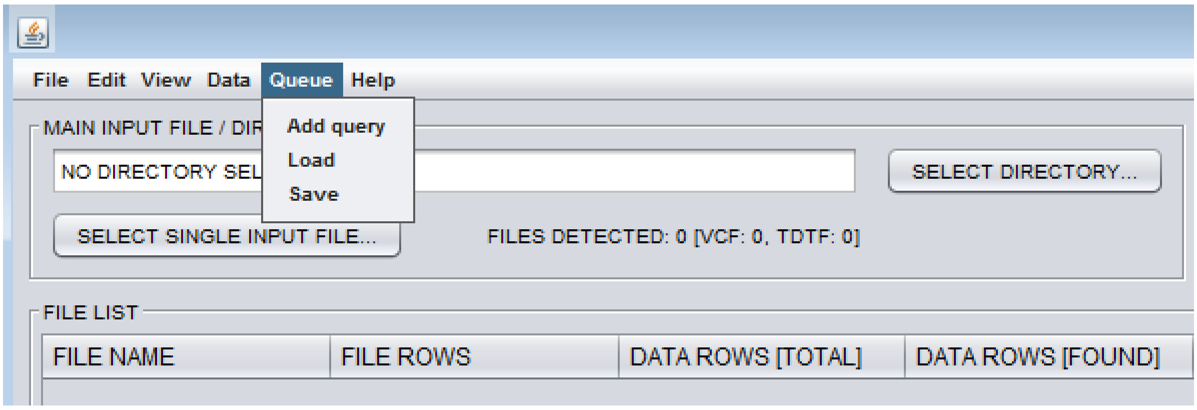

Information about the **HIGH-THROUGHPUT TABULAR DATA PROCESSOR** and this **user manual** is accessible via **Help** menu.

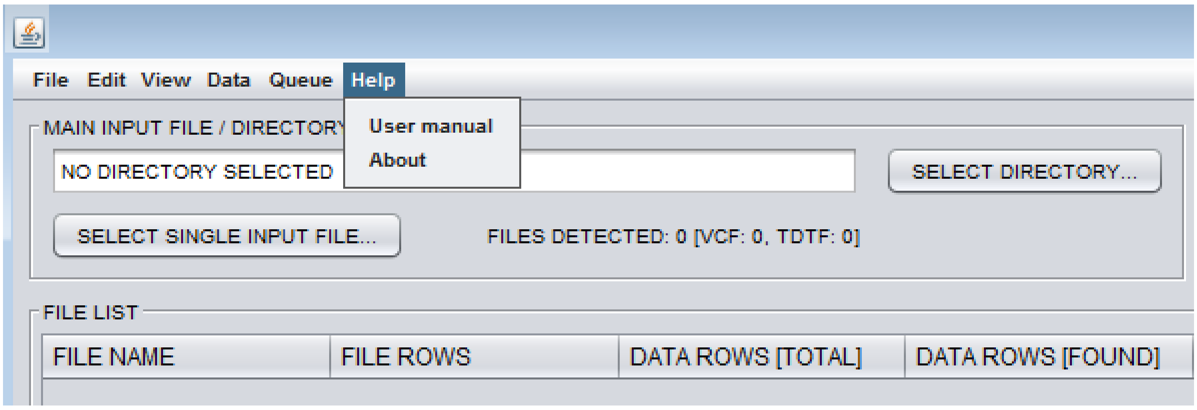

8.2.2 MAIN INPUT FILE / DIRECTORY field

This field facilities opening a single file (**SELECT SINGLE INPUT FILE**) or directory with files (**SELECT DIRECTORY**) intended for processing. For details see **Error! Reference source not found.** FILE IMPORT section.

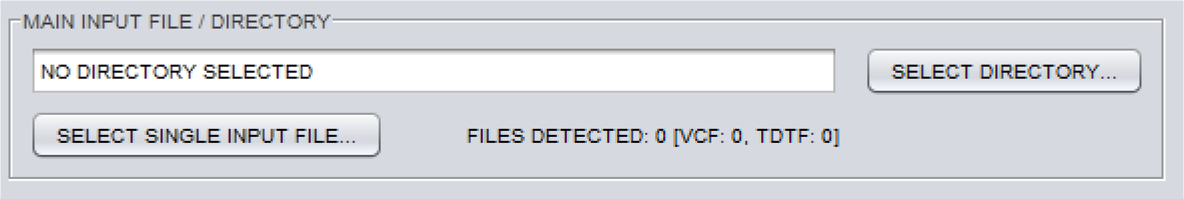

8.2.3 SECOND, OPTIONAL FILE / DIRECTORY field

Here, it is possible to open an additional file (**SELECT SINGLE INPUT FILE**) or directory with files (**SELECT DIRECTORY**). All operations are similar to those in the **MAIN INPUT FILE / DIRECTORY** field.

The **SHOW DATA** button provides a preview of the new data set.

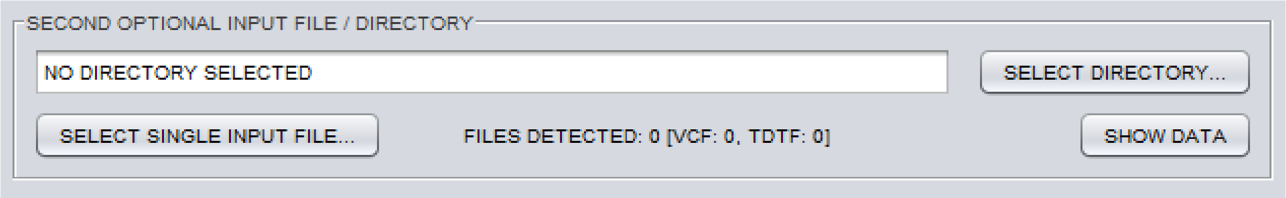

8.2.4 FILE LIST field

This field contains a list of imported files. Besides files names (**FILE NAME** column), the following information is provided:

- The number of rows in the input file (**FILE ROWS**).
- The total number of data rows (**DATA ROWS [TOTAL]**) – in the case of tab delimited text files the values in **FILE ROWS** and **DATA ROWS [TOTAL]** are always equal, while for VCF files these values may be different.
- The number of rows which are retained after the filtering criteria have been applied (**DATA ROWS [FOUND]**).
- Percentage of rows which are retained after the filtering criteria have been applied (**DATA ROWS [FOUND (%)]**), i.e. DATA ROWS [FOUND] vs. DATA ROWS [TOTAL].

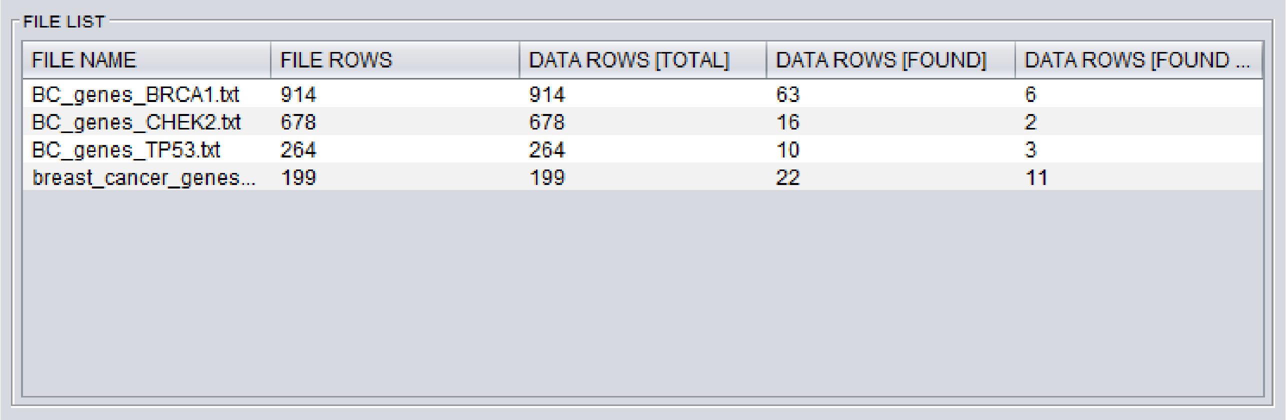
FILE LIST

FILE NAME	FILE ROWS	DATA ROWS [TOTAL]	DATA ROWS [FOUND]	DATA ROWS [FOUND ...
BC_genes_BRCA1.txt	914	914	63	6
BC_genes_CHEK2.txt	678	678	16	2
BC_genes_TP53.txt	264	264	10	3
breast_cancer_genes...	199	199	22	11

The list is updated in real time along with execution of user queries (see **Error! Reference source not found.** FILTERING THE INPUT FILE(S) section). It can be also sorted by any column after clicking the column name (see **Error! Reference source not found.** SORTING OF COLUMNS section).

8.2.5 QUEUE field

8.2.5.1 QUEUE subfield

This subfield displays the list of user-defined queries.

Each query is automatically denoted by subsequent number (**NO** column) along with user-defined identifier (**NAME**). Each query is also tagged with a query type (**TYPE** column) according to the name of the tab in **QUERY CONFIGURATION** window containing filtering parameters (see **Error! Reference source not found.** QUERY CONFIGURATION WINDOW – ADVANCED MODE section).
Queries are implemented in the order in which they have been added to the list. There are no limits with regard to the number of queries.

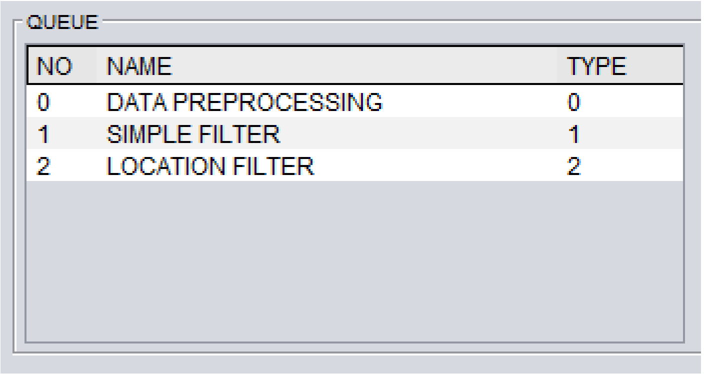

8.2.5.2 QUERY QUEUE CONTROL SECTION

Five buttons located on the right side of the **QUEUE** subfield allow query queue management.

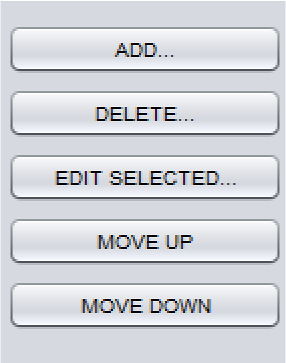

ADD button opens query configuration window to set filtering rules (see Error! Reference source not found. FILTERING THE INPUT FILE(S) section). Subsequently added queries will show up in the list.

Removal of every added query is possible using the **DELETE** button.

Click on a specific query in **QUEUE** field and then on **EDIT SELECTED** button to modify the content of this query. Afterwards, the **QUERY SETUP** window will be displayed again to introduce all changes.

Using **MOVE UP** and **MOVE DOWN** buttons the order of queries in the queue can be changed.

8.2.6 REPORT PREVIEW

The **REPORT PREVIEW** field displays the preview of the resulting data table.

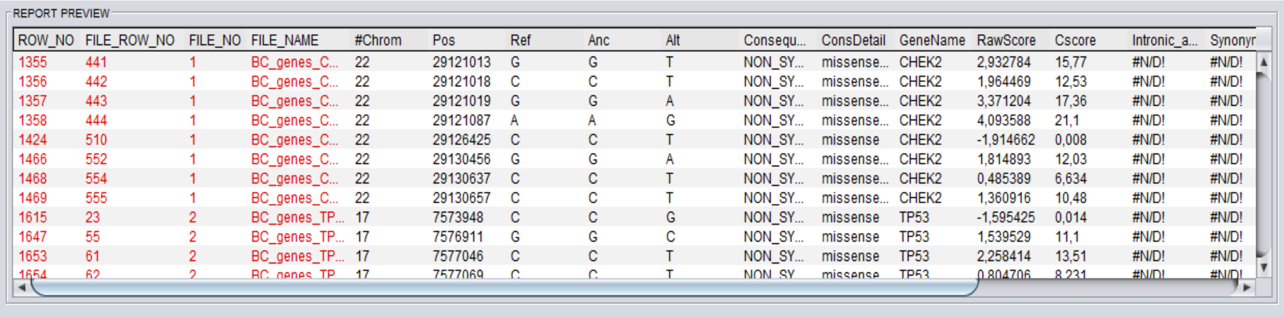

REPORT PREVIEW

ROW_NO	FILE_ROW_NO	FILE_NO	FILE_NAME	#Chrom	Pos	Ref	Anc	Alt	Consequ...	ConsDetail	GeneName	RawScore	Cscore	Intronic_a...	Synonyr
1355	441	1	BC_genes_C...	22	29121013	G	G	T	NON_SY...	missense...	CHEK2	2,932784	15,77	#N/D!	#N/D!
1356	442	1	BC_genes_C...	22	29121018	C	C	T	NON_SY...	missense...	CHEK2	1,964469	12,53	#N/D!	#N/D!
1357	443	1	BC_genes_C...	22	29121019	G	G	A	NON_SY...	missense...	CHEK2	3,371204	17,36	#N/D!	#N/D!
1358	444	1	BC_genes_C...	22	29121087	A	A	G	NON_SY...	missense...	CHEK2	4,093588	21,1	#N/D!	#N/D!
1424	510	1	BC_genes_C...	22	29126425	C	C	T	NON_SY...	missense	CHEK2	-1,914662	0,008	#N/D!	#N/D!
1466	552	1	BC_genes_C...	22	29130456	G	G	A	NON_SY...	missense...	CHEK2	1,814893	12,03	#N/D!	#N/D!
1468	554	1	BC_genes_C...	22	29130637	C	C	T	NON_SY...	missense...	CHEK2	0,485389	6,634	#N/D!	#N/D!
1469	555	1	BC_genes_C...	22	29130657	C	C	T	NON_SY...	missense...	CHEK2	1,360916	10,48	#N/D!	#N/D!
1615	23	2	BC_genes_TP...	17	7573948	C	C	G	NON_SY...	missense	TP53	-1,595425	0,014	#N/D!	#N/D!
1647	55	2	BC_genes_TP...	17	7576911	G	G	C	NON_SY...	missense	TP53	1,539529	11,1	#N/D!	#N/D!
1653	61	2	BC_genes_TP...	17	7577046	C	C	T	NON_SY...	missense	TP53	2,258414	13,51	#N/D!	#N/D!
1654	62	2	BC_genes_TP...	17	7577069	C	C	T	NON_SY...	missense	TP53	0,804706	8,231	#N/D!	#N/D!

This table is updated in real time along with execution of the filtering (see **Error! Reference source not found.** FILTERING THE INPUT FILE(S) section). It can be sorted by any column after clicking the column name (see **Error! Reference source not found.** SORTING OF COLUMNS section).

This table is also editable – double click on certain field, type some text and modified content will be saved.

8.2.7 INFORMATION AND STATUS BAR

The information bar, located at the bottom of the main window, shows aggregate data statistics:

- Number of imported files (**FILES [ALL]**).
- Number of imported files which have retained at least one row of data after the filtering has been applied (**FILES [FOUND]**).
- The total number of rows in all imported files prior to filtering (**DATA ROWS [ALL]**).
- The total number of rows in all imported files which are retained after the filtering criteria have been applied (**DATA ROWS [FOUND]**).
- Total analysis time after completion of all items in the query queue (**ANALYSIS TIME**).

Status bar is located below aggregate data statistics and shows progress of the analysis in real time.

The **CANCEL** button allows user to abort an ongoing operation.

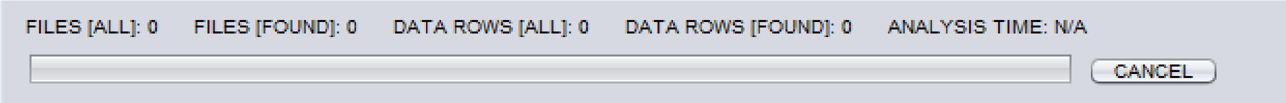

8.2.8 FILE OPERATIONS BUTTONS

GENERATE REPORT button commences data analysis. The status bar will display data processing progress information in real time and after completion the **REPORT PREVIEW** will be refreshed.

SAVE REPORT opens a dialog box to specify the output file name and folder (see **Error! Reference source not found.** SAVING FILTERED FILE(S) section). The output data is saved as tab delimited file.

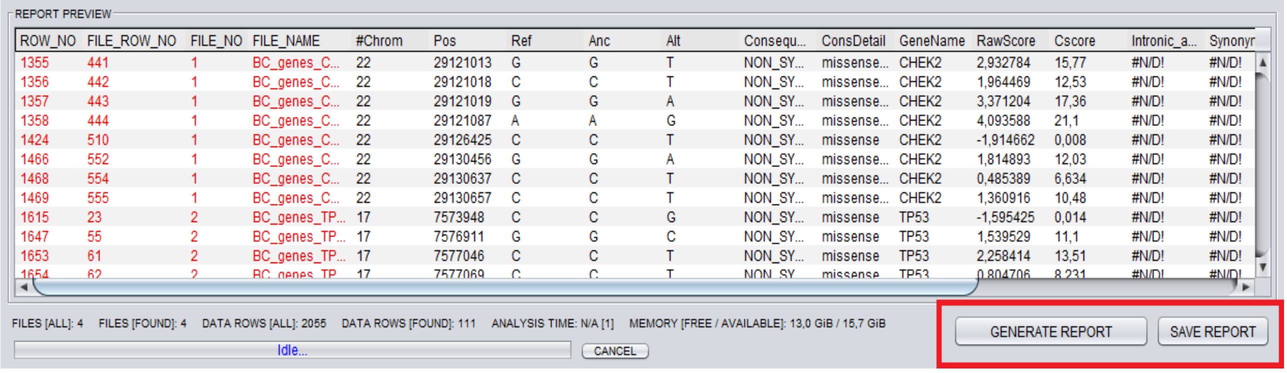

ROW_NO	FILE_ROW_NO	FILE_NO	FILE_NAME	#Chrom	Pos	Ref	Anc	Alt	Consequ...	ConsDetail	GeneName	RawScore	Cscore	Intronic_a...	Synonyr
1355	441	1	BC_genes_C...	22	29121013	G	G	T	NON_SY...	missense...	CHEK2	2,932784	15,77	#N/D!	#N/D!
1356	442	1	BC_genes_C...	22	29121018	C	C	T	NON_SY...	missense...	CHEK2	1,964469	12,53	#N/D!	#N/D!
1357	443	1	BC_genes_C...	22	29121019	G	G	A	NON_SY...	missense...	CHEK2	3,371204	17,36	#N/D!	#N/D!
1358	444	1	BC_genes_C...	22	29121087	A	A	G	NON_SY...	missense...	CHEK2	4,093588	21,1	#N/D!	#N/D!
1424	510	1	BC_genes_C...	22	29126425	C	C	T	NON_SY...	missense	CHEK2	-1,914662	0,008	#N/D!	#N/D!
1466	552	1	BC_genes_C...	22	29130456	G	G	A	NON_SY...	missense...	CHEK2	1,814893	12,03	#N/D!	#N/D!
1468	554	1	BC_genes_C...	22	29130637	C	C	T	NON_SY...	missense...	CHEK2	0,485389	6,634	#N/D!	#N/D!
1469	555	1	BC_genes_C...	22	29130657	C	C	T	NON_SY...	missense...	CHEK2	1,360916	10,48	#N/D!	#N/D!
1615	23	2	BC_genes_TP...	17	7573948	C	C	G	NON_SY...	missense	TP53	-1,595425	0,014	#N/D!	#N/D!
1647	55	2	BC_genes_TP...	17	7576911	G	G	C	NON_SY...	missense	TP53	1,539529	11,1	#N/D!	#N/D!
1653	61	2	BC_genes_TP...	17	7577046	C	C	T	NON_SY...	missense	TP53	2,258414	13,51	#N/D!	#N/D!
1654	62	2	BC_genes_TP...	17	7577069	C	C	T	NON_SY...	missense	TP53	0,804706	8,231	#N/D!	#N/D!

8.3 SORTING OF COLUMNS

To rearrange the data set with respect to one or more columns, select **Data > Sort** in pull down menu or click on any column name in the header line in the **REPORT PREVIEW** field. The **DATA SORT** window which is opened, facilitates sorting by up to the three columns at a time, in ascending or descending manner and in case insensitive (by default) or case sensitive way.

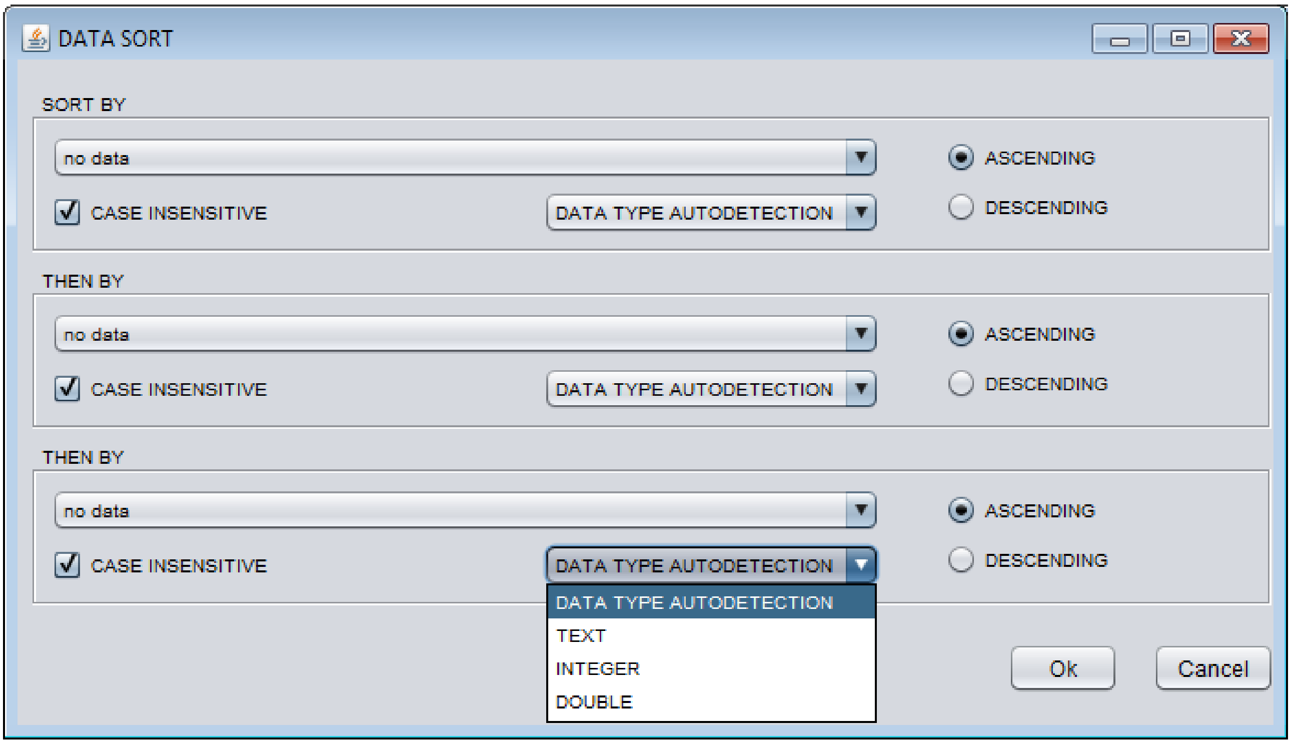

The type of data in the column(s) subjected to sorting is detected automatically (**DATA TYPE AUTODETECTION**), but can be also selected by hand as **TEXT**, **INTEGER** or **DOUBLE** (double-precision floating-point numbers, namely decimals).COLUMNS MANAGEMENT

Column(s) and file(s) management is possible via **COLUMN MANAGER** window. To open the window select **Data** > **Column manager** in pull down menu.

This window consists of two fields – **MANAGE DATA COLUMNS** and **FILE SELECTION**.

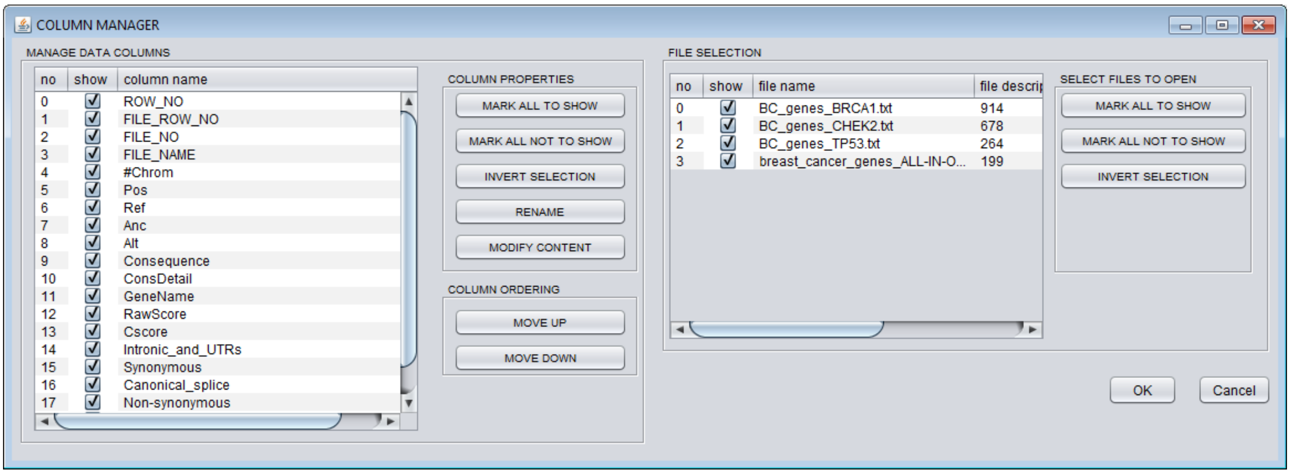
COLUMN MANAGER
MANAGE DATA COLUMNS
no
show
column name
0 ROW_NO
1 FILE_ROW_NO
2 FILE_NO
3 FILE_NAME
4 #Chrom
5 Pos
6 Ref
7 Anc
8 Alt
9 Consequence
10 ConsDetail
11 GeneName
12 RawScore
13 Cscore
14 Intronic_and_UTRs
15 Synonymous
16 Canonical_splice
17 Non-synonymous
COLUMN PROPERTIES
MARK ALL TO SHOW
MARK ALL NOT TO SHOW
INVERT SELECTION
RENAME
MODIFY CONTENT
COLUMN ORDERING
MOVE UP
MOVE DOWN
FILE SELECTION
no
show
file name
file descrip
0 BC_genes_BRCA1.txt 914
1 BC_genes_CHEK2.txt 678
2 BC_genes_TP53.txt 264
3 breast_cancer_genes_ALL-IN-O... 199
SELECT FILES TO OPEN
MARK ALL TO SHOW
MARK ALL NOT TO SHOW
INVERT SELECTION
OK
Cancel

8.3.1 MANAGE DATA COLUMNS field

Here it is possible to change columns' settings that have been chosen during file(s) import.

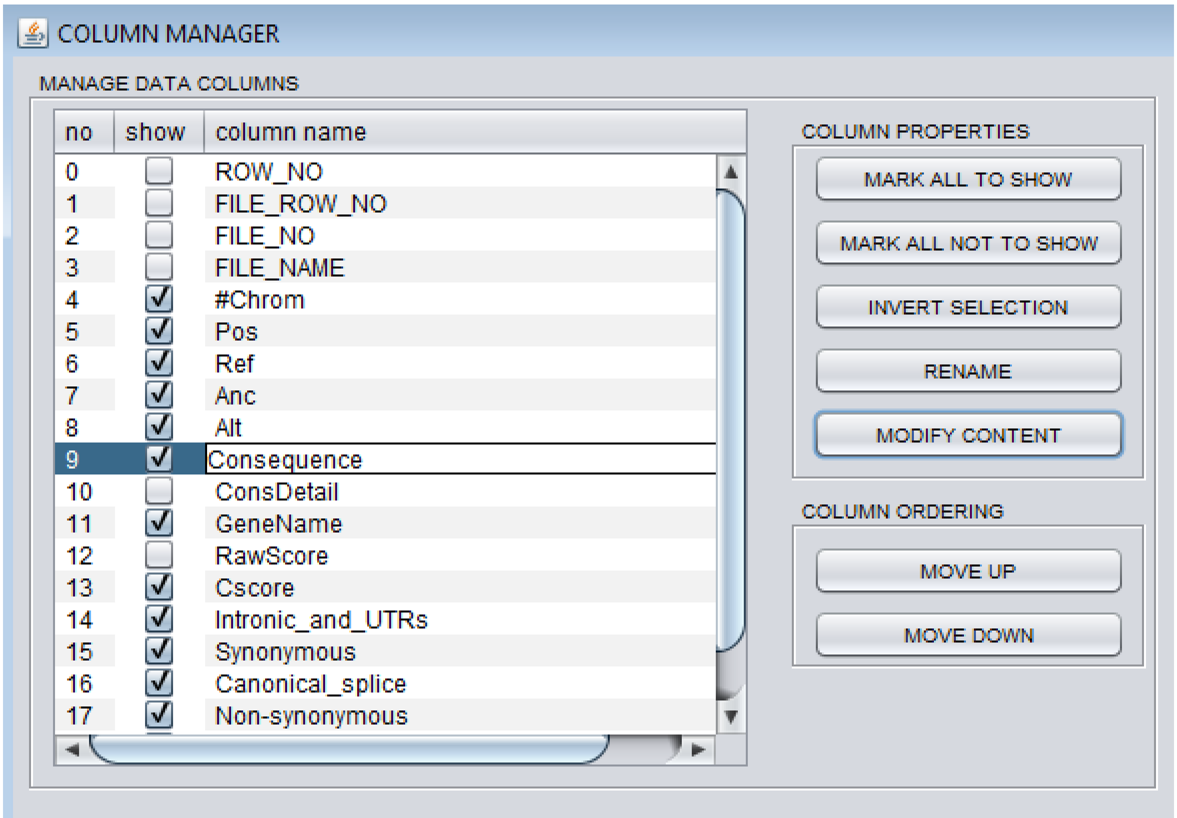

8.3.1.1 COLUMN PROPERTIES subfield

To customize browsing the data by hiding or showing again selected column(s), uncheck or check the corresponding selection field(s). For this purpose also **MARK ALL TO SHOW**, **MARK ALL NOT TO SHOW** and **INVERT SELECTION** buttons can be used. Hidden columns are not deleted from the data set though and are taken into account during every data processing step.

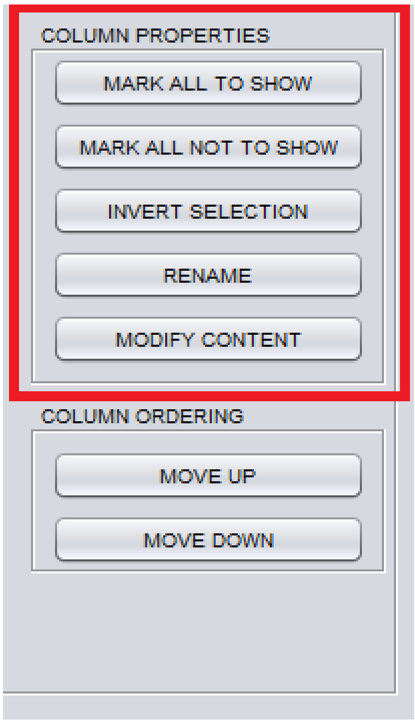

Every column name can be altered in two ways – by double clicking on it or by using the **RENAME** button. The latter opens the next window to define the new name of a specific column.

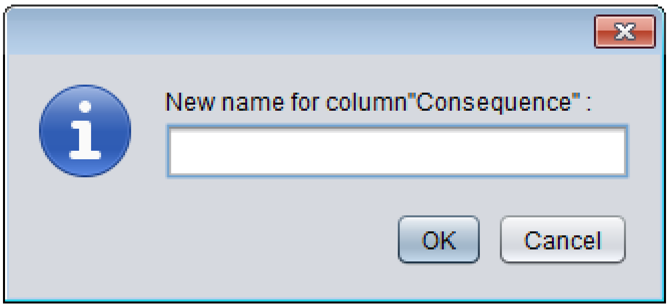

MODIFY CONTENT button opens a window called **COLUMN CONTENT MODIFICIATION,** which is intended to transform the data in all fields / cells from the selected column.

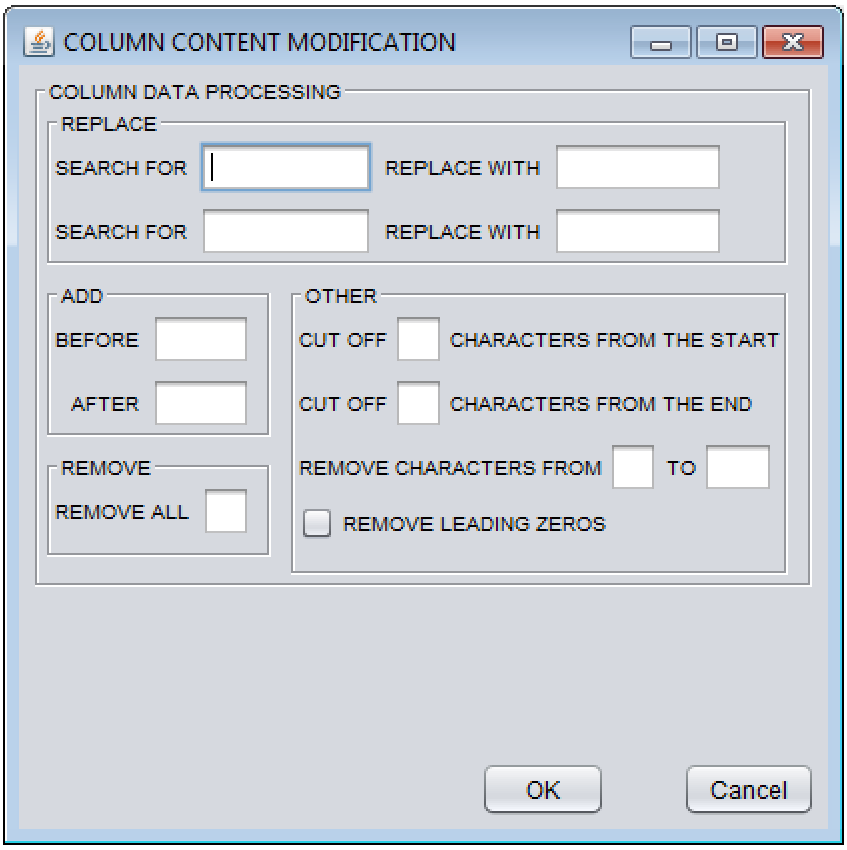

Following changes are available here:

- Searching and replacing alphanumeric strings (**SEARCH FOR** … **REPLACE WITH** …).
- Adding alphanumeric strings before and/or after (**ADD** – **BEFORE** … and **AFTER** …).
- Removing specified expression from every data row (**REMOVE ALL** …).
- Trimming the ends of the alphanumeric strings (**CUT OFF** … **CHARACTERS FROM THE START** / **END**) or cutting the characters within the alphanumeric strings at the specified positions (**REMOVE CHARACTERS FROM** … **TO** …).
- Removing of so called “leading zeros”, eg. 000023 to 23 (**REMOVE LEADING ZEROS** checkbox).

8.3.1.2 COLUMN ORDERING subfield

The order of columns displayed in **DATA PREVIEW** field (at the main window) can be changed. For this purpose, point the column name and click **MOVE UP** or **MOVE DOWN** button.

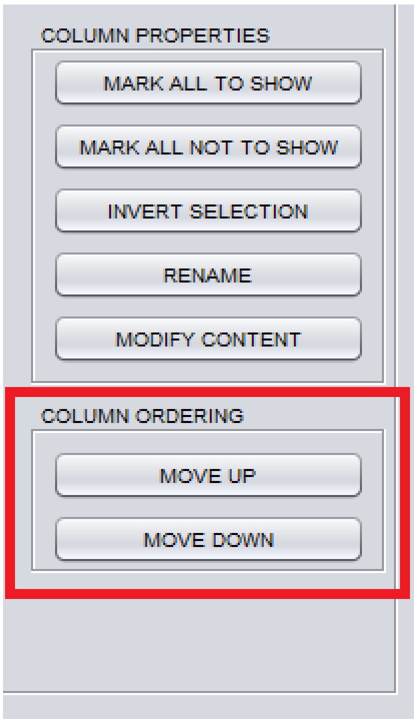

8.3.2 FILE SELECTION field

In this field it is possible to hide or unhide previously imported data set(s). As in case of column selection, click on a checkbox of matched file(s)or use **MARK ALL TO SHOW**, **MARK ALL NOT TO SHOW** and **INVERT SELECTION** buttons.

Hidden files are not deleted from the data set though, and are taken into account during every data processing step.

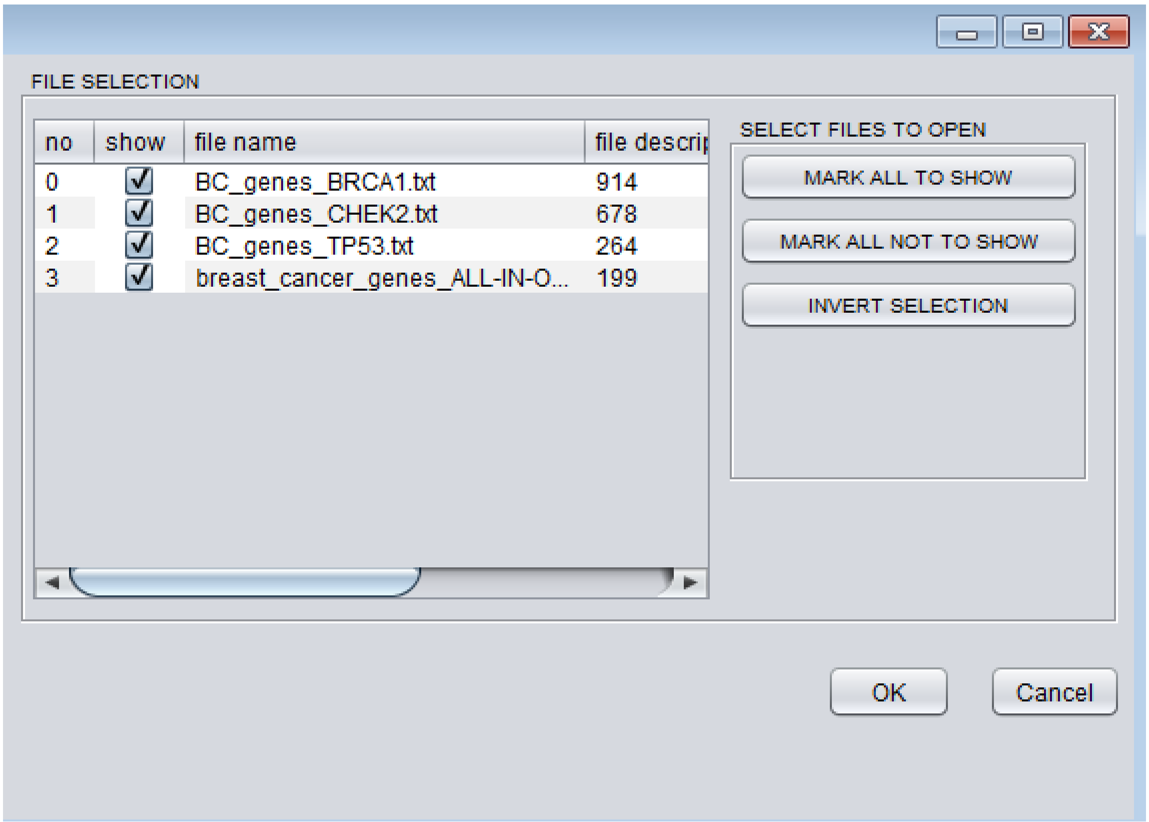

8.4 FILTERING THE INPUT FILE(S)

Advanced mode enables filtering which is carried out based on unlimited number of complex queries.

To set up filtering, click the **ADD** button in the query queue control section.

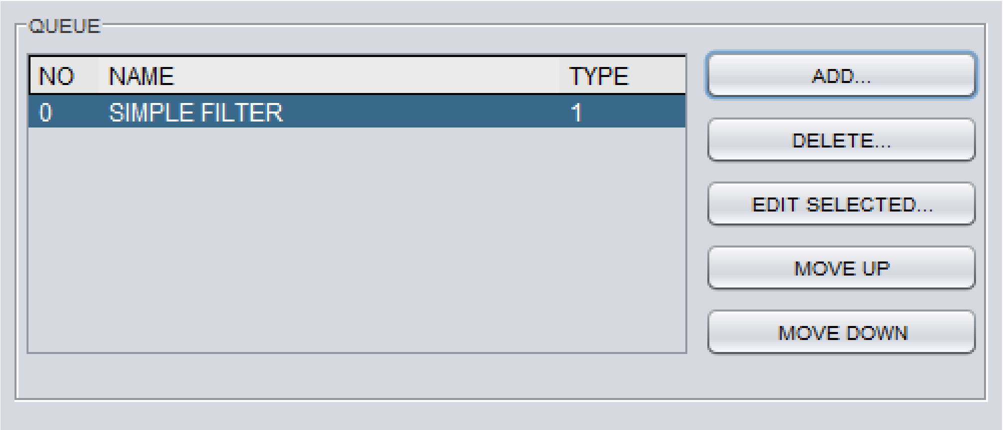

Subsequently, the **QUERY SETUP** window is opened that facilitates adding the queries.

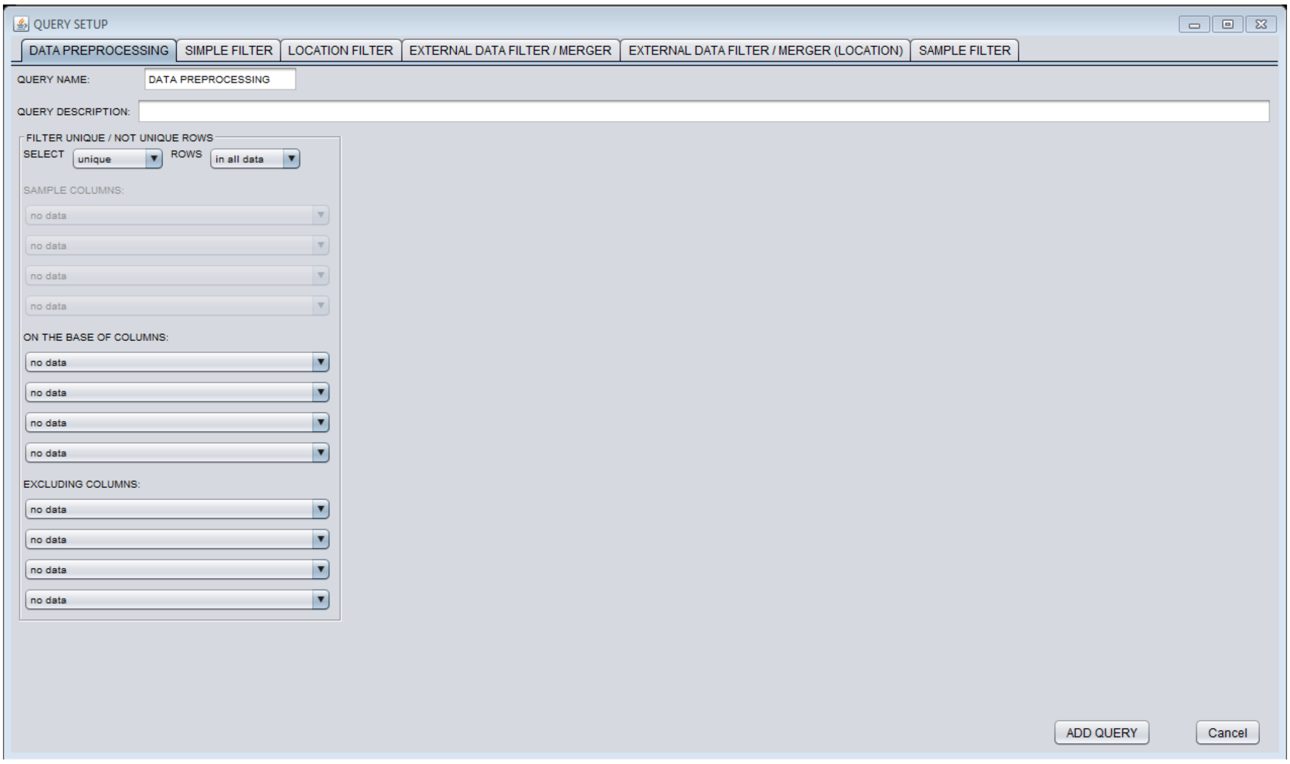

QUERY SETUP window has six tabs corresponding to different types of queries that can be handled by the program:

- **DATA PREPROCESSING**.
- **SIMPLE FILTER**.
- **LOCATION FILTER**.
- **EXTERNAL DATA FILTER / MERGER**.
- **EXTERNAL DATA FILTER / MERGER (LOCATION)**.
- **SAMPLE FILTER**.

Single query should only consist of parameters selected in one tab, as settings defined simultaneously in other tabs will not be executed. Consequently, the list of filtering criteria will be longer, but more legible.

8.4.1 DATA PREPROCESSING

DATA PREPROCESSING form is selected by default. This form is intended for global operations on the entire data sets.

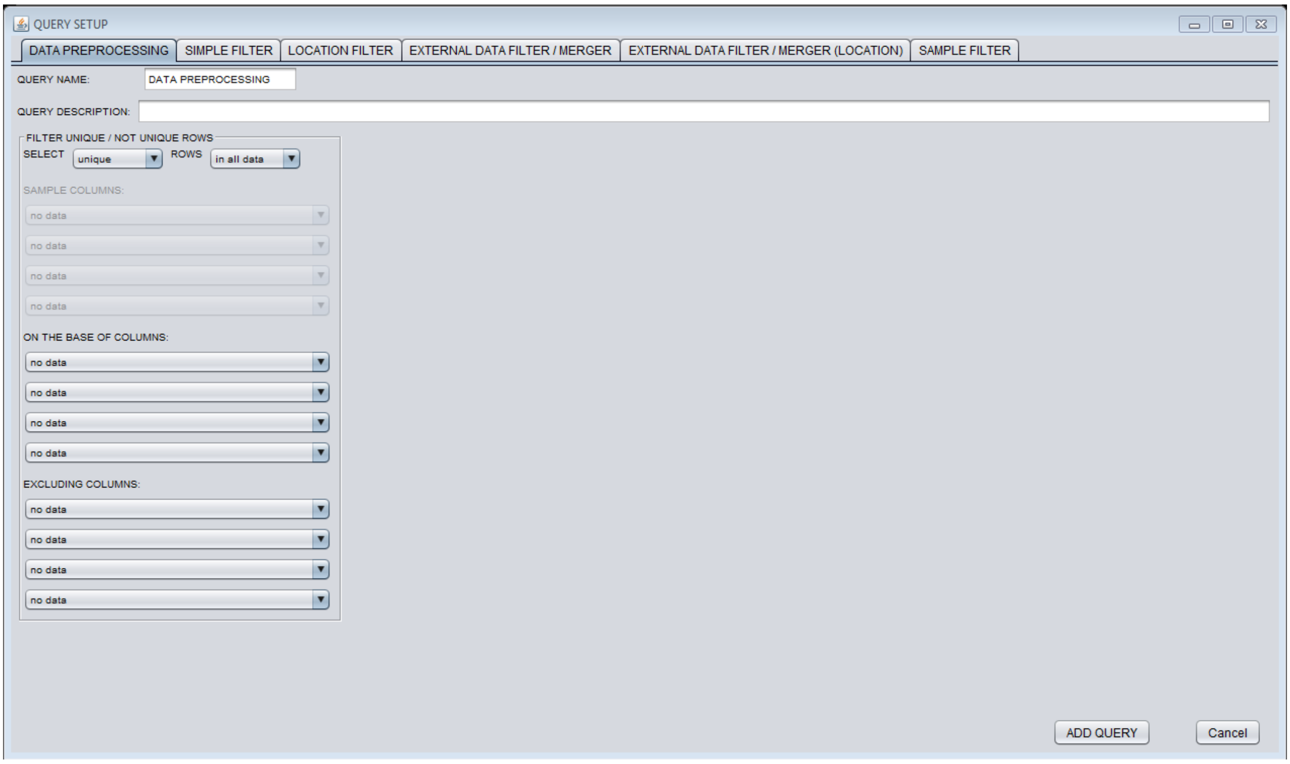

QUERY NAME and **QUERY DESCRIPTION** entry fields allow to assign the optional name and a short description to the query.

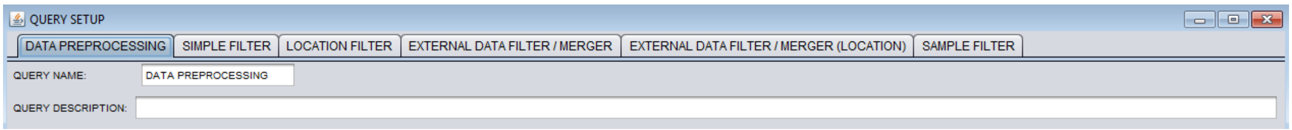

By using **SELECT** pull down menu only unique or redundant rows (occurring in the data more than once) can be retained.
The choice can be based on the unique/redundant rows in the entire data set, in each file separately or in each sample.

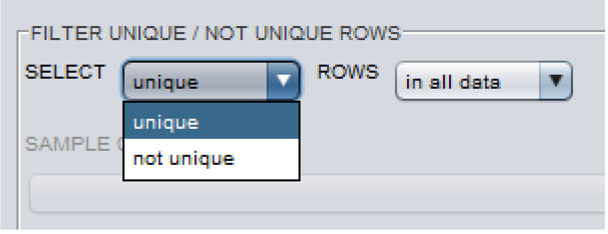

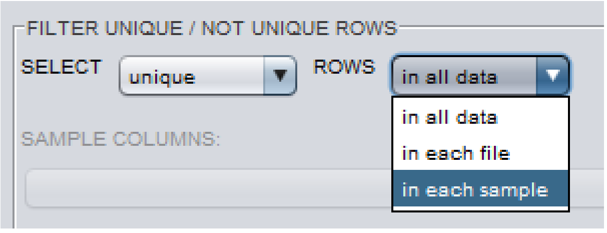

The uniqueness/redundancy can be determined based on the contents of the entire row (by default) or based on up to four indicated data column(s) (**ON THE BASE OF COLUMNS** subfield).

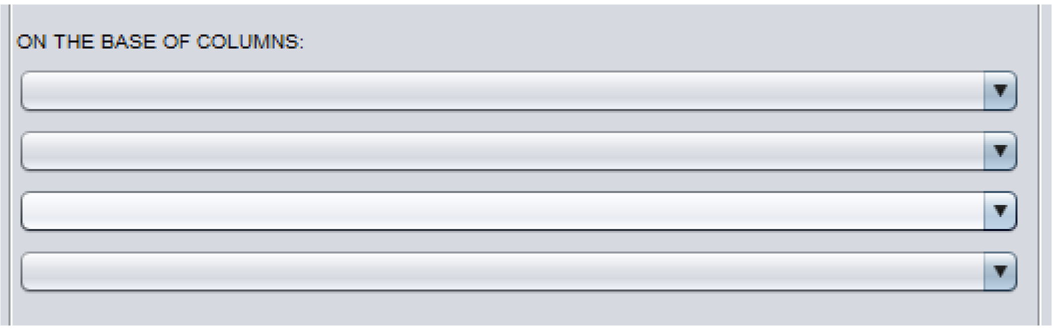

Alternatively, it is possible to determine the uniqueness/redundancy based on the contents of the entire row, but with the exclusion of up to four selected columns in the data (**EXCLUDING COLUMNS** subfield).

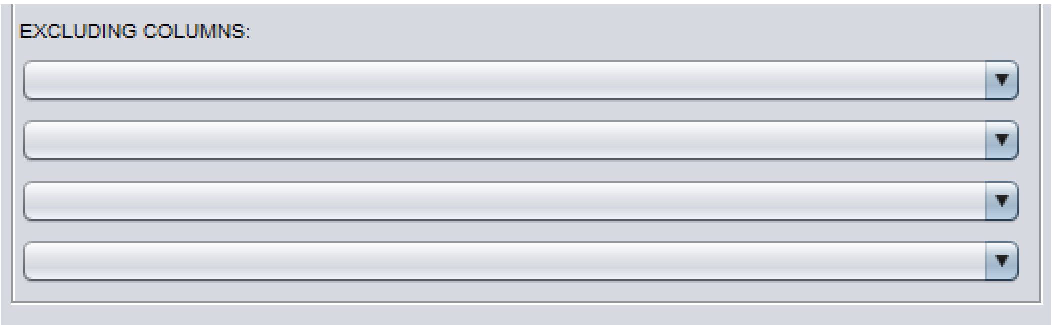

The described functions were used in example 1 in S2 Text. All needed example files were deposited on Open Science Framework (https://osf.io/pw2dx/) and are publicly available.

SIMPLE FILTER

SIMPLE FILTER refers to modifications and further filtering of the data stored in the indicated column.

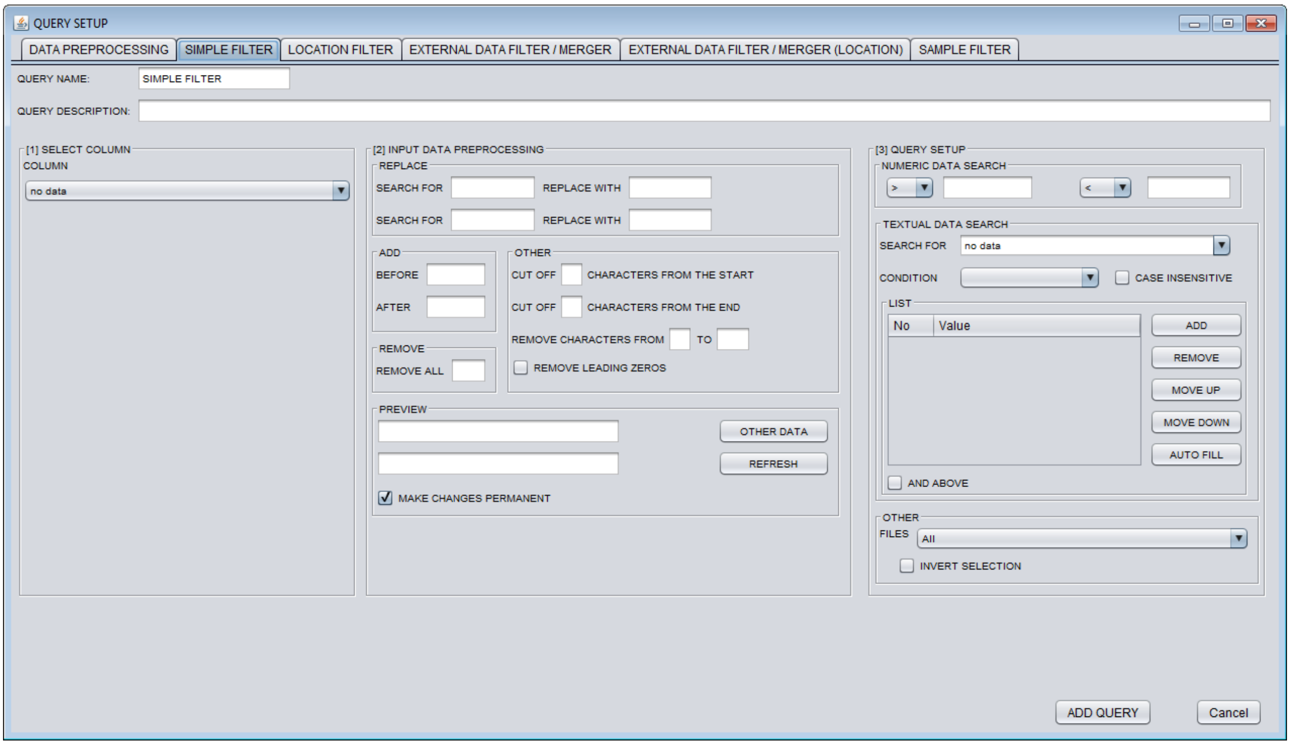

8.4.1.1 COLUMN SELECTION field

The first field, called **COLUMN SELECTION**, is used for choice of the column which will be subjected to filtering. Pull down menu located here contains the list of all columns included in the data set.

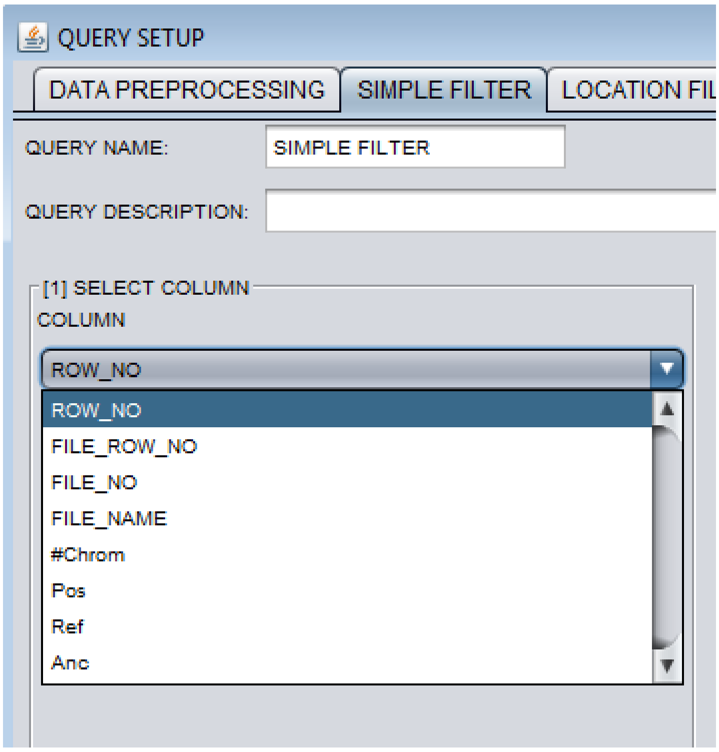

8.4.1.2 INPUT DATA PREPROCESSING field

In this field, data from the selected column can be prepared to the main filtering task.

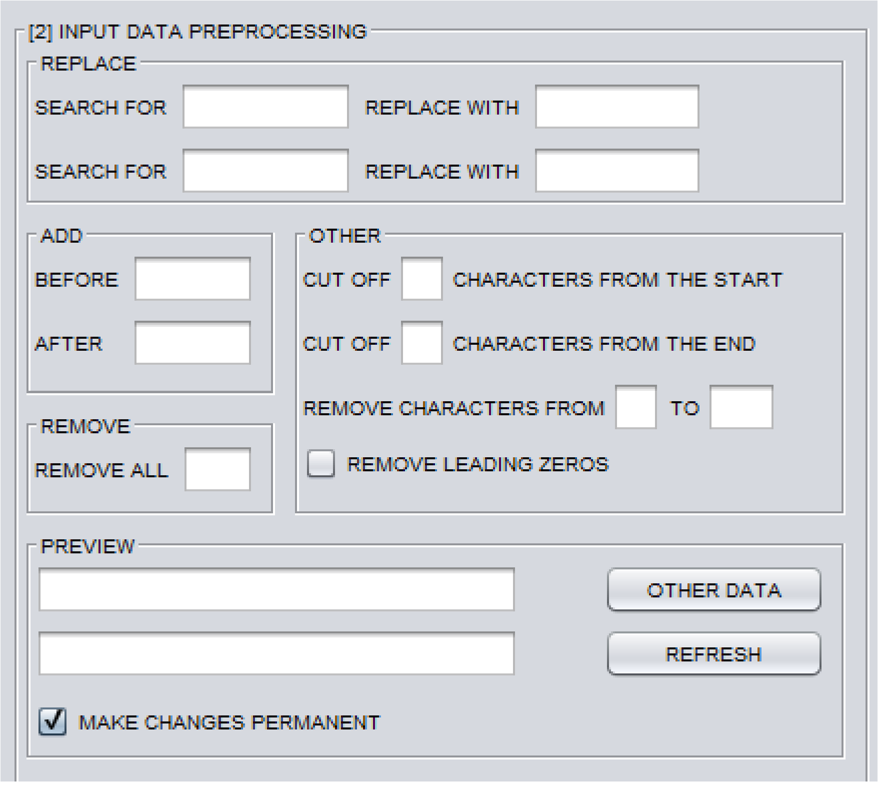

These preparatory operations include:

- Searching for alphanumeric strings to replace them with other phrases (**SEARCH FOR** … **REPLACE WITH** …).
-
- Adding alphanumeric strings before and/or after expressions in the rows (**ADD** – **BEFORE** … and **AFTER** …).
-
- Trimming the beginnings or ends of the alphanumeric strings (**CUT OFF** … **CHARACTERS FROM THE START** / **END**) or cutting the characters within the alphanumeric strings at the specified positions (**REMOVE CHARACTERS FROM** … **TO** …).
-
- Removing of so called “leading zeros”, eg. 000023 to 23 (**REMOVE LEADING ZEROS** checkbox).
-
- Removing specified expression from every data row (**REMOVE ALL…**).

The **PREVIEW** subfield shows a preview of introduced changes in the selected column and randomly selected row of data. The first line shows the original data and the second line presents the same data after processing.

The preview is updated upon introduction of any change in the **INPUT DATA PREPROCESSING** field, or can be enforced by clicking the **REFRESH** button. Also other exemplary rows of data can be drawn by clicking the **OTHER DATA** button.

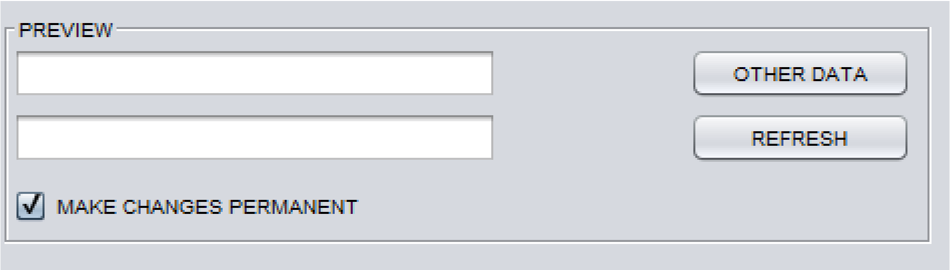

If the **MAKE CHANGES PERMANENT** box is not checked, the changes introduced in the **INPUT DATA PREPROCESSING** field have temporary character and facilitate customization of data for the **QUERY SETUP** field. In this case, after filtering has been applied, the actual data is not changed (the change is introduced temporarily then the original content is restored).

8.4.1.3 QUERY SETUP field

The query setup field serves for setting up the main filtering parameters. These parameters may refer to numeric or alphanumeric data, which are included in already chosen data column. Filtering can be done on data from one selected file from the dataset as well as on data from all loaded files.

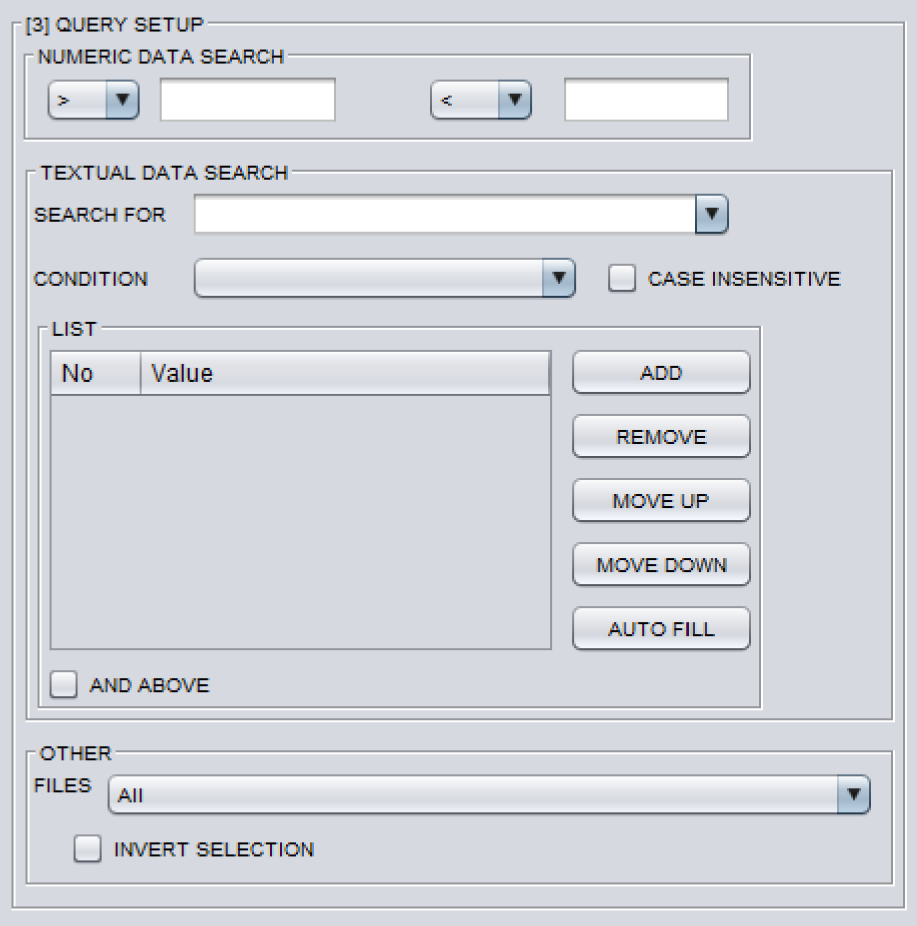

NUMERIC DATA SEARCH subfield provides searching for values less or greater than specified or values within the specified range.

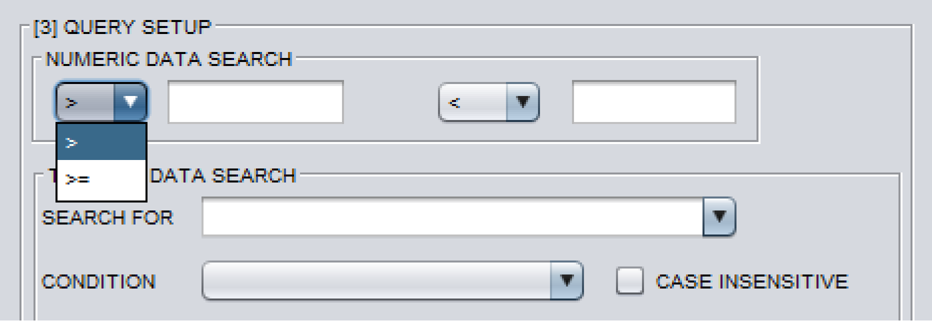

In the **TEXTUAL DATA SEARCH** subfield the data can be checked for matches with the specified alphanumeric substring.
Type the text to be searched or use the pull down list called **SEARCH FOR** to choose one of the expressions occurring in column. Furthermore, to specify the filtering task, following operators are available in the **CONDITION** pull down list: "**=**", "**<**", "**>**", "**<=**", "**>=**", "**<>**", "**Contains**", "**Does not contain**", "**Begins with**", "**Does not begin with**", "**Ends with**", "**Does not end with**".

Operations can be executed in case sensitive or insensitive way, respectively, which is defined using **CASE INSENSITIVE** checkbox.

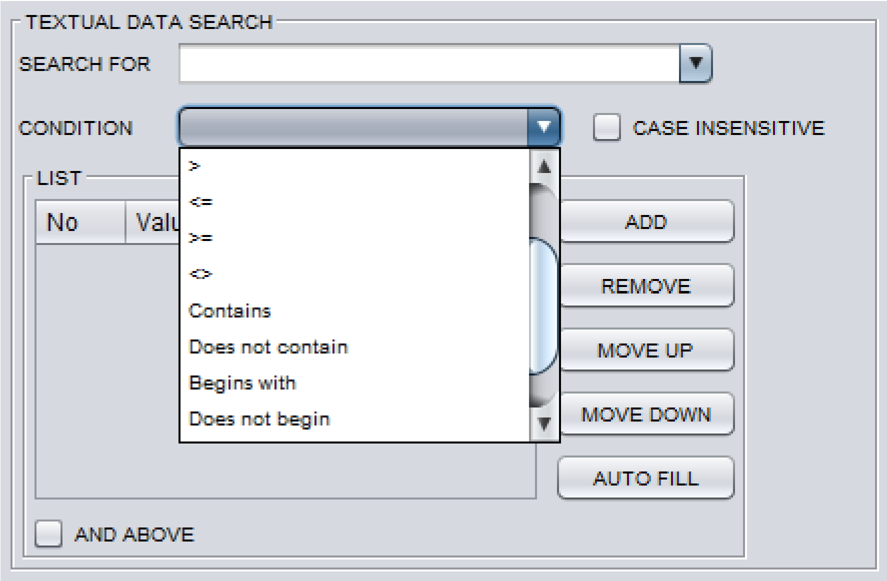

More complex filtering tasks can utilize the **LIST** feature. It allows to simultaneously look for more than one expression from the column selected in the first step of **SIMPLE FILTER**.

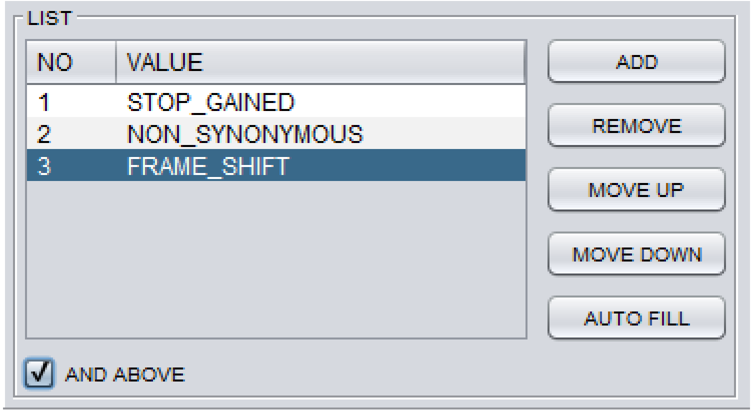

Each item in the list is added by clicking the **ADD** button. Subsequent items are shown denoted with a number (in the **NO** column) and a text to be searched (in the **VALUE** column).

To delete a particular item from the list click on it and then on the **REMOVE** button. An operation window will be displayed to confirm this action.

The order of listed expressions can be changed by using the **MOVE UP** and **MOVE DOWN** buttons. Select the item and then click on one of these two buttons. Every click changes the item's location by one position forward or backward.

The **AUTO FILL** function can be used to automatically create a list of all contents occurring in the indicated column.

The **LIST** can be utilized in 3 various ways:

- Without selecting any of the items in the list – in this case the contents of the selected column will be searched for the presence of either item in the list.
- With selecting an item in the list – in this case the contents of the selected column will be searched with regard to the conditions as defined by the selected item in the list.
- With selecting an item in the list and clicking the **AND ABOVE** checkbox – in this case the contents of selected column will be searched with regard to the conditions as defined by the selected item in the list as well as the items preceding the selected item in the list.

The described functions were used in example 2 (stage 7), 3 (stage 8) and 4 (stage 2 and 3) in S2 Text. All needed example files were deposited on Open Science Framework (https://osf.io/pw2dx/) and are publicly available.

8.4.2 LOCATION FILTER

LOCATION FILTER is useful to search for a data on the basis of the locus.

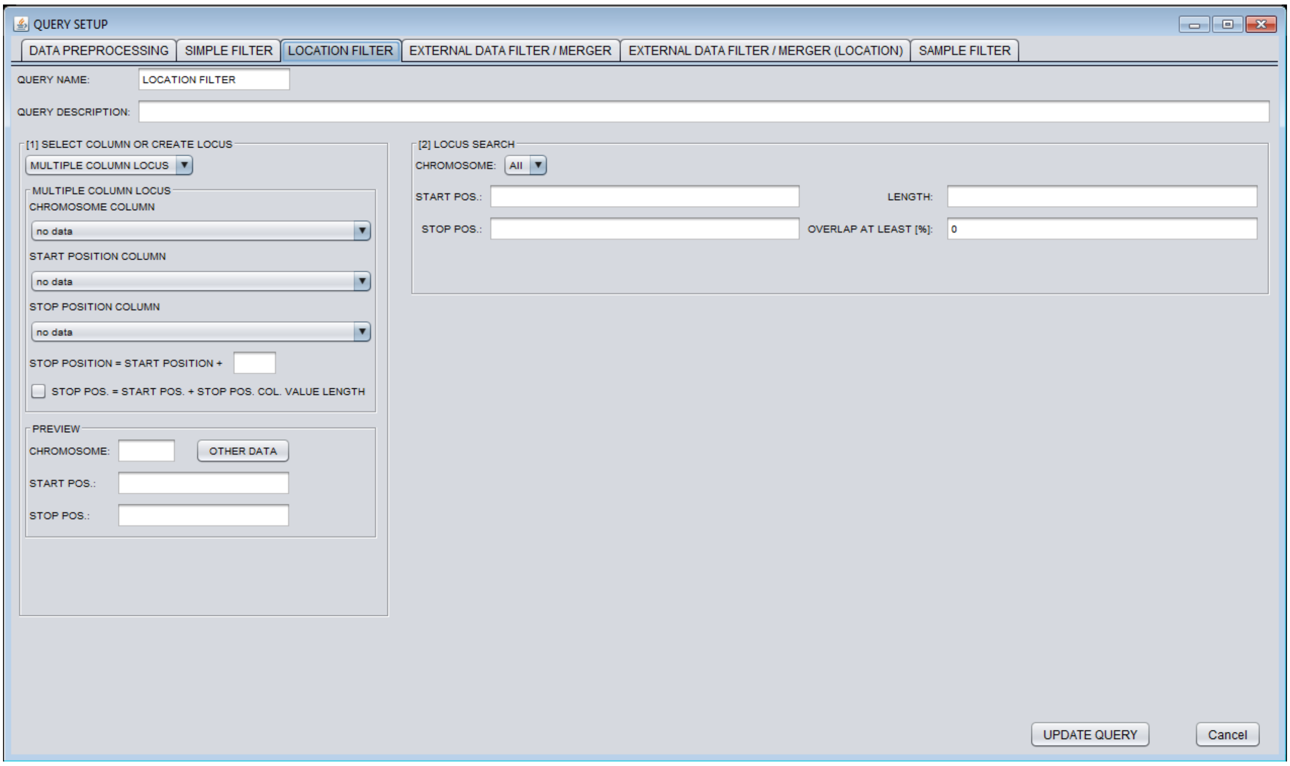

8.4.2.1 SELECT COLUMN OR CREATE A LOCUS field

The first field, called **SELECT COLUMN OR CREATE A LOCUS**, is designed to define the format of location data. It can be done in two ways – there are two options in the pull down menu:

- Using **MULTIPLE COLUMN LOCUS** – indicate which columns (presented in the pull down lists) refer to the number of the chromosome (**CHROMOSOME COLUMN**), start position (**STARTING POSITION COLUMN**) and stop position (**STOP POSITION COLUMN**), respectively.

Alternatively, the stop position can be created by adding any number to the start position. Therefore all loci will have the same length (**STOP POSITION = START POSITION + …**).

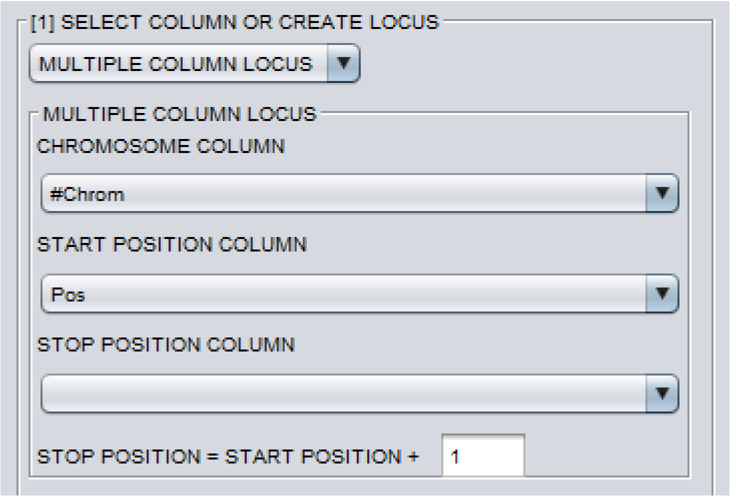

- Using **SINGLE COLUMN LOCUS** – indicate, which column contains complete information about the locus and then choose its format from the pull down list (**LOCUS COLUMN FORMAT**). Four different formats are available:

✓ Chromosome: start – stop.
✓ Chromosome start stop.
✓ Chromosome – start – stop.
✓ Chromosome: start : stop.

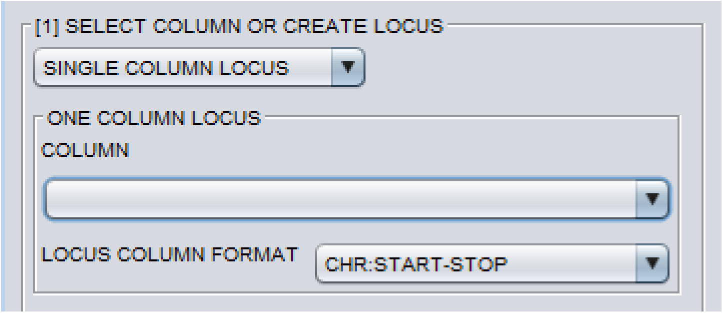

PREVIEW subfield, located below, displays the preview of locus data based on the one randomly chosen row. The other exemplary rows can be drawn by clicking the **OTHER DATA** button.

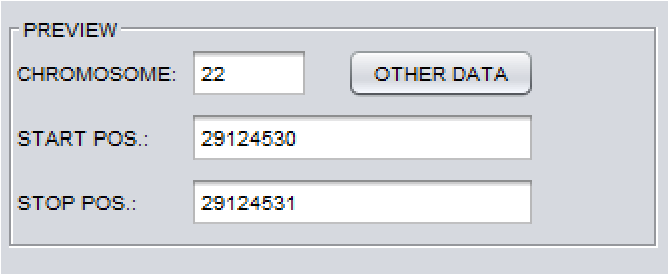

8.4.2.2 LOCUS SEARCH field

LOCUS SEARCH is created to select the rows containing particular locus data. The search can be done with regard to one chromosome or all chromosomes present in the data – to determine it, there is a **CHROMOSOME** pull down list.
This option may be used in 3 main ways:

- To look for a specified locus, the **START POS.** and **STOP POS.** entry fields must be filled.
- To look for a locus of defined length, the **START POS.** must be completed as well as the number of nucleotides in the **LENGTH** entry field.
- To look for a particular percentage overlap with the specified locus, the **START POS.** and **STOP POS.** entry fields must be filled along with the **OVERLAP [%]** entry field.

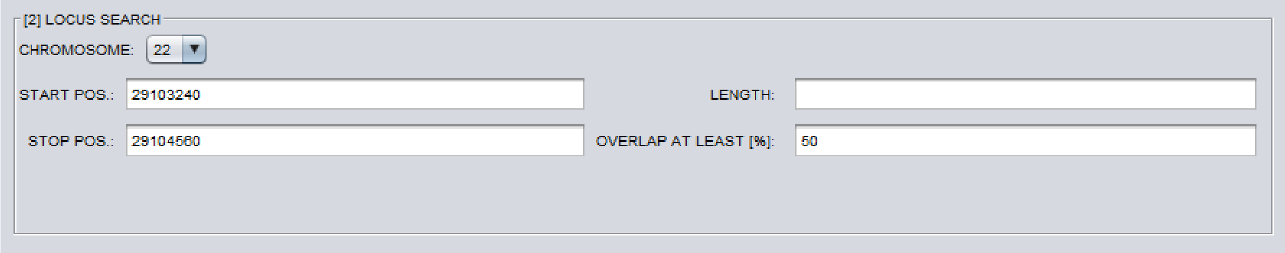

The described functions were used in example 1, 4 in S2 Text. All needed example files were deposited on Open Science Framework (https://osf.io/pw2dx/) and are publicly available. EXTERNAL DATA FILTER/MERGER

EXTERNAL DATA FILTER/MERGER form enables two main actions:

- Filtering the main file, namely particular column(s), on the basis of up to four columns from the additional file.
- Merging the matching data from external file(s) with the main file.

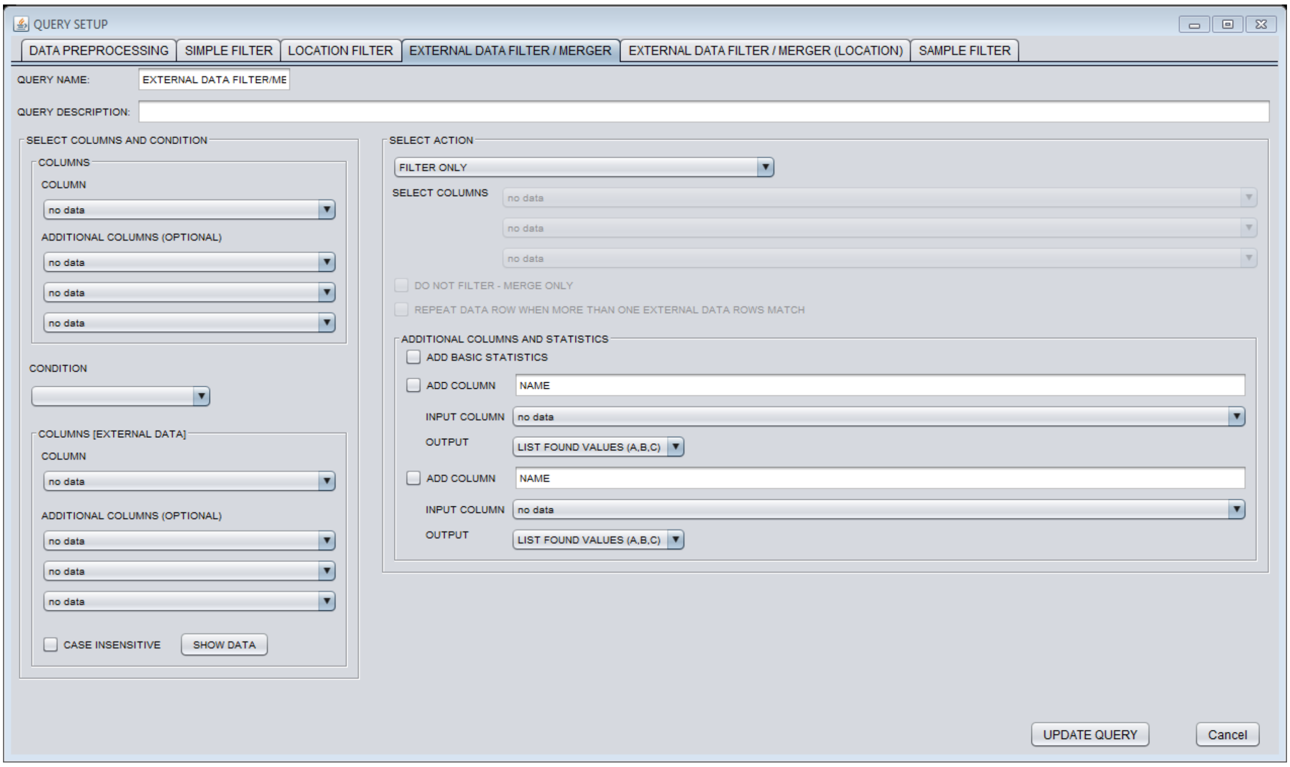
QUERY SETUP
DATA PREPROCESSING
SIMPLE FILTER
LOCATION FILTER
EXTERNAL DATA FILTER / MERGER
EXTERNAL DATA FILTER / MERGER (LOCATION)
SAMPLE FILTER
QUERY NAME:
EXTERNAL DATA FILTER/ME
QUERY DESCRIPTION:
SELECT COLUMNS AND CONDITION
COLUMNS
COLUMN
no data
ADDITIONAL COLUMNS (OPTIONAL)
no data
no data
no data
CONDITION
COLUMNS [EXTERNAL DATA]
COLUMN
no data
ADDITIONAL COLUMNS (OPTIONAL)
no data
no data
no data
CASE INSENSITIVE
SHOW DATA
SELECT ACTION
FILTER ONLY
SELECT COLUMNS
no data
no data
no data
DO NOT FILTER - MERGE ONLY
REPEAT DATA ROW WHEN MORE THAN ONE EXTERNAL DATA ROWS MATCH
ADDITIONAL COLUMNS AND STATISTICS
ADD BASIC STATISTICS
ADD COLUMN
NAME
INPUT COLUMN
no data
OUTPUT
LIST FOUND VALUES (A,B,C)
ADD COLUMN
NAME
INPUT COLUMN
no data
OUTPUT
LIST FOUND VALUES (A,B,C)
UPDATE QUERY
Cancel

8.4.2.3 SELECT COLUMNS AND CONDITION field

Here, a set of columns from the main file and external data is chosen to define the filtering/merging criteria.

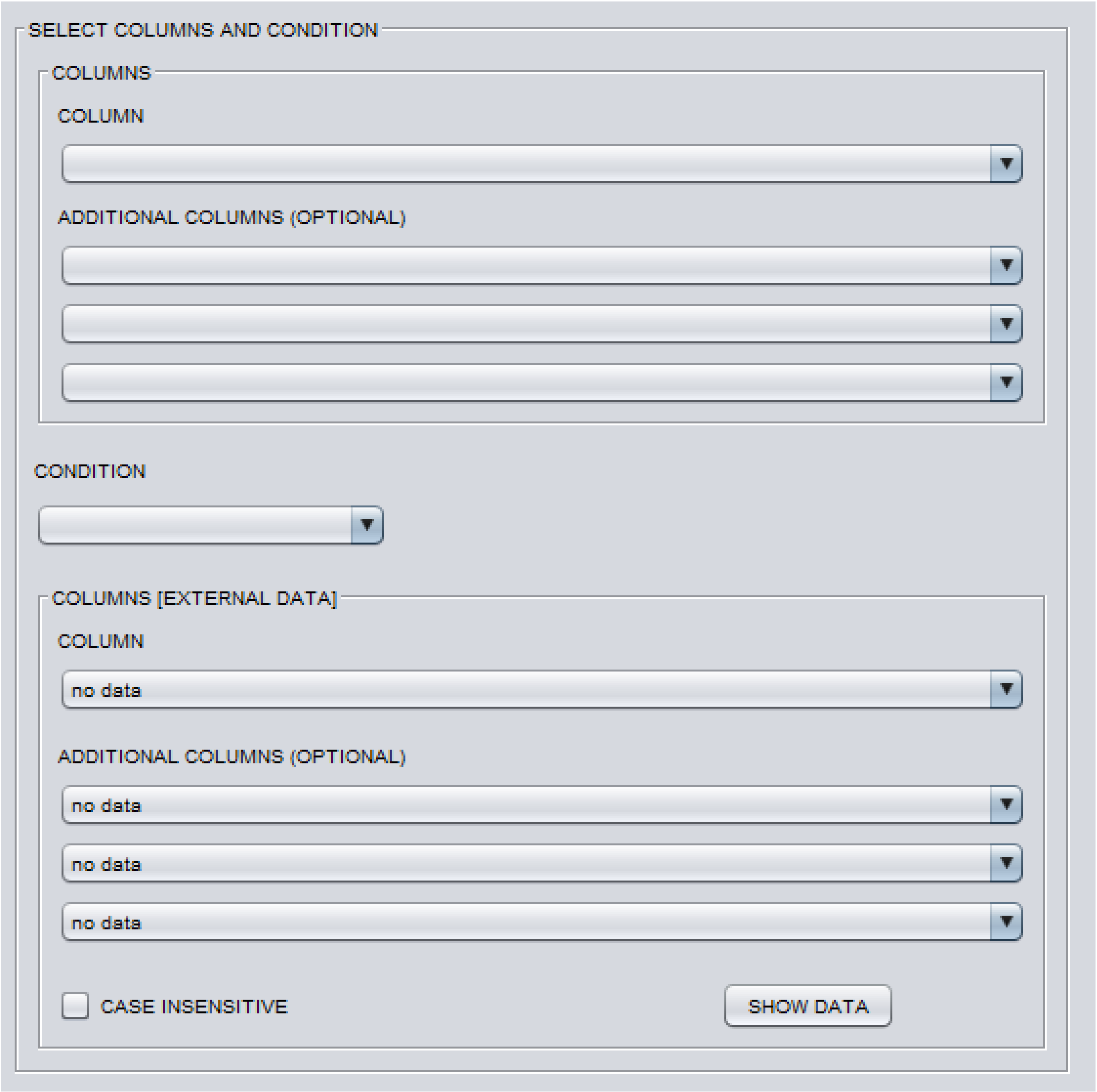

In the first step choose from one up to four columns from the main file ((**COLUMN**) and **ADDITIONAL COLUMNS (OPTIONAL)** from the **COLUMNS** subfield). An input dataset, which is created in this way, will be subjected to processing.

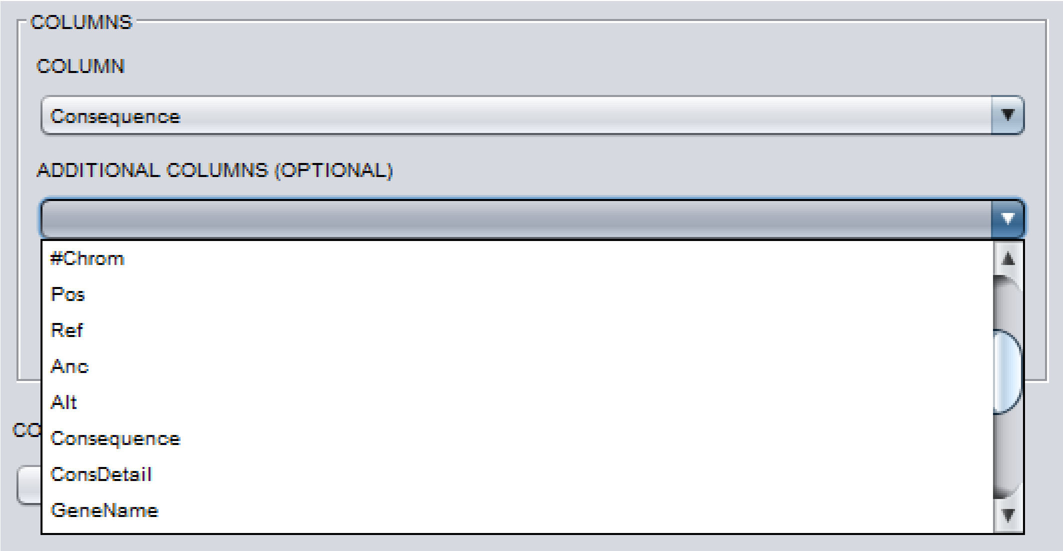

Using the pull down list called **CONDITION**, define the condition with regard to the filtering or merging task. There are 12 following options here, including simple mathematical signs and expressions – "=", "<", ">", "=<", "=>", "< >", "**Contains**", "**Does not contain**", "**Begins with**", "**Does not begin with**", "**Ends with**" and "**Does not end with**".

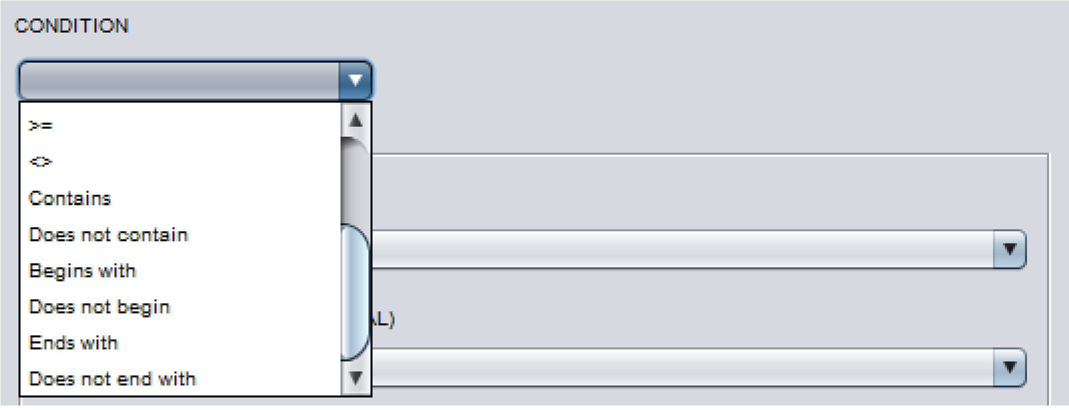

Finally, select from one up to four columns from external data file(s) (**COLUMN (EXTERNAL DATA)** and **ADDITIONAL COLUMNS (OPTIONAL)** which are located in the **COLUMNS [EXTERNAL FILE]** subfield). To facilitate the choice of columns, especially from several files, the **SHOW DATA** button is available to display the preview of the external dataset.

Moreover, filtering or merging on the basis of selected columns may be done in case insensitive way (**CASE INSENSITIVE** checkbox).

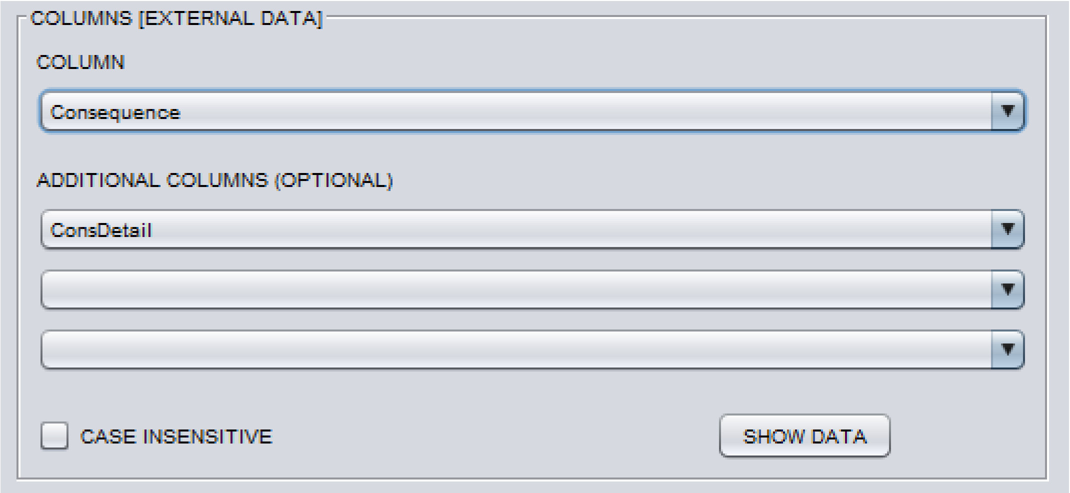

8.4.2.4 SELECT ACTION field

The **SELECT ACTION** is intended to determine the data processing operation.

The major step is to select one of three following alternatives:

- **FILTER ONLY** – data from the main file will be filtered on the basis of previously chosen condition and set of columns from the external file(s).

- **ADD ALL COLUMNS FROM EXTERNAL DATA MATCHING ROWS** – all previously selected columns that contain information matching defined criteria will be appended to the input dataset.

- **ADD SELECTED COLUMNS FROM EXTERNAL DATA MATCHING ROWS** – only selected columns from matching rows will be appended to the input dataset. Here, the **SELECT COLUMNS** subfield is active to choose up to three columns using pull down lists.

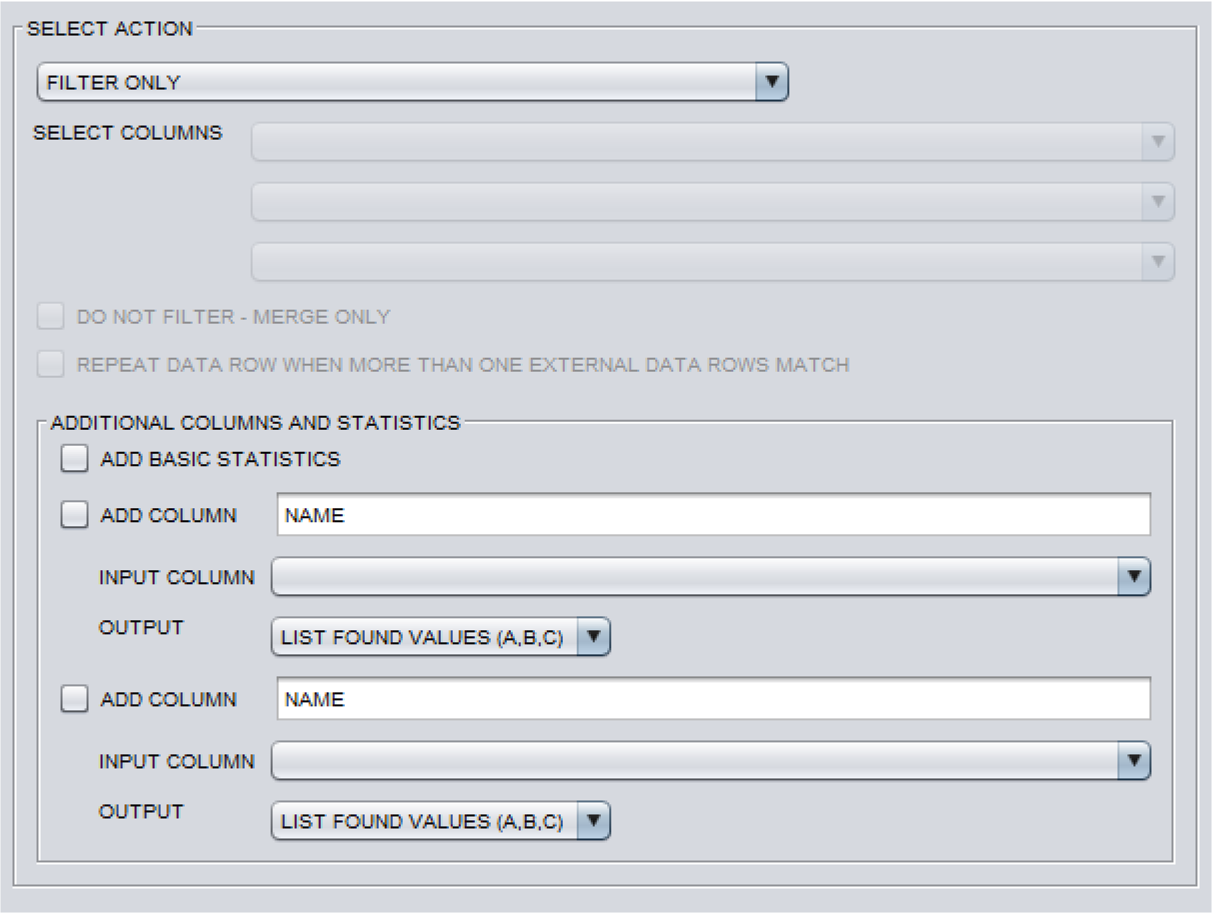

While merging files together, all rows from the main file that do not have matching data in the external file, will be filtered out and thus not visible. To keep all rows from the main file the **DO NOT FILTER – MERGE ONLY** option is useful. If the checkbox is marked, to every

row from the main file which does not have any matching data in the external file, empty fields will be inserted in places corresponding to columns from the external file. This option is available for both adding actions – **ADD ALL COLUMNS FROM EXTERNAL DATA MATCHING ROWS** and **ADD SELECTED COLUMNS FROM EXTERNAL DATA MATCHING ROWS**.

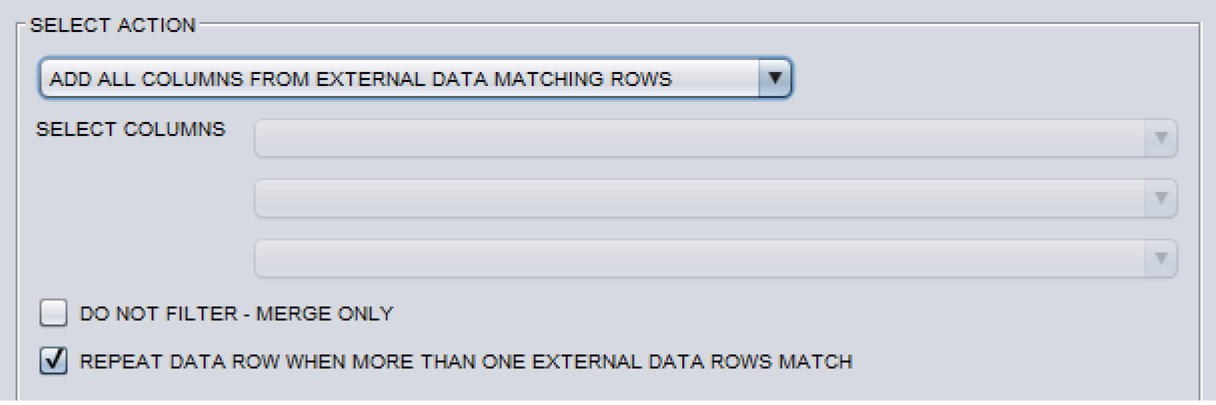

REPEAT DATA WHEN MORE THAN ONE EXTERNAL DATA ROWS MATCH checkbox is automatically marked while selecting the merging action. This option is designed to highlight the repeated occurrence of the same data row in the external file(s) – when two or more matching data rows (based on the condition and set of columns) are found in external dataset they will be repeated also after combining the data.
If this option is inactive, only the first occurrence of matching data in external file, will be shown.

8.4.2.5 ADDITIONAL COLUMNS AND STATISTICS subfield

This subfield facilitates adding further information to the newly created in this form dataset.

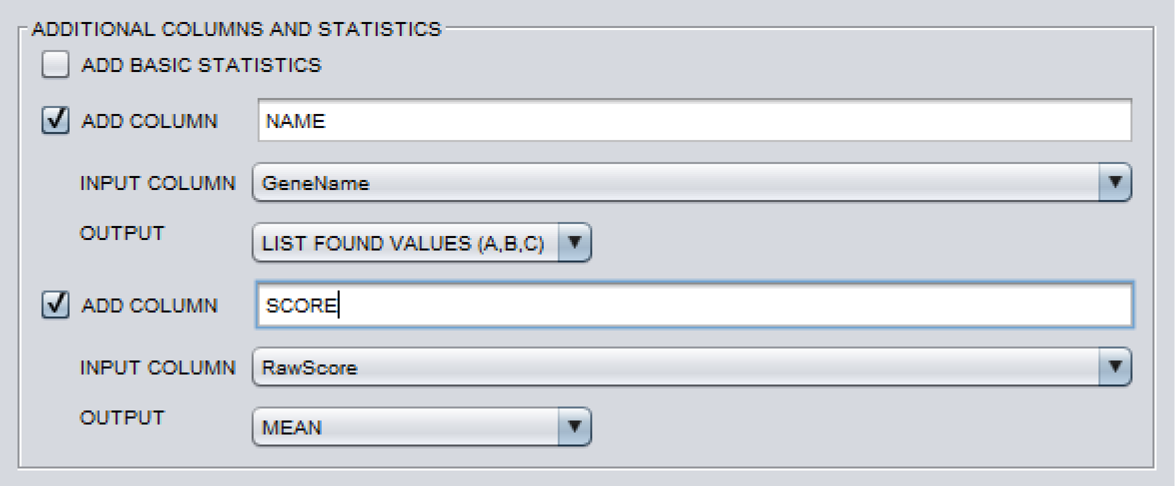

The **ADD COLUMN** checkbox enables appending a single column from external file to existing dataset. Any column, containing the data of interest, can be chosen, even that is not directly related to the main column(s) from the **SELECT COLUMNS AND CONDITION** field.

In the **NAME** entry field the title of the new column has to be entered. Then, from the **INPUT COLUMN** pull down list, one column from the external file is selected. Finally, the **OUTPUT** pull down list specifies the way of presenting the data from matching rows. There are four different possibilities:

- **LIST FOUND VALUES (A,B,C)** – if there is more than one matching result, all of them will be listed, separated with commas.
- **LIST FOUND VALUES (A;B;C)** – all of matching results will be listed, separated with semicolons.
- **MEAN** – the mean value of matching results will be calculated and presented if the data format is appropriate.
- **MEDIAN** – the median value of matching results will be calculated and presented if the data format is appropriate.

The described functions were used in example 1 and 2 (stage 9) in S2 Text. All needed example files were deposited on Open Science Framework (https://osf.io/pw2dx/) and are publicly available. EXTERNAL DATA FILTER/MERGER (LOCATION)

EXTERNAL DATA FILTER/MERGER (LOCATION) form is very similar to the **EXTERNAL DATA FILTER/MERGER** as it also enables two main actions, but they are performed only taking the locus data into account:

- Filtering the main file, namely particular column(s), on the basis of up to four columns from the additional file.
- Merging the matching data from external file(s) with the main file.

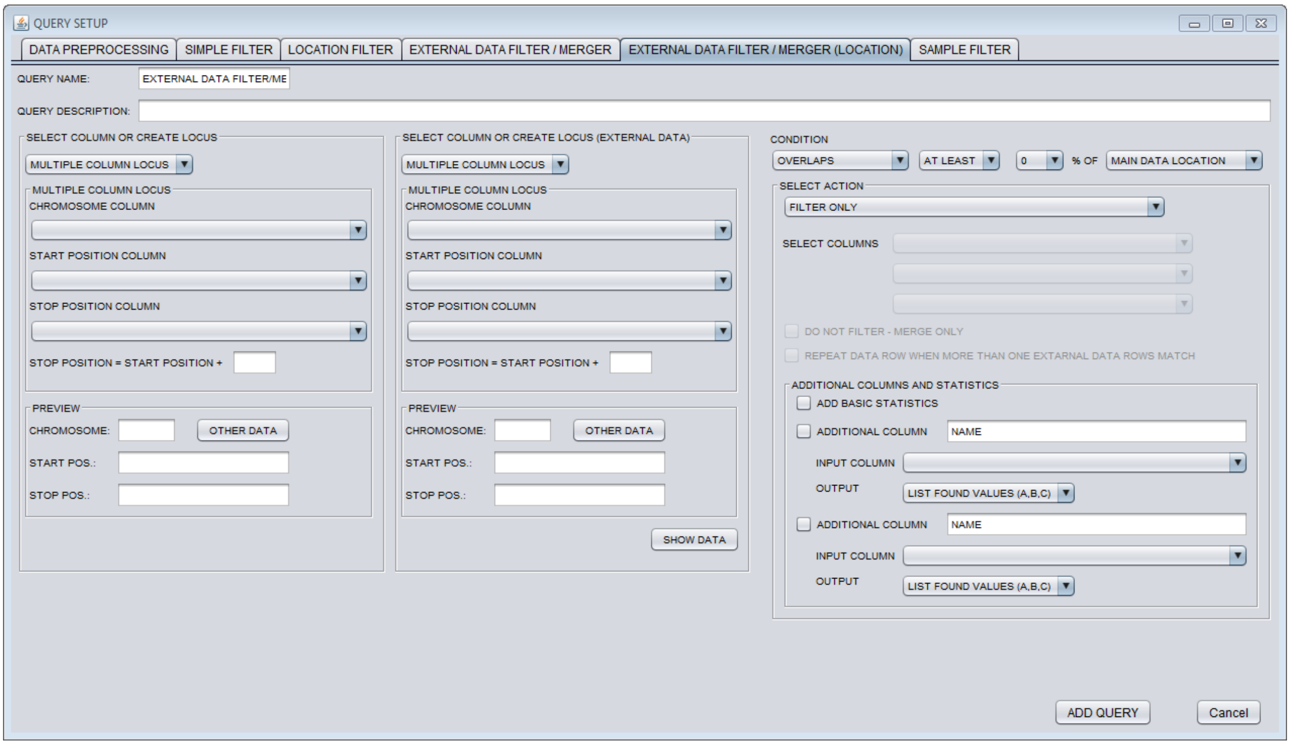

8.4.2.6 SELECT COLUMN OR CREATE LOCUS field

This field works in the same way as in **LOCATION FILTER** form. It is created to set the format of location data using one of the two ways available in the pull down menu:

- Using **MULTIPLE COLUMN LOCUS** – indicate which columns (presented in the pull down lists) refer to the number of the chromosome (**CHROMOSOME COLUMN**), start position (**STARTING POSITION COLUMN**) and stop position (**STOP POSITION COLUMN**), respectively.

Alternatively, the stop position can be created by adding any number to the start position. Therefore all loci will have the same length (**STOP POSITION = START POSITION + …**).

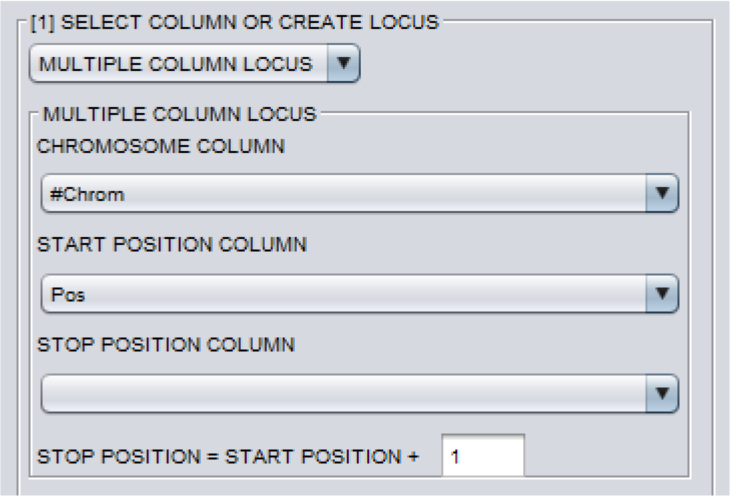

- Using **SINGLE COLUMN LOCUS** – indicate, which column contains complete information about the locus and then choose its format from the pull down list (**LOCUS COLUMN FORMAT**). Four different formats are available:

✓ Chromosome: start – stop.
✓ Chromosome start stop.
✓ Chromosome – start – stop.
✓ Chromosome: start : stop.

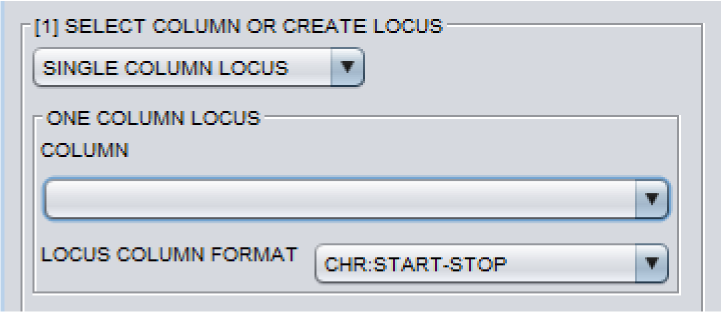

PREVIEW subfield, located below, displays the preview of locus data based on the one randomly chosen row. The other exemplary rows can be drawn by clicking the **OTHER DATA** button.

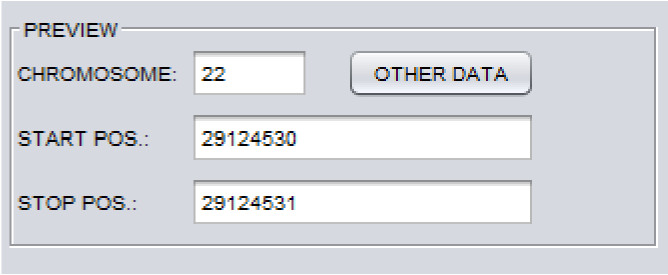

8.4.2.7 SELECT COLUMN OR CREATE LOCUS (EXTERNAL DATA) field

This field refers to setting the format of location data in the external data file(s). All options operate as in the previous field (**SELECT COLUMN OR CREATE LOCUS**).

The additional button, called **SHOW DATA**, displays the preview of the external dataset to facilitate the choice of proper columns.

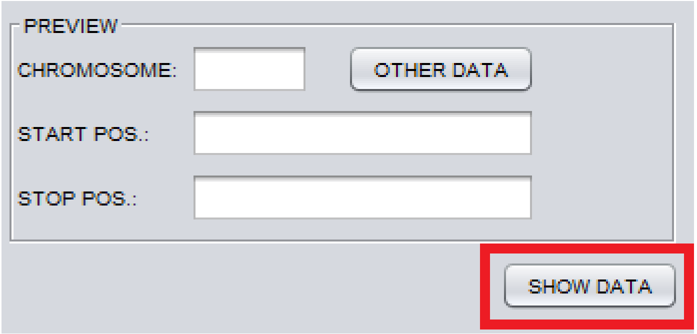

8.4.2.8 CONDITION field

CONDITION field consists of four pull down lists to determine subsequent processing criteria.

The first one is used to choose if the main file will be analyzed for overlapping or containing certain location data as there are four options – **OVERLAPS**, **DOES NOT OVERLAP**, **CONTAINS**, **DOES NOT CONTAIN**.
Containing refers to fully lying inside the particular location and overlapping to lying even partially.

The second pull down list refers to the scope of the overlap or containing – **AT LEAST** and **AT MOST** are the alternatives here.

The third one enables selection of the percentage value from 0 to 100 for **AT LEAST** or **AT MOST** option.

The last pull down menu is to choose the objective of the condition – location data from the main file (**MAIN DATA LOCATION**), from the external file (**EXTERNAL DATA LOCATION**) or including both of these datasets (**BOTH**). **BOTH** option is built up of two loops of analysis – the first one filters the main file and then the second one searches for matching data among them.

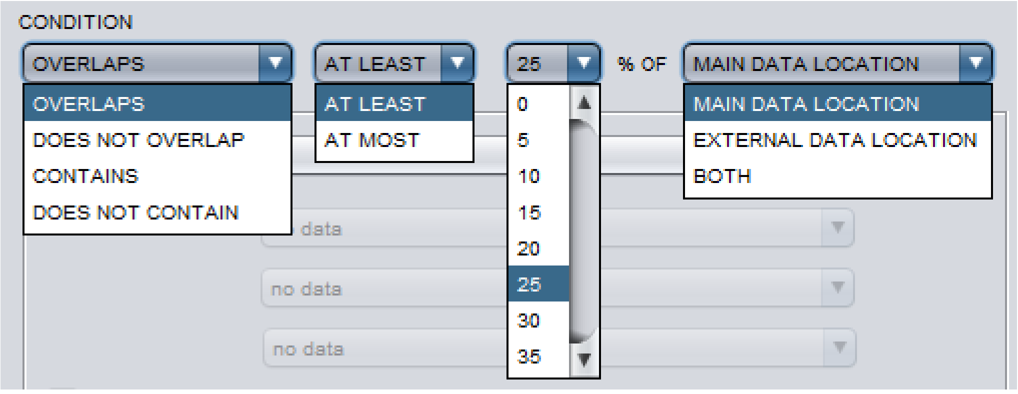

8.4.2.9 SELECT ACTION field

The **SELECT ACTION** subfield is identical with that present in **EXTERNAL DATA FILTER/MERGER** form and is intended to determine the data processing operation. The major step is to select one of three following alternatives:

- **FILTER ONLY** – data from the main file will be filtered on the basis of previously chosen condition and set of columns from the external file(s).
- **ADD ALL COLUMNS FROM EXTERNAL DATA MATCHING ROWS** – all previously selected columns that contain information matching defined criteria will be appended to the input dataset.
- **ADD SELECTED COLUMNS FROM EXTERNAL DATA MATCHING ROWS** – only selected columns from matching rows will be appended to the input dataset. Here, the **SELECT COLUMNS** subfield is active to choose up to three columns using pull down lists.

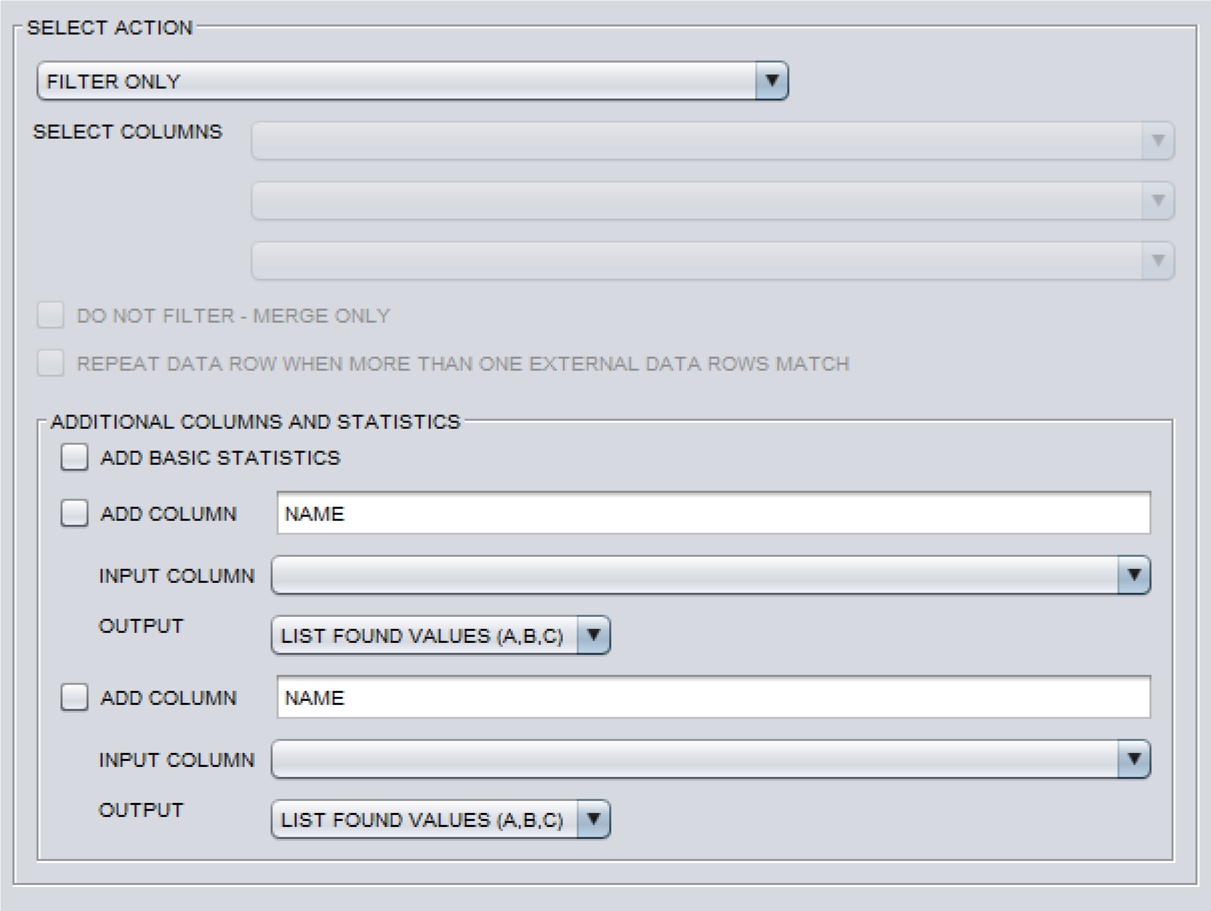

While merging files together, all rows from the main file that do not have matching data in the external file, will be filtered out and thus not visible. To keep all rows from the main file the **DO NOT FILTER – MERGE ONLY** option is useful. If the checkbox is marked, to every row from the main file which does not have any matching data in the external file, empty fields will be inserted in places corresponding to columns from the external file. This option is available for both adding actions – **ADD ALL COLUMNS FROM EXTERNAL DATA MATCHING ROWS** and **ADD SELECTED COLUMNS FROM EXTERNAL DATA MATCHING ROWS**.

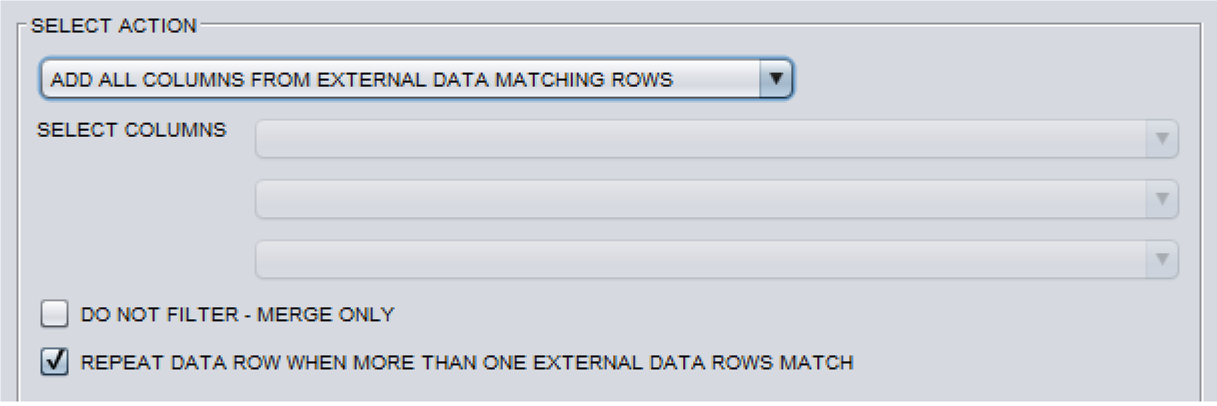

REPEAT DATA WHEN MORE THAN ONE EXTERNAL DATA ROWS MATCH checkbox is automatically marked while selecting the merging action. This option is designed to highlight the repeated occurrence of the same data row in the external file(s) – when two or more matching data rows (based on the condition and set of columns defining a locus) are found in external dataset they will be repeated also after combining the data.
If this option is inactive, only the first occurrence of matching data in external file, will be shown.

8.4.2.10 ADDITIONAL COLUMNS AND STATISTICS subfield

ADDITIONAL COLUMNS AND STATISTICS subfield facilitates adding further information to the newly created in this form dataset.

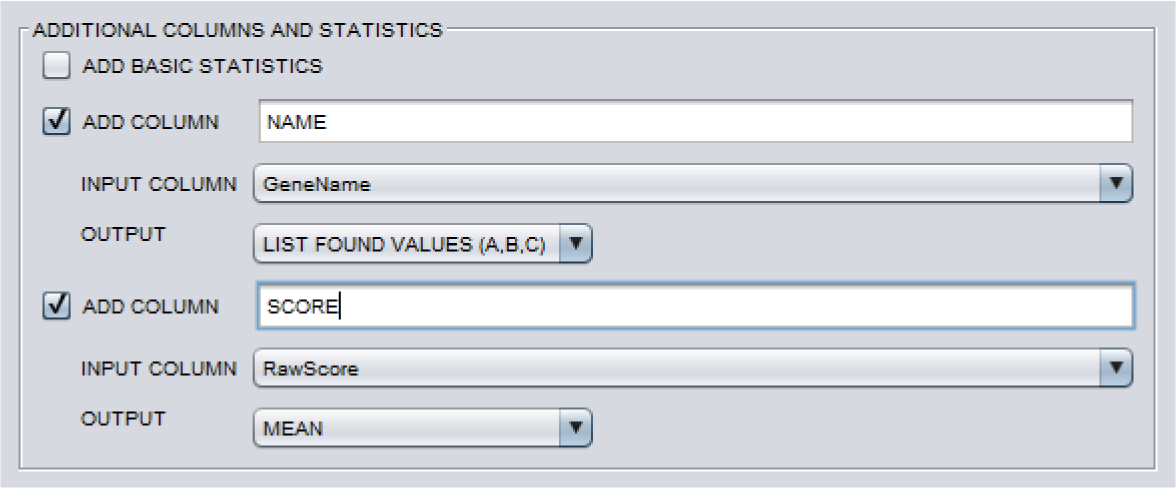

If **ADD BASIC STATISTICS** checkbox is active, an extra column will be added to the dataset – containing the overall number of external data rows that matches the main file based on the chosen column(s) and condition.

The **ADD COLUMN** checkbox enables appending a single column from external file to existing dataset. Any column, containing the data of interest, can be chosen, even that is not directly related to the main column(s) from the **SELECT COLUMNS AND CONDITION** field.

In the **NAME** entry field the title of the new column has to be entered. Then, from the **INPUT COLUMN** pull down list, one column from the external file is selected. Finally, the **OUTPUT** pull down list specifies the way of presenting the data from matching rows. There are four different possibilities:

- **LIST FOUND VALUES (A,B,C)** – if there is more than one matching result, all of them will be listed, separated with commas.

- **LIST FOUND VALUES (A;B;C)** – all of matching results will be listed, separated with semicolons.
- **MEAN** – the mean value of matching results will be calculated and presented if the data format is appropriate.
- **MEDIAN** – the median value of matching results will be calculated and presented if the data format is appropriate.

The described functions were used in example 1, 2 (stage 2), 3 (stage 3,6 and 7) and 4 (stage 4) in S2 Text. All needed example files were deposited on Open Science Framework (https://osf.io/pw2dx/) and are publicly available. SAMPLE FILTER

The last form from the **QUERY SETUP** window is designed for filtering with regard to differences in samples.

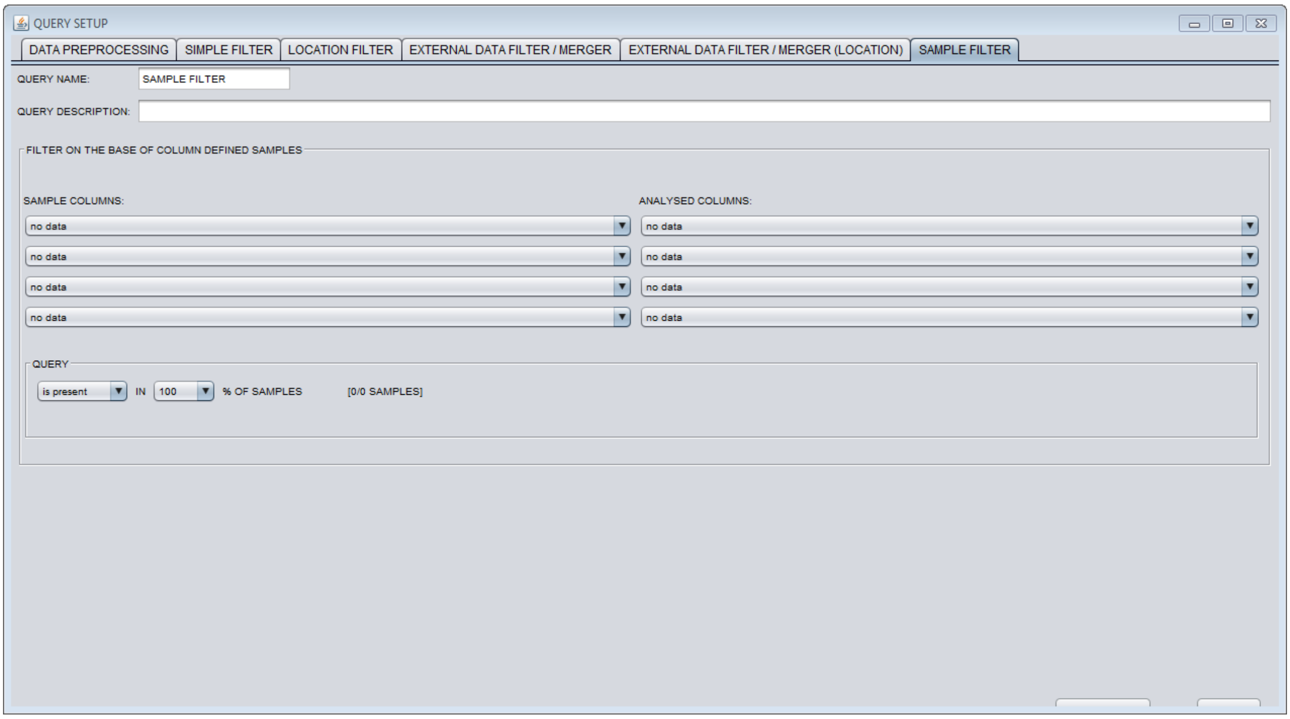

Here, sample is an individual object that can be defined by up to four columns from **SAMPLE COLUMNS** pull down lists. Columns, that will be examined, have to be chosen using **ANALYSED COLUMNS** pull down lists. Also, the maximal number of columns is four.

In order to specify the query, the **QUERY** subfield is located below. The first pull down list is intended for the selection of the condition – **is present**, **is not present**, **select largest**, **select smallest**. In case of the presence/absence condition – the second pull down list is to choose the percentage of samples (**from 1 to 100%**) for it.

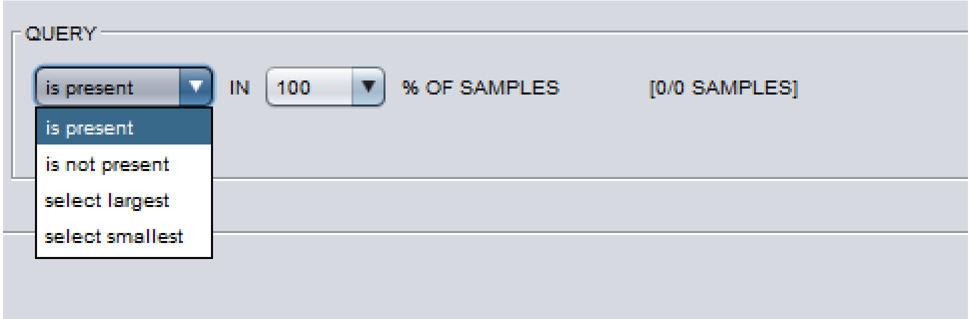

The described functions were used in example 1 in S2 Text. All needed example files were deposited on Open Science Framework (https://osf.io/pw2dx/) and are publicly available.

8.5 GENERATING REPORT

After setting the filtering parameters in the **QUEUE** field, the analysis will be carried out with the click on **GENERATE REPORT** button from the main view or **Edit > Generate report** in the main menu.

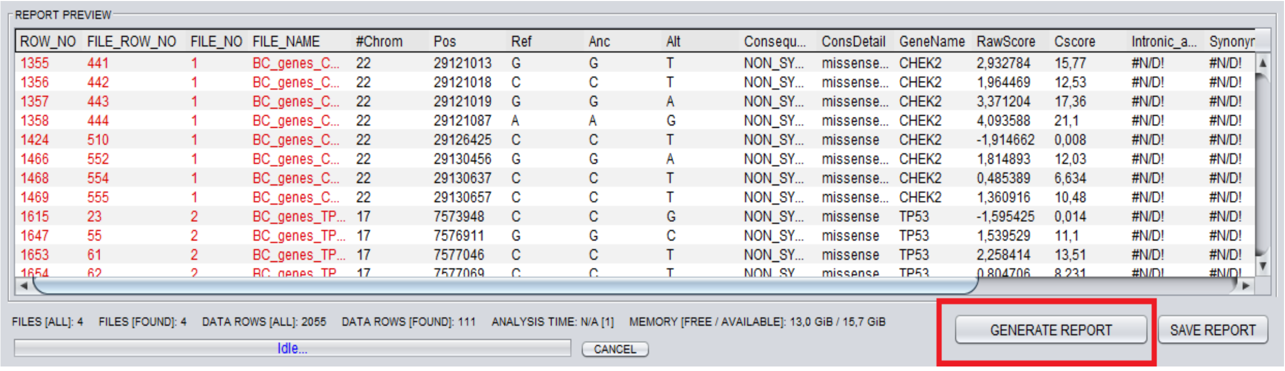

8.6 CANCELLING OF INTRODUCED CHANGES

To restore the data to the original layout, the **Reset all settings** option from the **Edit** pull down menu should be used.

It does not apply to permanently introduced changes – namely using **MODIFY CONTENT** and **RENAME** functions in **COLUMN MANAGER** window (see 8.3.1.1 COLUMN PROPERTIES subfield section) as well as all data modifications set in the **INPUT DATA PREPROCESSING** field from the **SIMPLE FILTER** tab (see **Error! Reference source not found.** INPUT DATA PREPROCESSING field).

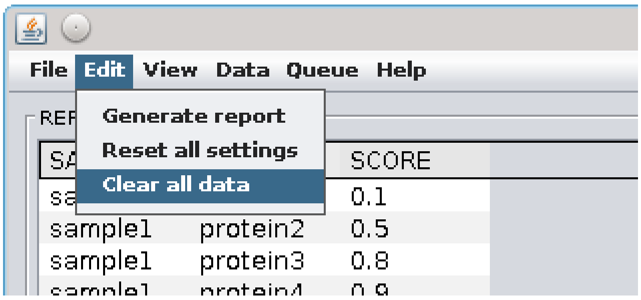

8.7 SAVING FILTERED FILE(S)

The filtered data can be saved to the output file by clicking the **SAVE REPORT** button in the main window or **File > Save Report** in pull down menu.
In the Save window the file directory and name are specified. The file is saved in tab delimited format only.

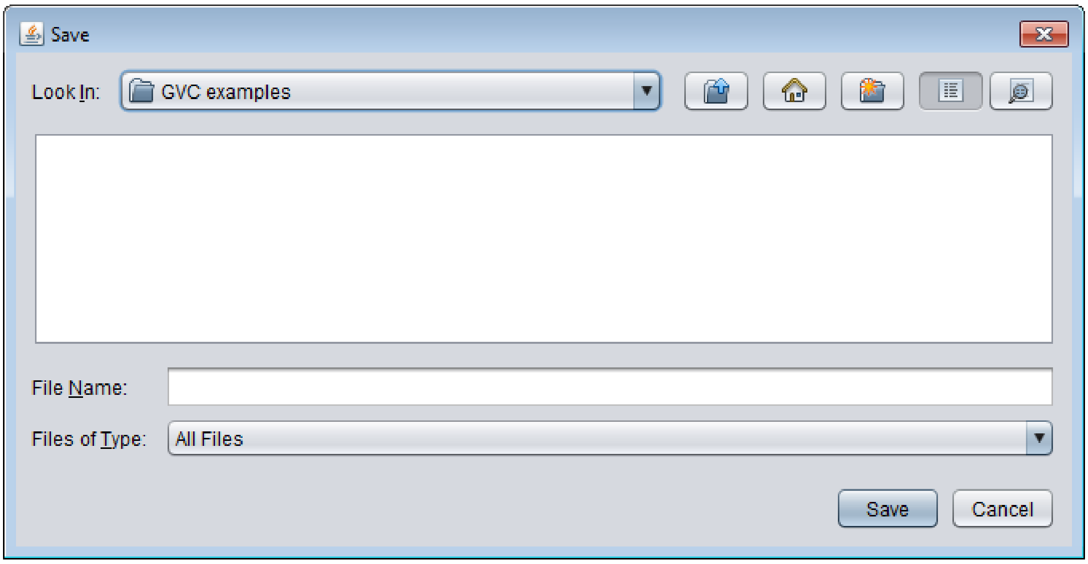

8.8 EXPORTING FILTERED FILE(S)

The filtered data can be exported to many formats (BED, BED detail, PSL, GFF, Personal Genome SNP, ENCODE RNA elements: BED6 + 3 scores, ENCODE narrowPeak: Narrow (or Point-Source) Peaks, ENCODE broadPeak: Broad Peaks (or Regions), ENCODE gappedPeak: Gapped Peaks (or Regions), ENCODE peptideMapping: BED6+4). Detailed formats description: https://genome.ucsc.edu/FAQ/FAQformat.html). Export can be done by clicking the **File > Export...** in pull down menu. Data columns should be assigned to the format-specific, required columns names before data exporting. Only assigned data columns are exported.

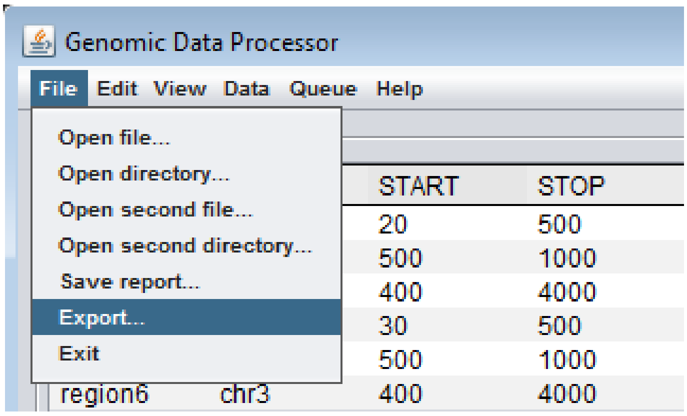

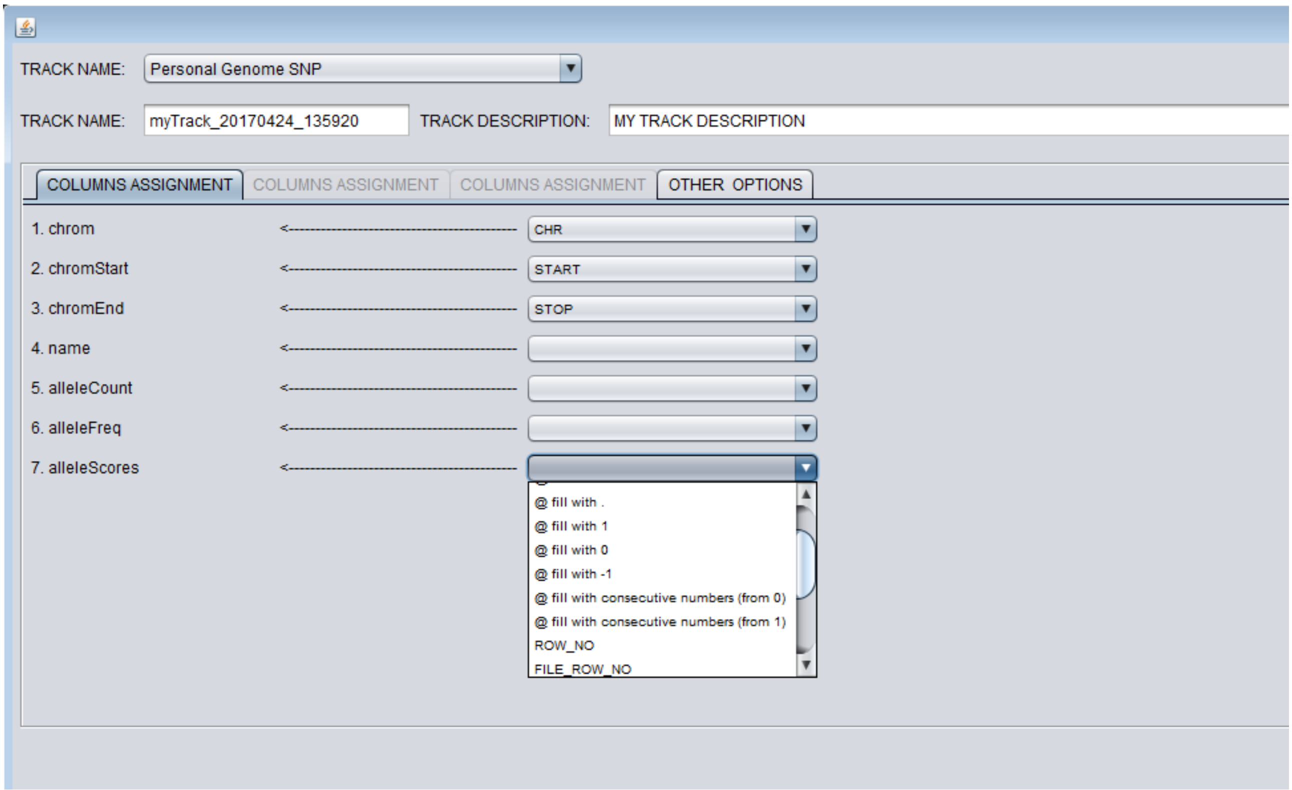

8.9 SAVING QUERIES

Every implemented set of filtering parameters can be saved for further analyses. For this purpose select **Queue** > **Save** in pull down menu in the main window. In the **Save** window the query file directory and name are specified. The file is saved in the internal format (.que extension).

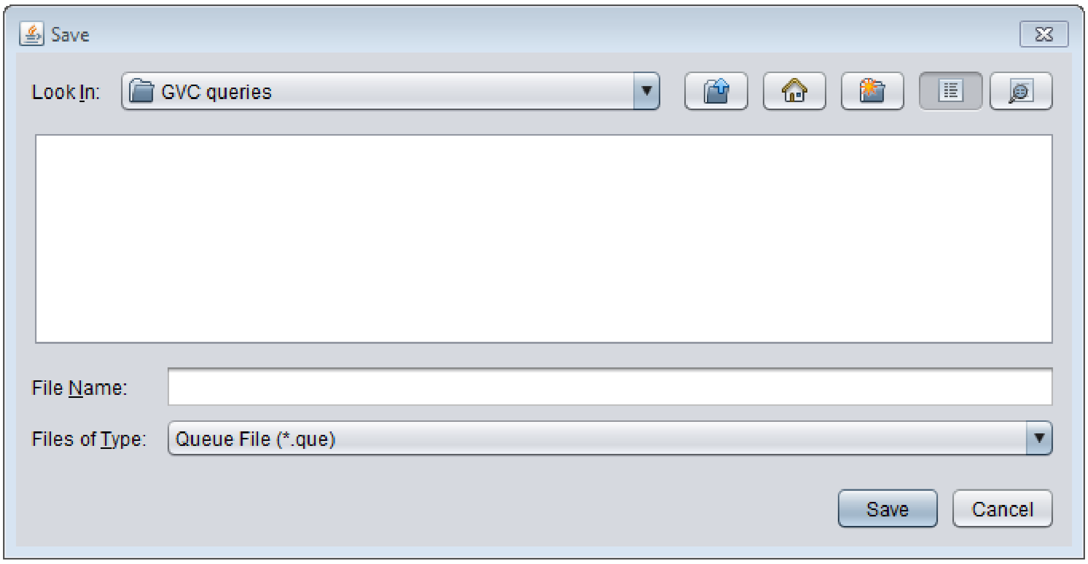

8.10 LOADING QUERIES

Previously saved query can by loaded by selecting **Queue > Load**. The Load queue file window is opened to point to the particular directory with .que file.
To successfully use the queue file, it should be consistent with previously loaded data, particularly in terms of columns' names.

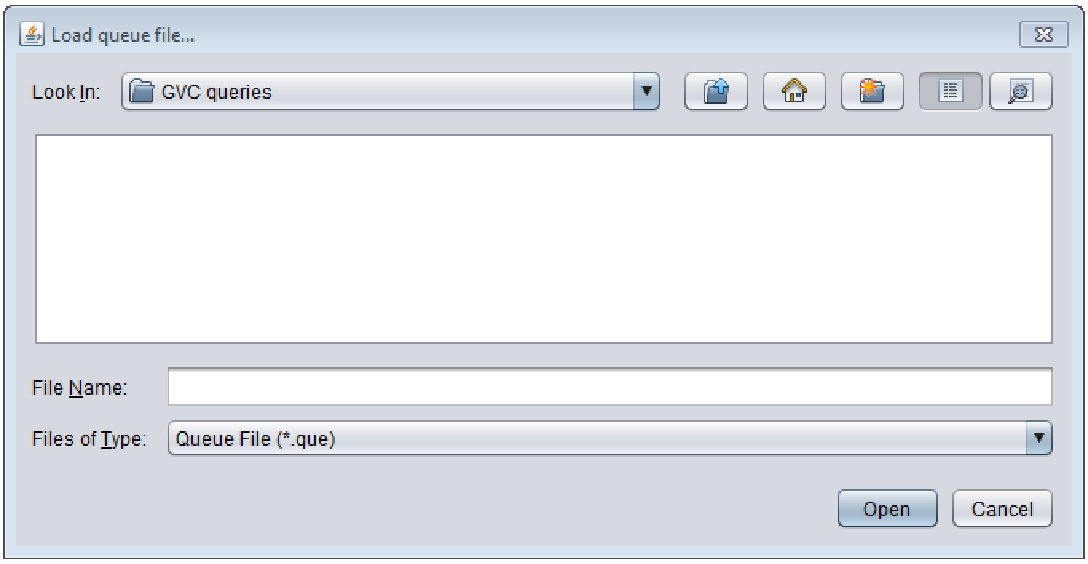

Supplement: S1 File — (PDF) [file pone.0192858.s001.pdf]
