# Supplementary material for: High-Throughput Tabular Data Processor – Platform independent graphical tool for processing large data sets
Source: PLoS One. 2018 Feb 12;13(2):e0192858. doi: 10.1371/journal.pone.0192858 (PMC5809091; doi:10.1371/journal.pone.0192858)
Supplement: S2 File — (PDF) [file pone.0192858.s002.pdf]

S2 File. Examples description

Examples repository: https://osf.io/pw2dx/
Files needed by each example were placed in example directories:

- *Example_1*
- *Example_2*
- *Example_3*
- *Example_4*
- *Example_5*

Each example directory contains subdirectories: *input_data*, *queries* and *results.*
Subdirectory *input_data* contains all input data needed to do all example tasks.
Subdirectory *queries* contains all GVC queries used by example and may be used when user do not want to manually (or do not know how to) properly fill query forms to filter data.
Subdirectory *results* contain results for each example stage allowing to fast jump to any other example stage without need to conduct all stages (some of them are time-consuming).

Example 1 contains set of easy, unassociated, one stage tasks demonstrating unique and most useful features of software using very simple artificial dataset. It may be used as reference when conducting other examples. Datasets are so small and simple that it was possible to show input and output data in the form of color tables to make understanding more easy.
Other examples contain real, scientific datasets and are more complex (many stages) and were presented as flowcharts with descriptions and notes.

In order to make text more readable some conventions were used:

- file names and paths are written using `mono font and coloured darkgreen`.
- software menu options were *italicized and mono font was used.*
- data tables column names were in coloured purple.
- rejected data rows are ~~strikethrough~~
- stage descriptions on flowcharts are in blue rectangles and notes ale in gray rectangles.

Example 1

Directory structure:
Example_1 – main directory containing example files
Example_1/input_data/ – input files for individual stages
Example_1/results – output files for individual stages
Example_1/queries – query files for individual stages

```
.
├── input_data
│   ├── example_input_file1.txt
│   ├── example_input_file2a.txt
│   ├── example_input_file2b.txt
│   ├── example_input_file3.txt
│   ├── example_input_file4a.txt
│   └── example_input_file4b.txt
├── queries
│   ├── q1.que
│   ├── q2.que
│   ├── q3.que
│   ├── q4.que
│   ├── q5.que
│   ├── q6.que
│   ├── q7.que
│   ├── q8.que
│   └── q9.que
└── results
    ├── r1.txt
    ├── r2.txt
    ├── r3.txt
    ├── r4.txt
    ├── r5.txt
    ├── r6.txt
    ├── r7.txt
    ├── r8.txt
    └── r9.txt
```

Filtering on the base of samples (column SAMPLE) where
only proteins (column PROTEIN) present in > 50% of samples are selected

SAMPLE	PROTEIN	SCORE
sample1	protein1	0.1
sample1	protein2	0.5
sample1	protein3	0.8
sample1	protein4	0.9
sample2	protein1	0.6
sample2	protein4	0.8
sample2	protein8	1.0
sample2	protein9	0.3
sample3	protein1	0.4
sample3	protein12	0.6
sample4	protein13	0.6
sample4	protein14	1.1

SAMPLE	PROTEIN	SCORE
sample1	protein1	0.1
~~sample1~~	~~protein2~~	~~0.5~~
~~sample1~~	~~protein3~~	~~0.8~~
sample1	protein4	0.9
sample2	protein1	0.6
sample2	protein4	0.8
~~sample2~~	~~protein8~~	~~1.0~~
~~sample2~~	~~protein9~~	~~0.3~~
sample3	protein1	0.4
~~sample3~~	~~protein12~~	~~0.6~~
~~sample4~~	~~protein13~~	~~0.6~~
~~sample4~~	~~protein14~~	~~1.1~~

menu: *File -> Open File ->* `Example_1/input_data/example_input_file1.txt`
menu: *View -> Advanced mode*
menu: *Queue -> Add query -> Sample Filter:*
Sample columns: **SAMPLE**
Analysed columns: **PROTEIN**
IS PRESENT IN 50% OF SAMPLES

Note:
Query is saved as file
`Example_1/queries/q1.que`
`Saved result file is`
`Example_1/results/r1.txt`

Horizontal joining of the files on the base of one column (column PROTEIN)

MAIN DATA

	SAMPLE	PROTEIN	SCORE
*	sample1	protein1	0.1
	sample1	protein2	0.5
	sample1	protein3	0.8
	sample1	protein4	0.9
	sample2	protein15	0.6
	sample2	protein41	0.8
	sample2	protein8	1.0
	sample2	protein9	0.3
	sample3	protein51	0.4
	sample3	protein12	0.6
	sample3	protein13	0.6
	sample3	protein14	1.1

EXTERNAL DATA

PROTEIN	DESCRIPTION
protein1	desc1p1
protein1	desc2p1
protein3	descp3
protein4	descp4
protein5	descp5
protein10	descp10
protein11	descp11
protein12	descp12
protein50	descp50
protein610	descp610
protein611	descp611
protein612	descp612

*-„REPEAT DATA ROW WHEN MORE THAN ONE EXTERNAL DATA ROWS MATCH” option active

* - system columns from external data not showed

RESULT

SAMPLE	PROTEIN	SCORE	PROTEIN	DESCRIPTION
sample1	protein1	0.1	protein1	desc1p1
sample1	protein1	0.1	protein1	desc2p1
sample1	protein2	0.5		
sample1	protein3	0.8	protein3	descp3
sample1	protein4	0.9	protein4	descp4
sample2	protein1	0.6		
sample2	protein4	0.8		
sample2	protein8	1.0		
sample2	protein9	0.3		
sample3	protein51	0.4		
sample3	protein12	0.6	protein12	descp12
sample3	protein13	0.6		
sample3	protein14	1.1		

menu: *File -> Open File ->* `Example_1/input_data/example_input_file2a.txt`
menu: *File -> Open Second File ->* `Example_1/input_data/example_input_file2b.txt`
menu: *View -> Advanced mode*
menu: *Queue -> Add query -> External Data Filter/Merger*:

Select columns and condition: Columns: Column: **PROTEIN**
Select columns and condition: Columns [external data]: Column: **PROTEIN**
Select columns and condition: Condition: =
Select action: Add all columns from external data matching rows
Select action: do not filter - merge only
Select action: repeat data row when more than one external data rows match

Note:
Query is saved as file
`Example_1/queries/q2.que`
Saved result file is
`Example_1/results/r2.txt`

Horizontal joining of the files on the base of one column (column PROTEIN) with filtering

MAIN DATA

	SAMPLE	PROTEIN	SCORE
*	sample1	protein1	0.1
	sample1	protein2	0.5
	sample1	protein3	0.8
	sample1	protein4	0.9
	sample2	protein15	0.6
	sample2	protein41	0.8
	sample2	protein8	1.0
	sample2	protein9	0.3
	sample3	protein51	0.4
	sample3	protein12	0.6
	sample3	protein13	0.6
	sample3	protein14	1.1

EXTERNAL DATA

PROTEIN	DESCRIPTION
protein1	desc1p1
protein1	desc2p1
protein3	descp3
protein4	descp4
protein5	descp5
protein10	descp10
protein11	descp11
protein12	descp12
protein50	descp50
protein610	descp610
protein611	descp611
protein612	descp612

*-„REPEAT DATA ROW WHEN MORE THAN ONE EXTERNAL DATA ROWS MATCH” option active

SAMPLE	PROTEIN	SCORE	PROTEIN	DESCRIPTION
sample1	protein1	0.1	protein1	desc1p1
sample1	protein1	0.1	protein1	desc2p1
sample1	protein3	0.8	protein3	descp3
sample1	protein4	0.9	protein4	descp4
sample3	protein12	0.6	protein12	descp12

menu: *File -> Open File ->* `Example_1/input_data/example_input_file2a.txt`
menu: *File -> Open Second File ->* `Example_1/input_data/example_input_file2b.txt`
menu: *View -> Advanced mode*
menu: *Queue -> Add query -> External Data Filter/Merger*:

Select columns and condition: Columns: Column: **PROTEIN**
Select columns and condition: Columns [external data]: Column: **PROTEIN**
Select columns and condition: Condition: =
Select action: Add all columns from external data matching rows
Select action: repeat data row when more than one external data rows match

Note:
Query is saved as file
`Example_1/queries/q3.que`
`Saved result file is`
`Example_1/results/r3.txt`

Filtering one file using another file on the base of one column (column PROTEIN) without joining

MAIN DATA

	SAMPLE	PROTEIN	SCORE
*	sample1	protein1	0.1
	sample1	protein2	0.5
	sample1	protein3	0.8
	sample1	protein4	0.9
	sample2	protein15	0.6
	sample2	protein41	0.8
	sample2	protein8	1.0
	sample2	protein9	0.3
	sample3	protein51	0.4
	sample3	protein12	0.6
	sample3	protein13	0.6
	sample3	protein14	1.1

EXTERNAL DATA

PROTEIN	DESCRIPTION
protein1	desc1p1
protein1	desc2p1
protein3	descp3
protein4	descp4
protein5	descp5
protein10	descp10
protein11	descp11
protein12	descp12
protein50	descp50
protein610	descp610
protein611	descp611
protein612	descp612

*-„REPEAT DATA ROW WHEN MORE THAN ONE EXTERNAL DATA ROWS MATCH" option active

SAMPLE	PROTEIN	SCORE
sample1	protein1	0.1
sample1	protein3	0.8
sample1	protein4	0.9
sample3	protein12	0.6

menu: *File -> Open File ->* `Example_1/input_data/example_input_file2a.txt`
menu: *File -> Open Second File ->* `Example_1/input_data/example_input_file2b.txt`
menu: *View -> Advanced mode*
menu: *Queue -> Add query -> External Data Filter/Merger*:

Select columns and condition: Columns: Column: **PROTEIN**
Select columns and condition: Columns [external data]: Column: **PROTEIN**
Select columns and condition: Condition: =
Select action: Filter only

Note:
Query is saved as file
`Example_1/queries/q4.que`
`Saved result file is`
`Example_1/results/r4.txt`

Selecting unique rows on the base of two columns (columns PROTEIN and REGION)

SAMPLE	PROTEIN	REGION
sample1	protein1	20
sample1	protein2	5
sample1	protein3	8
sample1	protein4	9
sample2	protein1	6
sample2	protein4	9
sample2	protein8	1
sample2	protein9	33
sample3	protein1	4
sample3	protein12	0
sample3	protein9	33
sample3	protein9	33

SAMPLE	PROTEIN	REGION
sample1	protein1	20
sample1	protein2	5
sample1	protein3	8
sample1	protein4	9
sample2	protein1	6
~~sample2~~	~~protein4~~	~~9~~
sample2	protein8	1
sample2	protein9	33
sample3	protein1	4
sample3	protein12	0
~~sample3~~	~~protein8~~	~~33~~
~~sample3~~	~~protein9~~	~~33~~

menu: *File -> Open File ->* `Example_1/input_data/example_input_file3.txt`
menu: *View -> Advanced mode*
menu: *Queue -> Add query -> Data preprocessing*:
Select unique rows in all data
On the base of columns: **PROTEIN**
REGION

Note:
Query is saved as file
`Example_1/queries/q5.que`
`Saved result file is`
`Example_1/results/r5.txt`

Selecting unique rows within each sample (column „SAMPLE”) on the base of two columns (PROTEIN and REGION)*.

SAMPLE	PROTEIN	REGION
sample1	protein1	20
sample1	protein2	5
sample1	protein3	8
sample1	protein4	9
sample2	protein1	6
sample2	protein4	9
sample2	protein8	1
sample2	protein9	33
sample3	protein1	4
sample3	protein12	0
sample3	protein9	33
sample3	protein9	33

SAMPLE	PROTEIN	REGION
sample1	protein1	20
sample1	protein2	5
sample1	protein3	8
sample1	protein4	9
sample2	protein1	6
sample2	protein4	9
sample2	protein8	1
sample2	protein9	33
sample3	protein1	4
sample3	protein12	0
sample3	protein9	33
~~sample3~~	~~protein9~~	~~33~~

menu: *`File -> Open File ->`* `Example_1/input_data/example_input_file3.txt`
menu: *`View -> Advanced mode`*
menu: *`Queue -> Add query -> Data preprocessing`*:

Select unique rows in each sample
Sample columns: **SAMPLE**
On the base of columns: **PROTEIN**
REGION

Note:
Query is saved as file
`Example_1/queries/q5.que`
`Saved result file is`
`Example_1/results/r5.txt`

*- the result data set is identical with selecting unique rows on the base of three columns „SAMPLE”, „PROTEIN” and „REGION”.

Result of filtering on the base of genome location

REGION	CHR	START	STOP
region1	chr1	20	500
region2	chr2	500	1000
region3	chr3	400	4000
region4	chr1	30	500
region5	chr2	500	1000
region6	chr3	400	4000

REGION	CHR	START	STOP
region1	chr1	20	500
region2	chr2	500	~~1000~~
region3	chr3	400	~~4000~~
region4	chr1	30	500
region5	chr2	500	~~1000~~
region6	chr3	400	~~4000~~

menu: *File -> Open File -> Example_1/input_data/example_input_file4a.txt*
menu: *View -> Advanced mode*
menu: *Queue -> Add query -> Location Filter*:

Select columns or create locus: Multiple Column Locus
Select columns or create locus: Chromosome column: **CHR**
Select columns or create locus: Start position column: **START**
Select columns or create locus: Stop position column: **STOP**

Locus search: Chromosome: 1
Locus search: Start pos.: 30
Locus search: Stop pos.: 500
Locus search: Length:
Locus search: Overlap at least [%]: 0 (means any overlap percent is allowed)

Note:
Query is saved as file
`Example_1/queries/q7.que`
`Saved result file is`
`Example_1/results/r7.txt`

Result of filtering on the base of genome location

REGION	CHR	START	STOP
region1	chr1	20	500
region2	chr2	500	1000
region3	chr3	400	4000
region4	chr1	30	500
region5	chr2	500	1000
region6	chr3	400	4000

REGION	CHR	START	STOP
region1	chr1	20	~~500~~
region2	chr2	500	~~1000~~
region3	chr3	400	~~4000~~
region4	chr1	30	500
region5	chr2	500	~~1000~~
region6	chr3	400	~~4000~~

menu: *`File -> Open File ->`* `Example_1/input_data/example_input_file4a.txt`
menu: *`View -> Advanced mode`*
menu: *`Queue -> Add query -> Location Filter`*:

Select columns or create locus: Multiple Column Locus
Select columns or create locus: Chromosome column: **CHR**
Select columns or create locus: Start position column: **START**
Select columns or create locus: Stop position column: **STOP**

Locus search: Chromosome: 1
Locus search: Start pos.: 30
Locus search: Stop pos.: 500
Locus search: Length:
Locus search: Overlap at least [%]: 100 (means only 100 % overlap are selected)

Note:
Query is saved as file
`Example_1/queries/q8.que`
`Saved result file is`
`Example_1/results/r8.txt`

Horizontal joining of the files on the base of genome location

MAIN DATA

REGION	CHR	START	STOP
region1	chr1	20	500
region2	chr2	500	1000
region3	chr3	400	4000
region4	chr1	30	500
region5	chr2	500	1000
region6	chr3	400	4000

EXTERNAL DATA

FEATURE	CHR	START	STOP	DESCRIPTION
promoterX	chr1	10	200	Description of promotor X...
regulatory sequenceY	chr1	100	1000	Description of regulatory sequence Y...
promoterZ	chr2	10	100	Description of promotor Y...
regulatory sequenceQ	chr2	390	460	Description of regulatory sequence Q...
promoterW	chr3	10	200	Description of promotor Y...
regulatory sequenceW	chr3	10	300	Description of regulatory sequence Q...

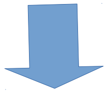

REGION	CHR	START	STOP	FEATURE	DESCRIPTION	external_data_found_rows
region1	chr1	20	500	promoterX	Description of promotor X...	2
region4	chr1	30	500	promoterX	Description of promotor X...	2
region1	chr1	20	500	regulatory sequenceY	Description of regulatory sequence Y...	2
region4	chr1	30	500	regulatory sequenceY	Description of regulatory sequence Y...	2

menu: *File -> Open File ->* `Example_1/input_data/example_input_file4a.txt`
menu: *File -> Open Second File ->* `Example_1/input_data/example_input_file4b.txt`
menu: *View -> Advanced mode*
menu: *Queue -> Add query -> External Data Filter /Merger (Location)*:

Select columns or create locus: Multiple Column Locus
Select columns or create locus: Chromosome column: **CHR**
Select columns or create locus: Start position column: **START**
Select columns or create locus: Stop position column: **STOP**

Select columns or create locus (external data): Multiple Column Locus
Select columns or create locus (external data): Chromosome column: **CHR**
Select columns or create locus (external data): Start position column: **START**
Select columns or create locus (external data): Stop position column: **STOP**

Condition: overlaps at least 0% of main data location

Select action: ADD SELECTED COLUMNS FROM EXTERNAL DATA MATCHING ROWS
Select columns: FEATURE
DESCRIPTION

„REPEAT DATA ROW WHEN MORE THAN ONE EXTERNAL DATA ROWS MATCH” option active
„ADD BASIC STATISTICS” option active

Note:
Query is saved as file
`Example_1/queries/q9.que`
Saved result file is
`Example_1/results/r9.txt`

Vertical joining of the files with partially the same column names

REGION	CHR	START	STOP
region1	chr1	20	500
region2	chr2	500	1000
region3	chr3	400	4000
region4	chr1	30	500

REGION	CHR	START	STOP	DESCRIPTION
region1	chr1	2000	50000	DESC1
region2	chr2	50000	100000	DESC2
region3	chr3	40000	400000	DESC3
region4	chr1	3000	50000	DESC4

menu: *File -> Open directory ->*
Example_1/input_data/input_files5a5b
menu: Data -> *Show system columns* (unchecked)

REGION	CHR	START	STOP	DESCRIPTION
region1	chr1	20	500	
region2	chr2	500	1000	
region3	chr3	400	4000	
region4	chr1	30	500	
region1	chr1	2000	50000	DESC1
region2	chr2	50000	100000	DESC2
region3	chr3	40000	400000	DESC3
region4	chr1	3000	50000	DESC4

Note:
Saved result file is
Example_1/results/r10.txt

Example 2

Directory structure:
Example_2 – main directory containing example files
Example_2/input_data/platypus_vcf – vcf files produced by Platypus (http://www.well.ox.ac.uk/platypus)
Example_2/input_data/seattleseq134 – files produced by SeattleSeq (http://snp.gs.washington.edu/)
Example_2/results – output files for individual stages
Example_2/queries – query files for individual stages

```
.
├── input_data
│   ├── intervals_with_col_headers.bed
│   ├── platypus_vcf
│   │   ├── AP-1_aln_n30_N100_0x0004_filtered_sorted.bam.vcf
│   │   ├── AP-2_aln_n30_N100_0x0004_filtered_sorted.bam.vcf
│   │   ├── AP-3_aln_n30_N100_0x0004_filtered_sorted.bam.vcf
│   │   ├── AP-4_aln_n30_N100_0x0004_filtered_sorted.bam.vcf
│   │   ├── AP-5_aln_n30_N100_0x0004_filtered_sorted.bam.vcf
│   │   ├── AP-6_aln_n30_N100_0x0004_filtered_sorted.bam.vcf
│   │   ├── AP-7_aln_n30_N100_0x0004_filtered_sorted.bam.vcf
│   │   └── AP-8_aln_n30_N100_0x0004_filtered_sorted.bam.vcf
│   └── seattleseq134
│       ├── SeattleSeqAnnotation134.AP-1_aln_n30_N100_0x0004_filtered_sorted.bam.vcf.245647667364.txt
│       ├── SeattleSeqAnnotation134.AP-1_aln_n30_N100_0x0004_filtered_sorted.bam.vcf.245705740173.txt
│       ├── SeattleSeqAnnotation134.AP-2_aln_n30_N100_0x0004_filtered_sorted.bam.vcf.245649049586.txt
│       ├── SeattleSeqAnnotation134.AP-2_aln_n30_N100_0x0004_filtered_sorted.bam.vcf.245706569206.txt
│       ├── SeattleSeqAnnotation134.AP-3_aln_n30_N100_0x0004_filtered_sorted.bam.vcf.245650093533.txt
│       ├── SeattleSeqAnnotation134.AP-3_aln_n30_N100_0x0004_filtered_sorted.bam.vcf.245710002010.txt
│       ├── SeattleSeqAnnotation134.AP-4_aln_n30_N100_0x0004_filtered_sorted.bam.vcf.245650643836.txt
│       ├── SeattleSeqAnnotation134.AP-4_aln_n30_N100_0x0004_filtered_sorted.bam.vcf.245712178025.txt
│       ├── SeattleSeqAnnotation134.AP-5_aln_n30_N100_0x0004_filtered_sorted.bam.vcf.245651221106.txt
│       ├── SeattleSeqAnnotation134.AP-5_aln_n30_N100_0x0004_filtered_sorted.bam.vcf.245714132870.txt
│       ├── SeattleSeqAnnotation134.AP-6_aln_n30_N100_0x0004_filtered_sorted.bam.vcf.245652094290.txt
│       ├── SeattleSeqAnnotation134.AP-6_aln_n30_N100_0x0004_filtered_sorted.bam.vcf.245716307661.txt
│       ├── SeattleSeqAnnotation134.AP-7_aln_n30_N100_0x0004_filtered_sorted.bam.vcf.245652712335.txt
│       ├── SeattleSeqAnnotation134.AP-7_aln_n30_N100_0x0004_filtered_sorted.bam.vcf.245716589511.txt
│       ├── SeattleSeqAnnotation134.AP-8_aln_n30_N100_0x0004_filtered_sorted.bam.vcf.245653191154.txt
│       └── SeattleSeqAnnotation134.AP-8_aln_n30_N100_0x0004_filtered_sorted.bam.vcf.245717027188.txt
├── results
│   ├── st1.txt
│   ├── st2.txt
│   ├── st3.txt
│   ├── st4a.txt
│   ├── st4b.txt
│   ├── st5_sel_columns.txt
│   └── st5.txt
└── queries
    ├── st1.que
    ├── st2.que
    ├── st3.que
    ├── st4a.que
    ├── st4b1.que
    ├── st4b.que
    └── st5.que
```

1. Open all files in directory `Example_2/input_data/platypus_vcf` *(menu: File > Open directory...)*

2. Use file `Example_2/input_data/intervals_with_headers.bed` as external data *(menu: File > Open second file...)* and select only variants within intervals *(menu: Queue > Add query > External data filter/merger (location))*

Note:
The length of variants should be determined by REF column data cells strings lengths because no variant length are provided.
Query is saved as file `Example_2/queries/st1.que`
Saved result file is `Example_2/results/st1.txt`

3. Save results as st1.txt (with system columns) *(menu: File > Save report...)*

Note:
Results should be the same as data presented in Supplementary Table 2: Depth of coverage of LZTR1 on 22q and variant calling statistics in publication „Germline loss-of-function mutations in LZTR1 predispose to an inherited disorder of multiple schwannomas", (table row: „Annotated entries in ROIs")

4. Open all files in directory `Example_2/input_data/seattleseq134/` *(menu: File > Open directory...)*

5. Select only rows with string „none" in column #inDBSNPOrNOT

Note:
Query is saved as file `Example_2/queries/st2.que`
Saved result file is `Example_2/results/st2.txt`

6. Save results as `st2.txt` (with system columns) *(menu: File > Save report...)*

Note:
Results should be the same as data presented in Supplementary Table 2: Depth of coverage of LZTR1 on 22q and variant calling statistics in publication „Germline loss-of-function mutations in LZTR1 predispose to an inherited disorder of multiple schwannomas", (table row: „Annotated entries not in dbSNP build 134".)

7. Open saved file `st2.txt` and filter using list of values:
   - missense
   - stop-gained
   - frameshift
   - coding

   Select only rows containing any of these values in the column function gvs *(menu: Queue > Add query > Simple filter)*

Note:
Results should be the same as data presented in Supplementary Table 2: Depth of coverage of LZTR1 on 22q and variant calling statistics in publicationi „Germline loss-of-function mutations in LZTR1 predispose to an inherited disorder of multiple schwannomas", wiersz „Missense, nonsense,deletion entries (Variants)"
Query is saved as file `Example_2/queries/st3.que`

8. Save results as st3.txt *(menu: File > Save report...)*

9. Merge files: st4b.txt and st4a.txt using genome localization and sample name as joining columns *(menu: Queue > Add query > External Data Filter/Merger)*. Save results as `st5.txt`.

Note:
The sample names unification in both files is required before joining (column file_name##).
(menu: Queue > Add query > Simple filter > input data preprocessing)
The sample names should be in scheme AP-[number], eg. AP-3. Any other substrings should be removed so eg. string "SeattleSeqAnnotation134.AP-1_aln_n30_N100_0x0004_filtered_sorted.bam.vcf.245647667364.txt" should be reduced to "AP-1". The operation may be accomplished using cutting or replacing options in "Simple filter" tab of "Add query" window ("input data preprocessing" form).
Similar task should be done for columns with chromosome number (the column should contain only chromosome numbers without "chr" prefix in both files). Unified data files were saved:
`Example_2/results/st1.txt was saved as Example_2/results/st4a.txt`
`Example_2/results/st3.txt was saved as Example_2/results/st4b.txt`
Unification queries were saved as files:
`Example_2/queries/st4a1.que` and `Example_2/queries/st4a2.que` (for st4a.txt data file)
`Example_2/queries/st4b.que` (for st4b.txt data file).
Joining query was saved as st5.que.

10. Open saved results and hide unwanted columns in Column Manager *(menu: Data > Column manager)* (hide all columns added from `st4a.txt` file with exception of columns names starting with #filter or #info).

Save results as `st5_sel_columns.txt` *(menu: File > Save report...)*

Example 3

Directory structure:
Example_3 – main directory containing example files
Example_3/input_data/conservation_all_headers_corrected/
– evolutionary conservation files (subdirectory chr_col_added contain the same files extended by required columns with chromosome name)
Example_3/results – output files for individual stages
Example_3/queries – query files for individual stages

```
.
├── input_data
│   ├── conservation_all_headers_corrected
│   │   ├── chr22_16050001-26657233_phastVertebrateCons46way.txt
│   │   ├── chr22_26657234-36737763_phastVertebrateCons46way.txt
│   │   ├── chr22_36737764-46804985_phastVertebrateCons46way.txt
│   │   ├── chr22_46804986-51304566_phastVertebrateCons46way.txt
│   │   └── chr_col_added
│   │       ├── 2_chr22_16050001-26657233_phastVertebrateCons46way.txt
│   │       ├── 2_chr22_26657234-36737763_phastVertebrateCons46way.txt
│   │       ├── 2_chr22_36737764-46804985_phastVertebrateCons46way.txt
│   │       └── 2_chr22_46804986-51304566_phastVertebrateCons46way.txt
│   ├── indels_snvs_z_kol_sys.txt
│   ├── tgpPhase3AccessibilityPilotCriteria.txt
│   └── tgpPhase3AccessibilityStrictCriteria.txt
├── results
│   ├── st1_report_pilot.txt
│   ├── st1_report_strict.txt
│   ├── st2_report_pilot.txt
│   ├── st2_report_strict.txt
│   ├── st3_report_pilot.txt
│   └── st3_report_strict.txt
└── queries
    ├── st1.que
    ├── st2.que
    ├── st3.que
    └── st4.que
```

1. Open main data file:
`/Example_3/indels_snvs_z_kol_sys.txt`
(menu: File > Open file...)
(menu: View > Advanced mode)

2. Open second data file:
`/Example_3/input_data/tgpPhase3AccessibilityPilotCriteria.txt`
(menu: File > Open second file...)

3. Filter `indels_snvs_z_kol_sys.txt` file selecting rows with genetic changes at least partially covering locations from file `tgpPhase3AccessibilityPilotCriteria.txt`
(menu: Queue > Add query > External data filter/merger (location))
Save results as `st1_pilot_report.txt`
(menu: File > Save report...)

Note:
Query is saved as file `Example_3/queries/st1.que`

Note:
The stop position of genetic changes in file indels_snvs_z_kol_sys.txt should be determined by referenceBase column data cells strings lengths because no variant length are provided.

4. Open main data file:
`/Example_3/input_data/indels_snvs_z_kol_sys.txt`
(menu: File > Open file...)
(menu: View > Advanced mode)

5. Open second data file:
`/Example_3/input_data/tgpPhase3AccessibilityStrictCriteria.txt`
(menu: File > Open second file...)

6. Filter `indels_snvs_z_kol_sys.txt` file selecting rows with genetic changes at least partially covering locations from file `tgpPhase3AccessibilityStrictCriteria.txt`
(menu: Queue > Add query > External data filter/merger (location))
Save results as `st1_strict_report.txt`
(menu: File > Save report...)

Note:
Query is saved as file `Example_3/queries/st2.que`

7. Add column with genetic conservation to results of previous steps using files downloaded from UCSC Genome Browser and modified accordingly to description in note. The procedure described below should be repeated for two results files (`st1_pilot_report.txt` and `st1_strict_report.txt`):
(menu: File > Open file...) (st1_pilot_report.txt or st1_strict_report.txt)
(menu: File > Open second directory...) (genetic conservation files directory: `/Example_3/input_data/conservation_all_headers_corrected/chr_col_added/`
(menu: Queue > Add query > External data filter/merger (location))
Save result files:
`st2_pilot_report.txt` and `st2_strict_report.txt`
(menu: File > Save report...)

Note:
The genetic conservation files need to be modified before joining. The header should be one row and additional column with chromosome number should be added. Header correction can be made manually in any capable text editor. Additional row can be added eg. using linux command: awk '$0="chr[chr number]/+"$0"[input_file] > [output_file].
The corrected files are placed in directory:
`/Example_3/input_data/conservation_all_headers_corrected/chr_col_added/`

Note:
In the case of more than one row the mean of genetic conservation is calculated.

Note:
Query is saved as file `Example_3/queries/st3.que`

8. Open saved `st2_pilot_report.txt` and `st2_strict_report.txt` and filter out rows with < 0.85 genetic conservation factor.
(menu: Queue > Add query > Simple filter)
Save the result files (`st3_pilot_report.txt` and `st3_strict_report.txt`) *(menu: File > Save report...)*

Note:
Query is saved as file `Example_3/queries/st4.que`

Example 4

Directory structure:
Example_4 – main directory containing example files
Example_4/input_data/vcf – vcf files
Example_4/results – output files for individual stages
Example_4/saved_queries – query files for individual stages

```
.
├── input_data
│   ├── Supporting_Table_S2.csv
│   └── vcf
│       ├── 608P.bam_PASS_filter_only.txt.vcf
│       ├── 642P.bam_PASS_filter_only.txt.vcf
│       ├── 903P_PASS_filter_only.bam.vcf
│       ├── 915P.bam_PASS_filter_only.txt.vcf
│       ├── 916P.bam_PASS_filter_only.txt.vcf
│       └── 924P_PASS_filter_only.bam.vcf
├── queries
│   ├── st1.que
│   ├── st2.que
│   └── st3.que
└── results
    ├── final
    │   ├── 608h_608h.txt
    │   ├── 608h_608m.txt
    │   ├── 608h_608t.txt
    │   ├── 608h_all.txt
    │   ├── 608h_null.txt
    │   ├── 642h_642h.txt
    │   ├── 642h_642m.txt
    │   ├── 642h_642t.txt
    │   ├── 642h_all.txt
    │   ├── 642h_null.txt
    │   ├── 903h_903m.txt
    │   ├── 903h_903t.txt
    │   ├── 903h_all.txt
    │   ├── 903h_null.txt
    │   ├── 915h_915h.txt
    │   ├── 915h_915m.txt
    │   ├── 915h_915t.txt
    │   ├── 915h_all.txt
    │   ├── 915h_null.txt
    │   ├── 916h_916h.txt
    │   ├── 916h_916m.txt
    │   ├── 916h_916t.txt
    │   ├── 916h_all.txt
    │   ├── 916h_null.txt
    │   ├── 924h_924h.txt
    │   ├── 924h_924m.txt
    │   ├── 924h_924t.txt
    │   ├── 924h_all.txt
    │   └── 924h_null.txt
    ├── st1.txt
    ├── st2.txt
    ├── st3.txt
    └── Supporting_Table_S2_st1.txt
```

1 Open all files in directory `Example_4/input_data/vcf`
(menu: File > Open directory...).
Save result file as `st1.txt`

2 The sample names unification is required in column vcf_sample_name (see note). Save result file as `st2.txt`
(menu: File > Save report...)

Note:
The sample names unification in `st1.txt` file is required (column vcf_sample_name).
(menu: Queue > Add query > Simple filter > input data preprocessing)
The sample names should be in scheme [number]H, eg. 924H. Any other substrings should be removed so eg. string "galaxy41-[ap924p_sam-to-bam_on_data_23__converted_bam]" should first be reduced to "924p" and next the letter "p" in resultant sample name string should be replaced by letter "H" giving string "924H". The operation may be accomplished using replacing options in "Simple filter" tab of "Add query" window ("input data preprocessing" form).
(menu: Queue > Add query > Simple filter)
Unification queries were saved as file:
`Example_4/queries/st1.que`

3 Open file `Example_4/input_data/Supporting_Table_S1.csv`
Remove numbers "1" or "1,2" at the end of strings in column patient so that cell content was eg. 924T instead 924T1,2
(menu: View > Advanced mode)
(menu: Queue > Add query > Simple filter)
Save result file as `Supporting_Table_S2_st1.txt`
(menu: File > Save report...)

Note:
Query is saved as file
`Example_4/queries/st2.que`

4 Open file `st2.txt` as main data
(menu: View > Advanced mode)
(menu: File > Open file...)
Open file `Supporting_Table_S2_st1.txt` as external data
(menu: File > Open second file...)
Merge the files using genome localization.
(menu: Queue > Add query > External data filter/merger (location)).
Save result file as `st3.txt`
(menu: File > Save report...)

Note:
Query is saved as file
`Example_4/queries/st3.que`

5 Open file `st3.txt`
Each sample need operation described below (on the example of sample 608H)
(menu: View > Basic mode)
(button: Filter)
Select only rows containing string "608H" in column vcf_sample_name
(button: Filter)
Save result file `608h_all.txt`
Open result file `608h_all.txt`
(button: Filter)
Select only rows containing string "608H" in column patient
(button: Filter)
Save result file `608h_608h.txt`
Select only rows containing string "608T" in column patient
(button: Filter)
Save result file `608h_608t.txt`
Select only rows containing string "608M" in column patient
(button: Filter)
Save result file `608h_608m.txt`
Select only rows containing string "null" in column patient
(button: Filter)
Save result file `608h_null.txt`

Note:
Saved result files are in directory
`Example_4/results/final/`

Example 5

Directory structure:
Example_5 – main directory containing example files
Example_5/results – output files for individual stages

```
.
├── input_data
│   ├── 1A_S1_hg38.bam.vcf
│   ├── 540237_41071_Area1_2012-06-25_25000bp_avg_segMNT.gff
│   └── 540237_41071_Area1_2012-06-25_unavg_segMNT.gff
└── results
    ├── 1A_S1_hg38.bam.txt
    ├── 540237_41071_Area1_2012-06-25_25000bp_avg_segMNT_s1.gff
    └── 540237_41071_Area1_2012-06-25_unavg_segMNT_s1.gff
```

❶

Open file `Example_5/input_data/1A_S1_hg38.bam.vcf`
Remove all columns with exception:

#_CHROM
POS
ID
REF
ALT
QUAL
FILTER
INFO@_TC
INFO@_TR

(menu: Data > Column manager)
Uncheck check boxes near the names of columns which should be deleted
Save result file as `1A_S1_hg38.bam.txt`
(menu: File > Save report...)

Note:
The result file is saved as tab delimited text file.
The result file size was reduced to 14,6% of input file size.

❷

Open file `Example_5/input_data/540237_41071_Area1_2012-06-25_25000bp_avg_segMNT.gff`
Remove columns NimbleScan:segMNT and 540237_41071_Area1_2012-06-25_25000bp_segMNT
(menu: Data > Column manager)
Uncheck check boxes near the names of columns
Save result file as `540237_41071_Area1_2012-06-25_25000bp_avg_segMNT_s1.gff`
(menu: File > Save report...)

Note:
The result file size was reduced to 43,5% of input file size.

❸

Open file `Example_5/input_data/540237_41071_Area1_2012-06-25_unavg_segMNT.gff`
Remove columns NimbleScan:segMNT and 540237_41071_Area1_2012-06-25_25000bp_segMNT
(menu: Data > Column manager)
Uncheck check boxes near the names of columns
Save result file as `540237_41071_Area1_2012-06-25_unavg_segMNT_s1.gff`
(menu: File > Save report...)

Note:
The result file size was reduced to 36% of input file size.

Note:
These files does not contain column headers so the first row is treated as column headers row. This fact do not affect the actual results.

Table 1. **Software/command equivalents for sample tasks that can performed with HTDP to achieve the same goal.**

There are many command line tools, software packages and programming languages that provide alternative ways to perform complex operations on text files with the same results. Many of them are native to linux/unix systems. The table below briefly presents a choice of the most obvious methods to achieve the analogous outcome as the results that were delivered by HTDP in examples as described in the paper (S2 file) (https://osf.io/pw2dx/). The file names used are real and can be found in „input data” or „results” subfolders of the relevant example. Despite availability of many ready-to-use tools, some stages of data processing are difficult to achieve using relatively short commands - in such cases writing specific scripts is necessary. All presented examples print results to the standard output which may redirected to a file with '> output_file_name.txt' string added at the end of command.

EXAMPLE NO<br>AND STAGE NO	COMMAND/SOFTWARE	NOTES
1<br>Filtering on the basis of samples (column SAMPLE) where<br>only proteins (column PROTEIN) present in > 50% of samples are selected	custom script	This task may be carried out using many programing languages (bash script, perl, php, using sql database depending on data amount). The script should make an array of proteins from column „PROTEIN” and samples from column „SAMPLE”, count the percentage of presence of each protein in each sample and select only proteins meeting the critera and next select only rows containing names of selected proteins.
1<br>Horizontal joining of the files on the basis of one column (column PROTEIN)<br>with filtering and without filtering	join --header -a 1 -1 2 -2 1 <(sort -k 2,2 example_input_file2a.txt) <(sort -k 1,1 example_input_file2b.txt)<br><br>join --header -1 2 -2 1 <(sort -k 2,2 example_input_file2a.txt) <(sort -k 1,1 example_input_file2b.txt)	join - bash command writing to standard output a line for each pair of input lines that have identical join fields.
1<br>Filtering one file using another file on the basis of one column<br>(column PROTEIN) without joining	awk 'NR==FNR{pats[$1]; next} $2 in pats' example_input_file2b.txt example_input_file2a.txt	awk command searches files for text containing a pattern. When a line or text matches, awk performs a specific action on that line/text.
1<br>Selecting unique rows on the basis of two columns<br>(columns PROTEIN and REGION)	sort -u -k2,3 example_input_file3.txt<br><br>awk -F"\t" '!seen[$2, $3]++' example_input_file3.txt	sort command - write sorted concatenation of all file(s) to standard output.
1<br>Selecting unique rows within each sample (column „SAMPLE”) on the basis of two columns (PROTEIN and REGION)*.	sort -u -k1,3 example_input_file3.txt<br><br>awk -F"\t" '!seen[$2, $3]++' example_input_file3.txt	-

EXAMPLE NO<br>AND STAGE NO	COMMAND/SOFTWARE	NOTES
1<br>Filtering on the basis of genome location<br><br>Horizontal joining of the files on the basis of genome location	custom script	This task may be carried out using many programming languages (bash script, perl, php, using sql database depending on data amount). The script should first associate columns with genomic location information fields: chromosome start and end position. All rows with unwanted chromosomes may be filtered out in first step (eg. join command) and next the rows are selected by comparing numbers in start and end positions
1<br>Vertical joining of the files with partially the same column names	custom script	This task may be carried out using many programming languages (bash script, perl, php). The simplest method is to read each file to multidimensional arrays storing data from each column and then organize data by joining the data from columns with the same name.
2<br>stages 1, 4	custom script	the same as in example 1 - Vertical joining of the files
2<br>stages: 2, 3	custom script	the same as example 1 - Horizontal joining of the files on the basis of genomic location
2<br>stage: 5, 6	awk 'NF' *.txt > test.csv<br>sed '/^#/ d' < test.csv<br>awk -v ss='none' '$1 == ss' test.csv	Vertical joining of the files (awk), deleting comment rows starting with '#' (sed) , simple filtering of rows on the basis of selected column value (awk)
2<br>stage: 7, 8	awk '{if ($13=="missense" || $13=="stp-gained" || $13=="frameshift" || $13=="coding") print}' st2.txt	filtering on the basis of list of values
2<br>stage: 9	awk '{print $28$29$1, $0}' st4a.txt > st4a_.txt<br>awk '{print $5$6$7, $0}' st4b.txt > st4b_.txt<br><br>join --header -a 1 -1 1 -2 1 <(sort -k 1,1 st4a_.txt) <(sort -k 1,1 st4b_.txt)	joining on the basis of three columns requires creating of additional column with combined values (awk) and then standard joining using join command
2<br>stage: 10	awk '{print $51,$52,$53,$54,$55,$56, $57,$58,$59,$60,$61,$62,$63,$64,$65,$66,$67,$68,$69,$70,$71,$72,$73,$74,$75,$76,$77,$78}' st5.txt	removing columns from the file
3<br>stages 1,2,3, 4, 5, 6	custom script	the same as in example 1 - Horizontal joining of the files on the basis of genomic location
3<br>stage: 7	custom_script	the same as in example 1 - Horizontal joining of the files on the basis of genome location, vertical joining of the files

EXAMPLE NO<br>AND STAGE NO	COMMAND/SOFTWARE	NOTES
3<br>stage: 8	awk -v threshold=0.85 '$41 > threshold' st2_report_strict.txt	simple filtering rows on the basis of selected column value > 0.85
4<br>stage: 1	vcf-merge<br>608P.bam_PASS_filter_only.txt.vcf<br>903P_PASS_filter_only.bam.vcf<br>916P.bam_PASS_filter_only.txt.vcf<br>642P.bam_PASS_filter_only.txt.vcf<br>915P.bam_PASS_filter_only.txt.vcf<br>924P_PASS_filter_only.bam.vcf	joining of vcf files. vcf-merge is included in VCFtools package
4<br>stage: 2, 3	custom script	sample names unification
4<br>stage: 4	custom script	the same as in example 1 - horizontal joining of the files on the basis of genomic location
4<br>stage: 5	awk -v ss='608H' '$37 == ss' st3.txt	simple filtering of rows on the basis of selected column value
5<br>stages: 1, 2, 3	awk '{print $1 $4 $5 $6 $7 $8 $9}' 540237_41071_Area1_2012-06-25_25000bp_avg_segMNT.gff<br><br>cut -f1,4- 540237_41071_Area1_2012-06-25_25000bp_avg_segMNT.gff	removing columns from the file<br>(vcf file need to be treated specifically - in some cases custom script may be required)
